# Multi-omics analysis reveals the molecular response to heat stress in a “red tide” dinoflagellate

**DOI:** 10.1186/s13059-023-03107-4

**Published:** 2023-11-23

**Authors:** Katherine E. Dougan, Zhi-Luo Deng, Lars Wöhlbrand, Carsten Reuse, Boyke Bunk, Yibi Chen, Juliane Hartlich, Karsten Hiller, Uwe John, Jana Kalvelage, Johannes Mansky, Meina Neumann-Schaal, Jörg Overmann, Jörn Petersen, Selene Sanchez-Garcia, Kerstin Schmidt-Hohagen, Sarah Shah, Cathrin Spröer, Helena Sztajer, Hui Wang, Debashish Bhattacharya, Ralf Rabus, Dieter Jahn, Cheong Xin Chan, Irene Wagner-Döbler

**Affiliations:** 1 Australian Centre for Ecogenomics, School of Chemistry and Molecular Biosciences, The University of Queensland, Brisbane, QLD 4072 Australia; 2 Helmholtz-Center for Infection Research (HZI), Inhoffenstraße 7, Braunschweig, 38124 Germany; 3 Institute for Chemistry and Biology of the Marine Environment (ICBM), Carl von Ossietzky University of Oldenburg, 26129 Oldenburg, Germany; 4 Braunschweig Center for Systems Biology (BRICS), Technische Universität Braunschweig, Rebenring 56, 38106 Brunswick, Germany; 5 German Culture Collection for Microorganisms and Cell Cultures (DSMZ), Inhoffenstraße 7B, 38124 Braunschweig, Germany; 6 Alfred Wegener Institute, Helmholtz Centre for Polar and Marine Research, Am Handelshafen 12, 27570 Bremerhaven, Germany; 7 Helmholtz Institute for Functional Marine Biodiversity at the University of Oldenburg (HIFMB), Ammerländer Heerstraße 231, 26129 Oldenburg, Germany; 8 Department of Biochemistry and Microbiology, Rutgers University, New Brunswick, NJ 08901 USA

**Keywords:** Genome evolution, Molecular regulation, Molecular response, Dinoflagellates, Harmful algal bloom, Heat stress

## Abstract

**Background:**

“Red tides” are harmful algal blooms caused by dinoflagellate microalgae that accumulate toxins lethal to other organisms, including humans via consumption of contaminated seafood. These algal blooms are driven by a combination of environmental factors including nutrient enrichment, particularly in warm waters, and are increasingly frequent. The molecular, regulatory, and evolutionary mechanisms that underlie the heat stress response in these harmful bloom-forming algal species remain little understood, due in part to the limited genomic resources from dinoflagellates, complicated by the large sizes of genomes, exhibiting features atypical of eukaryotes.

**Results:**

We present the de novo assembled genome (~ 4.75 Gbp with 85,849 protein-coding genes), transcriptome, proteome, and metabolome from *Prorocentrum cordatum*, a globally abundant, bloom-forming dinoflagellate. Using axenic algal cultures, we study the molecular mechanisms that underpin the algal response to heat stress, which is relevant to current ocean warming trends. We present the first evidence of a complementary interplay between RNA editing and exon usage that regulates the expression and functional diversity of biomolecules, reflected by reduction in photosynthesis, central metabolism, and protein synthesis. These results reveal genomic signatures and post-transcriptional regulation for the first time in a pelagic dinoflagellate.

**Conclusions:**

Our multi-omics analyses uncover the molecular response to heat stress in an important bloom-forming algal species, which is driven by complex gene structures in a large, high-G+C genome, combined with multi-level transcriptional regulation. The dynamics and interplay of molecular regulatory mechanisms may explain in part how dinoflagellates diversified to become some of the most ecologically successful organisms on Earth.

**Supplementary Information:**

The online version contains supplementary material available at 10.1186/s13059-023-03107-4.

## Background

Harmful algal blooms (HABs) result from highly accelerated microalgal growth that is often triggered by increasing water temperature, light intensity, and/or available nutrients. HABs often lead to oxygen depletion and toxin accumulation, causing significant losses to fisheries and aquaculture industries (~ USD 8B annual losses globally [1, 2]). Among HABs, the increasingly frequent “red tides” are caused by bloom-forming dinoflagellate microalgae, such as species of *Alexandrium*, *Amphidinium*, and *Prorocentrum* [3, 4]. Habitat expansion of bloom-forming dinoflagellates has been linked to warming oceans and global climate change [5].

Dinoflagellates are an ancient and highly diverse plankton group within the Alveolata, encompassing free-living, bloom-forming, parasitic, and symbiotic taxa [6, 7], with most species being mixotrophs (i.e., they combine phototrophic and heterotrophic modes of energy generation) or heterotrophs [8]. The photosynthetic machinery of dinoflagellates powers the biological carbon pump of oceans, which is essential for lowering the global carbon budget. With increasing water temperature, aquatic microbes are exposed to several cellular challenges at their upper tolerance level, including with high metabolic rates and membrane fluidity, while maintaining photosynthetic efficiency. For dinoflagellates, the formation of large blooms and the maintenance of beneficial symbioses with corals and other organisms are all impacted by increasing water temperature [5, 9]. Transcriptome studies of toxic dinoflagellates, e.g., [10–12], had revealed stress-related gene functions including metabolism and cell signaling. However, the molecular regulatory and evolutionary mechanisms that underlie the heat stress response in HAB-forming species remain little understood. This is primarily explained by the lack of high-quality genome data from these dinoflagellates, which may be up to ~ 200 Gbp in size [13, 14] and exhibit features atypical of eukaryotes [15, 16]. Thus far, genome studies [17–22] have targeted members of the family Symbiodiniaceae which form coral symbiosis and their free-living relatives in the genus *Polarella* (genome sizes ≤ 3 Gbp). These analyses reveal extensive sequence divergence and lineage-specific innovations with respect to putative gene functions. A single draft genome assembly exists for HAB-forming dinoflagellates, from *Amphidinium gibbosum* (~ 6.4 Gbp) [23], which was used to study metabolic and toxin biosynthesis functions vis-à-vis nutrient deprivation. Past studies lack proteome and metabolome data, which are necessary to elucidate the molecular mechanisms that underpin gene expression regulation. Although some muti-omics data were recently generated from three Symbiodiniaceae species [24], these results are not relevant to distantly related HAB species, given the high divergence that exists among dinoflagellate genomes [21, 22, 25].

Relevant to our study, transcriptional regulation is minimal in dinoflagellates [12, 16] with only a handful of known transcriptional regulators [26], and chromosomes existing in a permanently condensed, liquid crystalline state [27]. Initial studies [28, 29] suggested that most dinoflagellate genes are constitutively expressed regardless of growth conditions, particularly of shock treatments, but more-recent research suggests a potentially important role for differential gene regulation in these species [30]. *Trans*-splicing of a conserved spliced leader sequence in nuclear genes has been described [31]. Editing of mRNAs occurs for both organelle- and nuclear-encoded genes [29, 32], suggesting a role for this mechanism in generating physiological flexibility.

Among bloom-forming dinoflagellates, *Prorocentrum cordatum* (formerly *Prorocentrum minimum*) [33, 34] is an invasive, potentially toxic species that has expanded its habitat from the Caspian Sea where it was originally found to coastal oceans worldwide [35], including the temperate oceans in the North Atlantic where it is now regularly detected [36]. The tolerance of *P. cordatum* to a wide range of salinities and temperatures facilitates its increased bloom frequency [33, 34]. Along with the estimated 1.5°C rise in average sea temperatures, the period when temperatures range between 26 and 30°C at certain depths and areas of the oceans is expected to be prolonged in future [37]. Here, we present the genome and multi-omics data from *P. cordatum*, targeting the algal heat stress response in axenic cultures. Our results provide an integrated view of how a HAB-forming species may respond to ocean warming induced by global climate change.

## Results

### *P. cordatum* genome reveals hallmarks of bloom-forming dinoflagellates

**Fig. 1:**
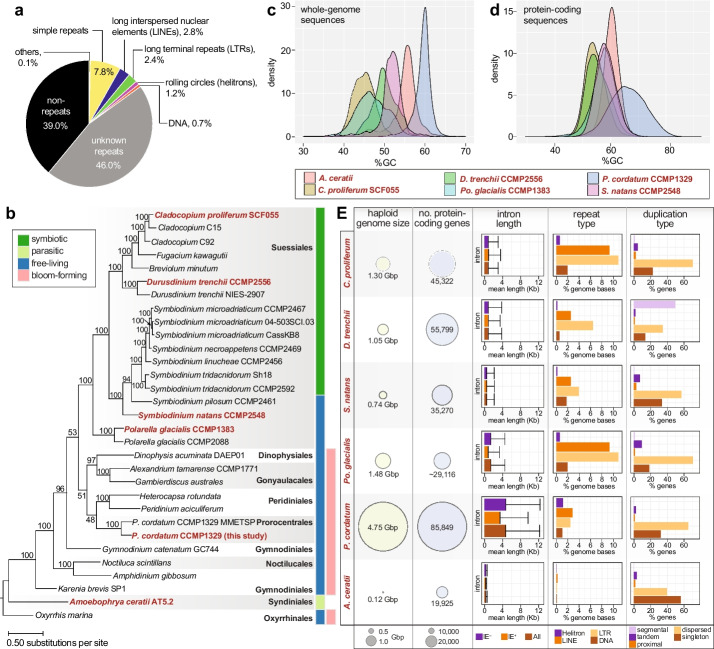
Genome features of *P. cordatum*. **a** Distribution of repeat types in the *P. cordatum* genome. **b** Maximum likelihood tree inferred using 3507 strictly orthologous, single-copy protein sets among 31 dinoflagellate taxa, with ultrafast bootstrap support (based on 2000 replicate samples) shown at each internal node; unit of branch length is number of substitutions per site. The ecological niche for each taxon is shown on the right of the tree. The five representative taxa and *P. cordatum* from this study are highlighted on the tree in red text. Distribution of G+C content for **c** whole-genome sequences and **d** protein-coding sequences relative to the other five representative genomes. **e** Genome and gene features of *P. cordatum* relative to the other five taxa, showing haploid genome size estimated based on sequence data, number of protein-coding genes, intron lengths, and separately for introns that contain introner elements (IE^+^), and those that lack these elements (IE^−^), known repeat types, and types of duplicated genes

We generated a de novo haploid, repeat-rich genome assembly from *P. cordatum* CCMP1329 (4.15 Gb, scaffold N50 length = 349.2 kb; Table 1 and Fig. 1a) [38]. Compared to five representative genomes of dinoflagellates [21, 39–42] from diverse ecological niches (Additional File 1: Supplementary Note [43–53]), *P. cordatum* has the highest G+C content in the genome sequences (mean 59.7%; Fig. 1c and Table 1) and in protein-coding genes (mean 65.9%; Fig. 1d and Table 2), compared to the moderate G+C content observed in the bloom-forming *A. gibbosum* [23] and the free-living *Polarella glacialis* [39] (Table 1). The larger genome of *P. cordatum* encodes more protein-coding genes with longer introns than do the other species (Fig. 1E, Table 2, and Additional File 2: Fig. S1). These introns are enriched in introner elements, i.e., introns that contain simple inverted repeats at both ends (Additional File 3: Table S1), suggesting a prevalence of these elements in the genomes of free-living dinoflagellates (Additional File 1: Supplementary Note, Additional File 2: Fig. S2, and Additional File 3: Table S2).

**Table 1:** Statistics of assembled genomes of *P. cordatum* and other dinoflagellates

Species	*Prorocentrum cordatum*	*Amphidinium gibbosum*^a^	*Polarella glacialis*	*Symbiodinium natans*	*Durusdinium trenchii*	*Cladocopium proliferum*	*Amoebophrya ceratii*
Isolate	CCMP1329	Not defined	CCMP1383	CCMP2548	CCMP2556	SCF055; formerly *C. goreaui* [53]	AT5.2
Reference	This study	Beedessee et al. [23]	Stephens et al. [39]	González-Pech et al. [21]	Dougan et al. [40]	Chen et al. [41]	John et al. [42]
Assembly size (Gbp)	4.15	7.55	2.99	0.76	1.71	1.17	0.09
Estimated genome size based on *k*-mers (Gbp)	4.75	6.30	1.48	0.74	1.05	1.30	0.12
Number of scaffolds	22,724	4,221,750	33,494	2,855	29,137	6,843	2,351
Genome scaffolds N50 (kbp)	349.2	150.4	170.3	610.5	774.3	353.9	84.0
Maximum scaffold length (Mbp)	3.56	1.36	2.17	3.40	4.57	7.38	0.54
Genome GC-content (%)	59.7	47.1	45.9	51.8	49.8	44.4	55.9
BUSCO proteins recovered (%) [genome mode; alveolata_odb10]	57.9	48.5	60.2	62.0	61.4	66.7	82.5

^a^Values for *Amphidinium gibbosum* (not displayed in Fig. 1) were derived directly from the published assembly version 1.0 [54]

**Table 2:** Statistics of predicted genes in *P. cordatum* and other dinoflagellates

Species		*Prorocentrum cordatum*	*Amphidinium gibbosum*^a^	*Polarella glacialis*	*Symbiodinium natans*	*Durusdinium trenchii*	*Cladocopium proliferum*	*Amoebophrya ceratii*
Isolate		CCMP1329	Not defined	CCMP1383	CCMP2548	CCMP2556	SCF055; formerly *C. goreaui* [53]	AT5.2
Reference		This study	Beedessee et al. [23]	Stephens et al. [39]	González-Pech et al. [21]	Dougan et al. [40]	Chen et al. [41]	John et al. [42]
Number of predicted genes		85,849	85,139	58,232	35,270	55,799	45,322	19,925
Recovery of BUSCO proteins (%) [protein mode; alveolata_odb10]		61.4	45.0	70.2	74.9	69.6	82.4	86.5
Genes with transcript support (%)		84.8	75.9	94.0	83.0	75.7	82.5	24.4
Average gene length (bp)		24,462	26,201	16,206	8780	15,334	15,745	2772
Average CDS length (bp)		2798	1193	1230	1660	1647	2018	1964
CDS GC-content (%)		65.9	54.9	57.8	58.2	55.7	54.2	60.8
Number of exons per gene		11.7	8.0	11.6	15.7	16.7	17.2	3.4
Average exon length (bp)		239.9	184.8	105.7	106	98.7	120.4	578.7
Genes with introns (%)		83.7	92.7	73.8	85.5	93.1	95.9	71.3
Number of introns per gene		9.8	7.0	10.6	14.7	15.7	16.2	2.4
Average intron length (bp)		4709	3732	1408	486	869	839	377
Splice donor motif (%)	GT	24.7	74.9	28.8	23.6	30.3	36.6	99.9
	GC	51.4	25.0	52.7	58	52.3	43.6	< 0.1
	GA	23.8	0.1	18.5	18.4	17.4	19.8	0
Splice acceptor with AGG motif (%)		79.7	93.2	96.9	97.1	96.5	96.1	56.5
Number of intergenic regions		48,574	47,727	35,271	33,042	47,452	39,720	17,856
Average length of intergenic regions (bp)		26,278	26,756	21,625	11,585	13,222	7388	1522

^a^Values for *Amphidinium gibbosum* (not displayed in Fig. 1) were derived directly from the published gene models version 1.0 [54]

**Fig. 2:**
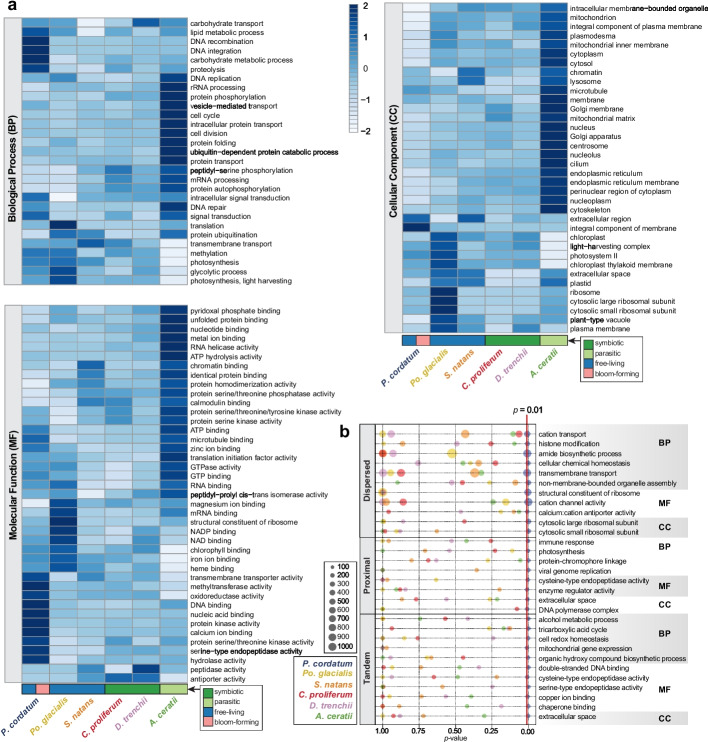
Gene functions encoded in the *P. cordatum* genome. **a** Gene functions encoded in the genome of *P. cordatum* and the other five representative taxa based on relative abundance of Gene Ontology (GO) terms per genome, shown for categories of Biological Process (BP), Molecular Function (MF), and Cellular Component (CC). The ecological niche for each taxon is shown at the bottom of the heatmaps. **b** GO terms that are significantly enriched (*p* < 0.01) among genes for each duplication type (i.e., dispersed, proximal and tandem) in *P. cordatum*, relative to *p*-values observed for the other taxa, and the associated number of genes for each GO term

We predicted 85,849 gene models in *P. cordatum* [38], 41,078 (47.8%) of which were annotated using a stringent approach (Additional File 3: Tables S3 and S4; see “Methods”); about half (52.2%) are assigned as “dark,” coding for functions yet to be discovered [47]. Based on the relative abundance of annotated Gene Ontology (GO) terms (Fig. 2a) in *P. cordatum* genes, we observed more-abundant functions related to metabolism, cell signaling, transmembrane transport, and stress response (Additional File 1: Supplementary Note and Additional File 2: Fig. S3). Most genes are unidirectionally encoded in *P. cordatum* genome, as similarly observed in genomes of other dinoflagellates (Additional File 2: Fig. S4), e.g., [39]. Interestingly, a substantial proportion (64.8%) of the 85,849 genes in *P. cordatum* are dispersed duplicates (Fig. 1e and Additional File 3: Table S5), suggesting that most duplication events occurred independently; alternatively, collinearity of duplicated blocks was disrupted by extensive rearrangements, due in part to the abundant transposable elements, as expected in dinoflagellate genomes [21, 55, 56]. We found significantly enriched (*p* ≤ 0.01) gene functions in the distinct types of gene duplicates (Fig. 2b), e.g., transmembrane transport and organelle assembly among the dispersed duplicates, compared to metabolic processes (e.g., tricarboxylic acid cycle [TCA]) and binding of biomolecules/ions among the tandem duplicates (Additional File 3: Table S6). These results demonstrate that distinct duplication modes have shaped the evolution of *P. cordatum* genes and their functions. Moreover, 47 genes were potentially acquired via horizontal transfer from uncultivated marine prokaryotes with functions related to structural conversion of amino acids and biosynthesis of metabolites (Additional File 1: Supplementary Note, Additional File 2: Fig. S5, and Additional File 3: Table S7).

### Integrated multi-omics analysis of heat stress responses specific to *P. cordatum*

**Fig. 3:**
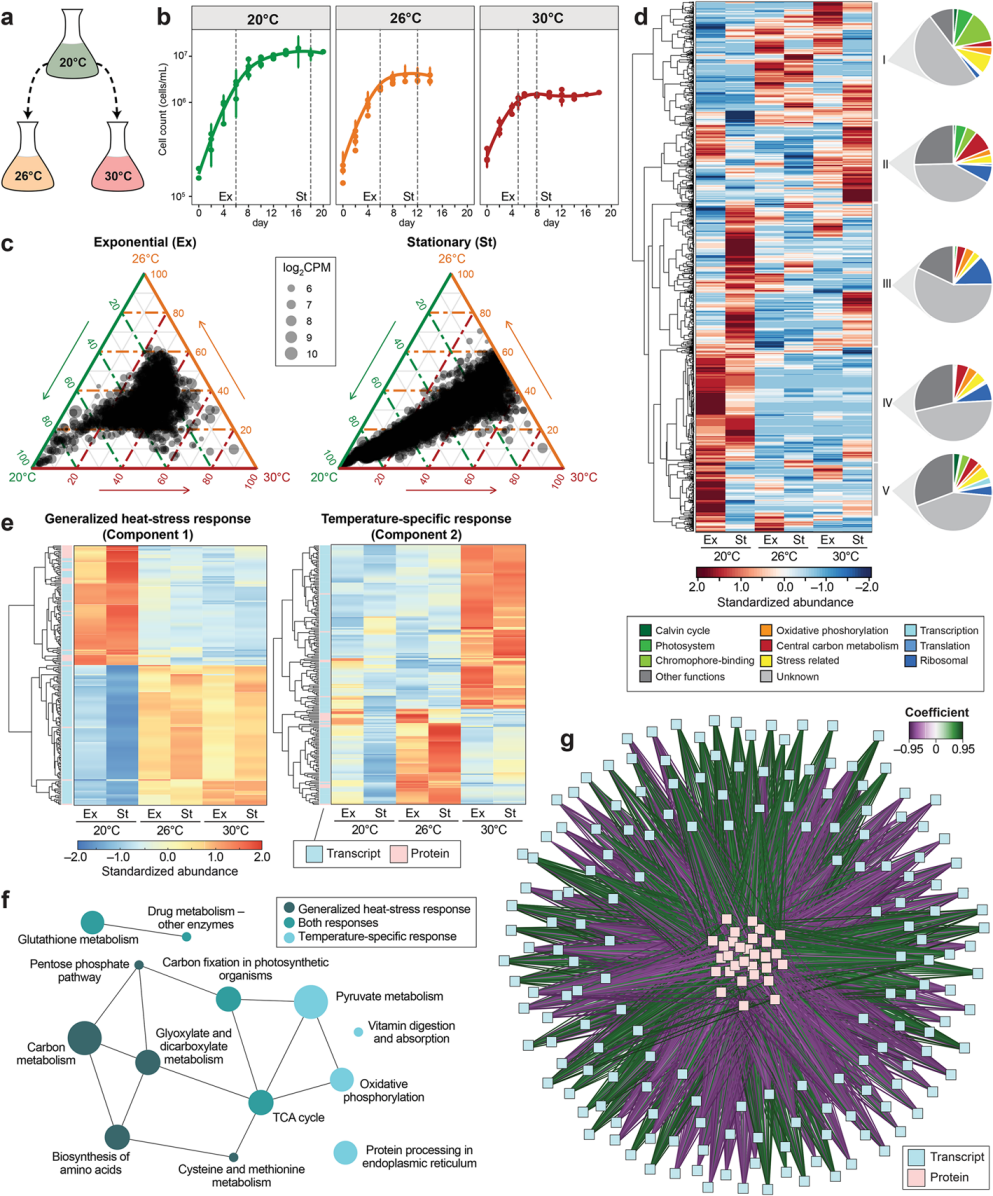
Integrated analysis of the transcriptome and proteome response of *P. cordatum* to heat stress. **a** Experimental design. **b** Growth of *P. cordatum* at 20, 26, and 30°C. Collection of cells for multi-omics analysis is indicated by dashed vertical lines (Ex: exponential, St: stationary phase). **c** Ternary plots of highly expressed gene models with mean log_2_(count per million) > 5 in response to temperature and growth phase (8593 transcripts in each plot). **d** Clustering of 2098 differentially abundant proteins in response to temperature and growth phase. Abundances of proteins were calculated from standardized peptide counts. **e** Heatmap of transcripts and proteins showing significant correlations for generalized heat stress response (component 1) and temperature-specific response (component 2). **f** Over-represented KEGG pathways in the networks of generalized and temperature-specific heat stress response. **g** DIABLO network of generalized heat stress response (component 1) revealing positive and negative correlations (coefficient ≥ 0.7) between transcripts and proteins

To investigate the heat stress response in *P. cordatum*, axenic cultures were grown in defined media at the optimal temperature (20°C) before they were exposed to either 26 or 30°C (Fig. 3a); 30°C is already observed in summers during which dinoflagellates commonly form blooms [57], thus is ecologically relevant in this context. We observed similar growth rates (0.33–0.47 day^−1^ and 0.47–0.68 doubling rate day^−1^) under all three conditions, but relative to the final cell density observed at 20°C, algal biomass was reduced to 62% and 41% at 26 and 30°C, respectively (Fig. 3b). Stable cell numbers over two weeks at stationary phase at both elevated temperatures indicate the tolerance of *P. cordatum* to heat stress. Transcriptome [38], proteome [58], and metabolome [58] data (Additional File 3: Tables S8, S9, S10 and S11) were generated from cells harvested independently at exponential (Ex) and stationary (St) growth phases in the three temperature conditions (Fig. 3b; see “Methods”).

Analysis of the transcriptome data (1.96 Gb, ~ 110 million reads per sample; Additional File 3: Table S8) from 18 samples (6 conditions × 3 replicates) using principal component analysis revealed a clear separation between 20°C and the higher temperatures in both Ex and St phases (Additional File 2: Fig. S6 and Additional File 3: Table S9), although gene expression changes are less pronounced in the Ex phase (Fig. 3c). Analysis of soluble and membrane protein fractions yielded 2098 proteins, of which 1032 were of unknown function; 244 are unique to *P. cordatum*. The 68 chromophore-binding (antennae) proteins of the photosystem comprised the largest group, accounting for 70.6% of detected peptides. We found 779 proteins with significantly changed abundance at 26 or 30°C compared to 20°C, with functions related to photosynthesis, energy generation, and central metabolism (Fig. 3d, Additional File 2: Fig. S7; all detected proteins shown in Additional File 3: Table S10). Metabolome analysis (see “Methods”) yielded 173 compounds, of which 73 could be identified (Additional File 2: Fig. S8 and Additional File 3: Table S11). Fifty-four metabolites displayed significantly changed abundances (*p* < 0.05) in response to growth phase and temperature, particularly those involved in central metabolism and amino acid biosynthesis (Additional File 3: Table S11).

To identify the molecular response in *P. cordatum* to heat stress, we integrated the transcriptome and proteome data using DIABLO [59] to identify shared multi-omics signatures of the Ex and St phases. This analysis revealed two types of heat stress response: a generalized response (component 1) with abundance changes common to both elevated temperatures (26 and 30°C), and a temperature-specific response (component 2) with abundance changes specific to 26°C or to 30°C (Fig. 3e); transcripts and proteins comprising these two components are shown in Additional File 3: Table S12. KEGG pathways for carbon metabolism such as the pentose phosphate pathway, glyoxylate and dicarboxylate metabolism, the biosynthesis of amino acids, and the metabolism of cysteine and methionine were enriched in the generalized response (component 1; Fig. 3f and Additional File 2: Fig. S9). In contrast, oxidative phosphorylation, protein processing in endoplasmic reticulum, vitamin digestion and absorption, and pyruvate metabolism pathways were enriched in the temperature-specific response (component 2; Additional File 2: Fig. S10). Our results also reveal both positive and negative correlations of expression between transcripts and proteins in component 1 (Fig. 3g) and component 2 (Additional File 2: Fig. S11). In component 1 for instance, gene expression of glutamine synthetase was positively correlated to the expression of chlorophyll *a*-*b* binding protein and the light harvesting complex I LH38 proteins, whereas it was negatively correlated to protein expression of pyruvate dehydrogenase, and the sulfate and formate transporters.

### Heat-induced multi-omics modulation of central metabolism in *P. cordatum*

**Fig. 4:**
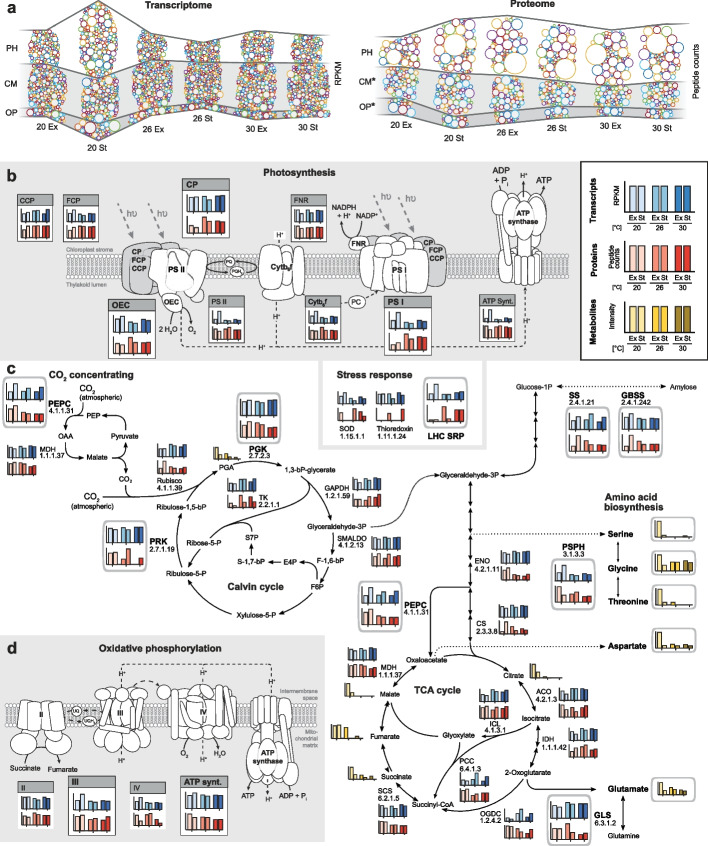
Heat stress response of central modules of energy and carbon metabolism in *P. cordatum*. **a** Temperature-dependent dynamics of sub-transcriptomes (left) and sub-proteomes (right) associated with photosynthesis (PH), central metabolism (CM), and oxidative phosphorylation (OP). Colored circles represent individual transcripts and proteins, respectively, with their areas proportional to the determined abundances. For the proteome, heights of the CM- and OP-bands (marked with an asterisk) were magnified tenfold to allow easier comparison with the PH-band. Expression of transcripts, proteins, and metabolites is shown for functions specific to **b** light reaction of photosynthesis, showing CCP, carotenoid/chlorophyll-binding protein; CP, chlorophyll-binding protein; FCP, fucoxanthin/chlorophyll-binding protein; FNR, ferredoxin:NADP oxidoreductase; OEC, oxygen evolving complex; and PS, photosystem; **c** central metabolism including CO_2_-concentrating Calvin cycle, central carbon metabolism, and selected biosynthesis of amino acids, showing PEPC, phosphoenolpyruvate carboxylase; GBSS, granule-bound starch synthase (NDP-glucose-starch glycosyltransferase); GLS, glutamine synthetase; PGK, phosphoglycerate kinase; PRK, phosphoribulokinase; PSPH, phosphoserine phosphatase; and SS, starch synthase; and **d** oxidative phosphorylation. A detailed scheme is presented in Additional File 2: Fig. S12

Given the lower biomass observed at elevated temperatures (Fig. 3b), we studied the recovery of biomolecules specific to three metabolic modules that drive growth and primary production in *P. cordatum*: photosynthesis, central metabolism, and oxidative phosphorylation. A global visualization of the relevant expressed transcripts (753) and proteins (278) revealed differential expression at elevated temperatures, with a marked increase in photosynthesis proteins but a decreased abundance of proteins related to central metabolism and oxidative phosphorylation (Fig. 4a). This result is supported by reduction in the accumulation of 38 metabolites associated with central metabolism at elevated temperatures. We also observed a similar pattern of elevated expression in transcripts and proteins coding for photosynthetic functions in the St phase relative to Ex phase at 20°C.

The detected components of the photosynthetic electron transport chain (PETC) showed varying abundance profiles (Fig. 4b). Whereas chlorophyll-binding proteins (CPs), light harvesting complex (LHC), stress-related proteins, and photosystem I (PS I) proteins increased at elevated temperature, subunits of the chloroplast ATP synthase did not change. Notably, PS II, and the associated oxygen evolving complex (OEC) and enzyme components of the response to reactive oxygen species (Additional File 3: Tables S13 and S14), appeared unchanged, but complex IV and mitochondrial ATP synthase of oxidative phosphorylation showed reduced abundance at higher temperature (Fig. 4d). Taken together, during heat stress *P. cordatum* faces energy deprivation arising from a less efficient PETC, which should directly impact protein synthesis and central metabolism. Accordingly, amino acid synthesis was reduced, demonstrated by decreased levels of phosphoserine phosphatase (PSPH) and glutamine synthetase (GLS), amino acids (e.g., serine and glutamate), and TCA-cycle intermediates (e.g., succinate) (Fig. 4c; detailed reconstruction of central metabolism in Additional File 2: Fig. S12). These results suggest lower levels of enzymes involved in concentrating CO_2_ (phosphoenolpyruvate carboxylase [PEPC]) and in ATP-consuming reactions of the Calvin cycle, e.g., phosphoribulokinase (PRK) and phosphoglycerate kinase (PGK) (Fig. 4c). The generally unchanged profile of CO_2_-fixing ribulose 1,5-bisphosphate carboxylase (RuBisCo; Calvin cycle) may be misleading in this context; because activity of form I RuBisCo is known to decrease at higher temperatures [60], we expect a similar trend for form II RuBisCo in dinoflagellates as well. Enzymes involved in the synthesis of amylose, e.g., starch synthase (SS) and granule-bound starch synthase (GBSS), remained stable (Fig. 4c). In contrast to the declining levels of TCA metabolites and amino acids, some carbohydrates and fatty acids increased at elevated temperature, suggesting the recycling metabolites and re-organization of cellular processes and structural elements (e.g., lipids) to protect cells from heat stress, or as a countermeasure against increased membrane fluidity [61]. These results demonstrate the severe impact of heat stress on essential metabolic processes that attenuated *P. cordatum* growth.

### Transcriptional dynamics under heat stress

**Fig. 5:**
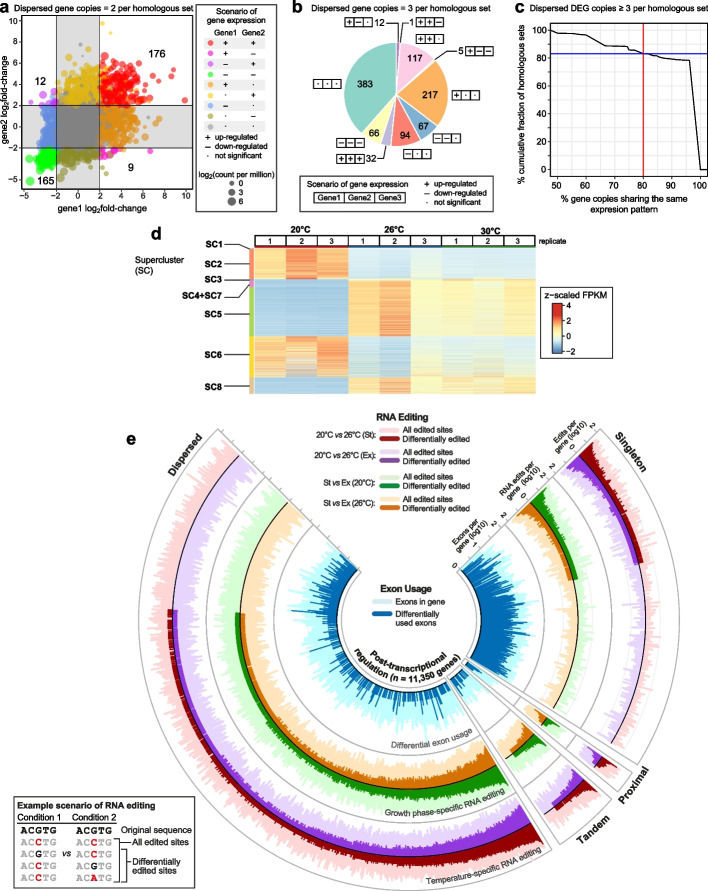
The transcriptome landscape under heat stress. The expression pattern of dispersed duplicated genes under treatments comparing 26°C against 20°C at St phase, shown for homologous sequence sets that contain **a** two and **b** three copies, in three possible outcomes: upregulated (+), downregulated (−), and not significant (·). **c** Proportion of dispersed DEGs that share similar expression pattern in homologous sets containing ≥ 3 of such copies. **d** Heatmap and clusters of gene expression pattern across triplicate samples at 20, 26, and 30°C in the St phase, showing eight superclusters (SCs). Centered log_2_(FPKM + 1) values are shown. **e** Post-transcriptional regulation in *P. cordatum*, shown for 4550 genes in distinct duplication modes (counter-clockwise): dispersed, tandem, proximal, and singleton. Features shown from the inner-most to the outer-most circle: differential exon usage (blue), differential editing of mRNA per gene in response to growth phase (green/brown), and differential mRNA editing per gene in response to temperature (red/purple). In each circle, a bar in a light shade indicates the number of corresponding features identified in a gene, a bar in a dark shade indicates the number of statistically significant features in a gene. The bottom-left legend shows the distinct scenarios for identifying sites for mRNA editing in all transcripts *versus* differentially edited sites

The transcriptome of *P. cordatum* showed extensive differentially expressed genes (DEGs) under heat stress: 2142 in the Ex phase, and 22,924 in the St phase (Additional File 3: Table S15), and we observed no strong evidence of batch-effect biases (Additional File 2: Fig. S6a). The most extensive difference (14,322 DEGs) was observed between 20 and 26°C in the St phase (i.e., a chronic heat stress response), of which 11,159 were also observed between 20 and 30°C (Additional File 2: Fig. S6b). Among 2368 homologous gene sets that contain two dispersed gene copies, 368 contain both copies as DEGs, of which 341 shared a similar expression profile (176 + 165 in Fig. 5a). Of the 994 sets containing three dispersed gene copies, 982 (98.8%) consist of two or more copies showing similar expression patterns (Fig. 5b). Moreover, among 949 sets containing ≥ 3 dispersed copies as DEGs, 789 (83.1%) contain 80% of such copies that were expressed similarly (Fig. 5c). The conserved expression patterns for this subset of dispersed duplicates hint toward gene dosage as a driver of the transcriptome in *P. cordatum* despite the lack of evidence for whole-genome duplication [40]; see Additional File 1: Supplementary Note and Additional File 2: Fig. S13 for more detail.

Based on the expression pattern across the three temperature conditions, DEGs were grouped into eight superclusters (SCs) independently at Ex (Additional File 2: Fig. S14a, Additional File 3: Table S16) and St phase (Fig. 5d, Additional File 2: Fig. S14b, Additional File 3: Table S17). Of the 22,924 DEGs in St phase, 11,647 (50.8%) showed an increase in expression from 20, 26 to 30°C (SC3–5, SC7 and SC8), 4853 (21.2%; SC1 and SC2) showed a gradual decrease, and 6424 (28.0%; SC6) showed little changes (Fig. 5d, Additional File 2: Fig. S14b). Enrichment analysis of GO terms revealed that at 26°C relative to 20°C in St phase, functions related to inositol oxygenase activity, cellulase activity, ATP-binding, and metal ion binding were upregulated (Additional File 2: Fig. S15a, Additional File 3: Table S17), whereas those related to ribosome, rRNA binding, translation, transmembrane transporter, photosystem II, and photosynthesis light reaction were downregulated (Additional File 2: Fig. S15b). At Ex phase, functions related to structural constituents of cytoskeleton and microtubule were downregulated at 26°C (Additional File 3: Table S18). These results are consistent with the observation of enriched metabolic pathways (Additional File 2: Fig. S15, Additional File 3: Tables S19 and S20), and GO terms among the SCs (Additional File 2: Fig. S14c). Interestingly, among 13 polyketide synthase I genes, likely involved in biosynthesis of most dinoflagellate toxins [62], five were upregulated and none were downregulated under heat stress (Additional File 3: Table S21). These results highlight the dynamic transcriptional response of *P. cordatum* to heat stress, which is greater than previously reported for any dinoflagellate; see Additional File 1: Supplementary Note for more detail.

### Post-transcriptional regulation via the complementary interplay of RNA editing and exon usage

The integration of genome and transcriptome data revealed two modes of post-transcriptional regulation in *P. cordatum* that may generate protein diversity (and functions): (a) alteration of a single base in mRNA (i.e., differential RNA editing), and (b) alternative splicing that leads to preferential usage of exons (i.e., differential exon usage: DEU); see “Methods.” We found evidence of post-transcriptional regulation in 11,350 genes (Fig. 5e): 9098 genes (involving 45,180 sites) with differential RNA editing, 1820 genes (involving 6243 exons) with DEU (Additional File 3: Table S22), and 432 genes (involving 2367 sites and 1177 exons) with both modes of regulation. The number of mRNA edits was similar under all conditions, but the type of edit and overlap between edited sites was condition-specific (Fig. 5e, Additional File 4: Data S1).

Interestingly, the relatively few (432) genes with both RNA editing and DEU in *P. cordatum* suggests that post-transcriptional regulation of a gene tends to involve only one of these two modes: the editing of RNA was identified predominantly in genes for which no DEU was identified, and vice versa (Fig. 5e); singleton genes showed a greater extent of DEU in contrast to the dispersed duplicates that exhibited a greater extent of RNA editing. The 432 genes displaying both modes of regulation encode key photosynthetic and stress-related functions, such as chloroplastic ATP synthase subunits (β and ε subunits), ferredoxin, photosystem I reaction center subunit XI, and heat shock 70 kDa protein. The complementary role of these two pathways for post-transcriptional regulation in central pathways (e.g., photosynthesis) may represent a key mechanism for generating functional diversity of the proteome in *P. cordatum* (and potentially more broadly in dinoflagellates) to enable quick acclimation to environmental changes.

### Regulation of multi-protein-coding gene variants as a concerted heat stress response

To assess the impact of RNA editing and exon usage on the heat stress response, we focused on genes that undergo both of these processes. An example is the gene coding for heat shock protein 70 kDa (HSP70), a ubiquitous chaperone associated with stress responses. Of the 16 *P. cordatum* gene models that putatively code for HSP70 of varying lengths (574–2575 amino acids), one (s12246_g74608) encodes multiple full-length copies of the functional protein. The protein-coding sequence is organized in multiple sub-regions, in which each sub-region encodes the same full-length protein; we follow Shi et al. [63] and define such a sub-region within a gene model as a coding unit (CU), and the entire sequence as a multi-CU gene model. These CUs are separated by spacer sequences that are collectively transcribed as a single transcript that was then translated as a single protein sequence, after which the spacer peptides were removed (Additional File 2: Fig. S16c). This is slightly different from the usual polycistronic transcription in dinoflagellates (Additional File 2: Fig. S16b), whereby multiple (different) genes are co-transcribed as a primary transcript that is processed subsequently, each gene via *trans*-splicing at the 5′-end and polyadenylation at the 3′-end [31], into individual mature transcripts. HSP70 genes in other unicellular eukaryotes such as *Leishmania* and trypanosomes are also polycistronically transcribed, with the 3′-UTR and regions downstream of the protein important for translational regulation [64–66]. Polycistronic transcripts in dinoflagellates are thought to be converted into monocistronic sequences via *trans*-splicing of a conserved 22-nucleotide dinoflagellate spliced leader (dinoSL) sequence [31]. We found 4.95% of *P. cordatum* transcripts to contain dinoSL (Additional File 3: Table S23) involving 17,214 (20.1% of 85,849) gene models, including those encoding HSP70, lending further support to this idea (Additional File 1: Supplementary Note). However, due to the technical limitations of identifying dinoSL in transcriptome data generated from RNA-Seq in this study (for which the assembled transcripts are likely fragmented), we took caution not to over-interpret these results.

**Fig. 6:**
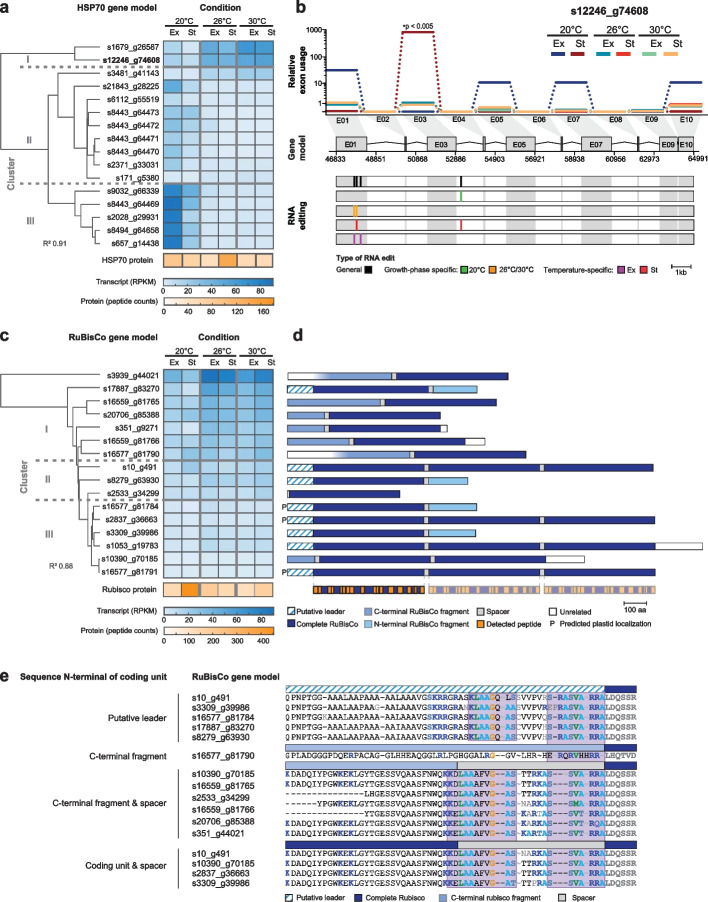
Expression and structure of multi-protein-coding genes in *P. cordatum*. The example of HSP70 is shown for **a** clustering of distinct gene models based on gene expression pattern in a heatmap across growth conditions, with cumulative abundance of transcripts and proteins indicated at the bottom, and **b** the relative exon usage, CDS structure, and mRNA editing of gene model s12246_g74608 that harbors multiple CUs. Exon (E) is connected by introns (kinked line); each of E01, E03, E05, and E07 encodes a complete HSP70, E09, and E10 each encodes N-/C-terminal fragments, whereas the other exons encode spacers. The example of RuBisCo is shown for **c** clustering of gene models based on gene expression pattern in a heatmap across growth conditions, with cumulative abundance of transcripts and proteins indicated at the bottom, and **d** protein structures corresponding to complete CUs, N- and C-terminal fragments, spacer, and putative leader sequences, with detected peptides indicated at the bottom. **e** Aligned upstream regions of 16 complete RuBisCo proteins of *P. cordatum*, including leader sequence and representative spacer sequences where available. Basic residues are marked in dark blue and conserved regions are highlighted in a purple background

In *P. cordatum*, the multi-CU gene model is the main contributor to the heat stress response of HSP70 along with one single-CU gene model in cluster 1 (Fig. 6a). Exon 1 (CU-1) of the multi-CU gene displayed a unique pattern of RNA editing (Fig. 6b), with contrasting RNA edits specific to growth phase and temperature, including multiple sites upstream of the transcriptional start site (Additional File 4: Data S1). In this gene, exons 1, 3, 5, and 7 constitute four CUs, with exons 9 and 10 encoding a partial CU (Fig. 6b, Additional File 2: Fig. S17). The usage of exon 3 (CU-2) was biased toward a higher abundance at 20°C of St phase, in combination with multiple specific intronic RNA-edited sites downstream of this exon. Interestingly, the HSP70 encoded by this exon lacks a disordered C-terminus region involved in protein–protein interactions in many species.

To assess expression heterogeneity in response to heat stress among paralogs occurring as single-CU and multi-CU gene models, we selected RuBisCo for its central physiological function. Of the 32 *P. cordatum* gene models that putatively encode RuBisCo (of sizes 163 to 5197 amino acids; Additional File 2: Fig. S18), one-half encode fragments of the protein, whereas the remainder are organized in single or multiple CUs (Fig. 6d). Distinct from polycistronic transcripts, here, each CU in a gene model does not have a termination codon for translation (Additional File 2: Fig. S16c). Earlier studies of two *Prorocentrum* species [63, 67] revealed a single transcript encoding three or four consecutive CUs, and the unprocessed protein product is thought to be transported into the plastid where it is split into separate, active proteins [63]. Here, we found gene models that encode three CUs (4), two CUs (1), and a single CU (11; Fig. 6d); dinoSL-containing transcripts were identified for some of these, e.g., in the three-CU-encoding s10_g491 (Additional File 3: Table S23).

Transport of RuBisCo to the plastid is facilitated by transit peptides including a leader sequence encoded upstream of the gene; these sequences are highly diverged among photoautotrophs [68, 69]. In dinoflagellates, an elevated serine/threonine and alanine content, and a potential motif of FVAP close to the N-terminus was predicted [70, 71]. We found, in the upstream region of eight gene models (Fig. 6d), the predicted leader sequence from the closely related *Prorocentrum shikokuense* [63]. We identified two conserved sequence motifs in the leader sequence, the N-terminal region of proteins that lacked a leader sequence, and the spacer region between two CUs (Fig. 6e). The two sequence motifs are moderately hydrophobic and rich in basic amino acid residues (i.e., arginine or lysine) as expected for plastid transit peptides [69]. We also observed a high content of serine and alanine, and an FVGA motif in the spacer regions (upstream of the N-terminus; Fig. 6d). The conserved sequence regions indicate that proteins coded by these single- and multi-CU genes in *P. cordatum* can be transported into the plastid. Highly similar sequence motifs present in *P. shikokuense* and other dinoflagellates support this hypothesis, and these motifs may be taxon-specific, e.g., FVGA is conserved in *Prorocentrum*, but not in the analyzed RuBisCo proteins of *Heterocapsa*, *Symbiodinium*, and *Ligulodinium* taxa (Additional File 2: Fig. S19).

Associating expressed peptides to distinct CUs is not straightforward due to the high pairwise protein sequence identity (> 95%). The cumulative abundance of proteins is highest at 20°C in St phase, whereas transcript expression increased with elevated temperature, for which the clustered expression profile follows the number of CUs in the transcript (Fig. 6c); this pattern was not observed in HSP70. In RuBisCo, multi-CU transcripts had a consistently low expression level, whereas single-CU derived transcripts were more strongly differentially expressed: i.e., upregulated under heat stress (Fig. 6c, Additional File 2: Fig. S20). Nine of the 16 complete RuBisCo gene models are in single exons, and no differential exon usage was identified in the multi-CU gene models. The modulation of expression responses in multi- *versus* single-CU transcripts reflects a regulatory mechanism in addition to alternative exon usage or RNA editing.

## Discussion

The high gene density, long introns [72, 73], and extensive genetic duplication in *P. cordatum* likely reflect genomic hallmarks of bloom-forming dinoflagellates, consistent with data from *A. gibbosum* [23]. Habitats of *P. cordatum* are known to have rapidly fluctuating temperatures, such as during diurnal vertical migration or in spring blooms and red tides. Diurnal vertical migration of plankton in the ocean is known to cover many hundreds of meters, encompassing large gradients of temperature, oxygen, and nitrate [74]. Although distances of vertical migration of dinoflagellates are little known, earlier studies have demonstrated migration of *P. minimum* (= *P. cordatum*) across an artificially established halocline [75] and *Prorocentrum micans* across temperature gradients of 7°C [76]. Compared to dinoflagellates of family Symbiodiniaceae well-known as the coral symbionts found in warm, sub-tropical coral reef ecosystems, *P. cordatum* is found in cooler, temperate waters. Therefore, the elevated G+C content of the *P. cordatum* genome does not appear to be a signature of heat resistance, and instead may be favored by selection to ensure high fidelity of transcription (i.e., G-C base pairs are more thermally stable) in open oceans. The long introns and presence of introner elements in the genome points to active transposition, i.e., non-autonomous DNA transposons, in contributing to the extensive rearrangement and duplication of genes [45, 46, 55], and to the large genome size with many functionally redundant but slightly different transcript isoforms that provide rich adaptive resources in frequent changing environments in the aquatic realm.

Our data [38, 58] were generated from axenic cultures, thus the results reflect strict photoautotrophy, without the influence of cohabiting microbes, a host organism, and/or potential mixotrophy. *P. cordatum* is able to grow as a photoautotroph under heat stress [77], supported by our observation of stable growth at 30°C. We report a dynamic transcriptional response to heat stress, with a more profound reprogramming during stationary growth phase compared to exponential phase. Our multiple lines of omics data demonstrate a strong coordinated response, particularly under chronic heat stress, that reduces energy production and consumption via the reduction of photosynthesis, carbon fixation, and amino acid biosynthesis, resulting in a suppressed central metabolism and protein synthesis. These observations lend support to the results of earlier transcriptome studies of pelagic dinoflagellates [78, 79].

We present the first evidence of a complementary mechanism of post-transcriptional regulation in a pelagic dinoflagellate, involving RNA editing and exon usage for regulating molecular responses to elevated temperature. This mechanism likely provides the transcriptional flexibility that is essential for generating transcript and protein variants, and maximizes the functional diversity of gene products in dinoflagellates. In the absence of RNA editing and alternative exon usage, e.g., in single-exon genes that are common in dinoflagellates [39], a third system we describe is through the adjustment of the number of coding units. All these mechanisms are set against the backdrop of polycistronic transcription followed by *trans*-splicing [31, 52, 80].

## Conclusions

HABs are occurring at an increasing frequency due in part to global climate change [5]. Our results provide an integrated multi-omics perspective on the molecular responses to heat stress in a pelagic HAB-forming dinoflagellate. These results are set against the backdrop of a large, complex genome structure and multi-level transcriptional regulation. The multi-omics resources generated from this study provide a foundational reference for understanding the molecular regulatory and evolutionary processes of a HAB dinoflagellate species [81] in response to environmental stress. The dynamics and interplay of molecular regulatory mechanisms may explain in part the complex and large genome in the context of vast diversification and evolutionary success of dinoflagellates over 696–1520 million years [82].

## Methods

### Strain and culture conditions

The axenic culture of *Prorocentrum cordatum* strain CCMP1329 used in this work was obtained from the Provasoli-Guillard National Center for Marine Algae and Microbiota (NCMA). *P. cordatum* CCMP1329 was cultivated in L1 minus Si medium, which is a modified L1 medium [83] in which synthetic ocean water was used instead of natural seawater, and Na_2_SiO_3_·9H_2_O omitted. Cultures were routinely maintained by transferring 15-day-old culture (10 mL) to fresh medium (90 mL) in 300-mL Erlenmeyer flasks. The flasks were kept in a climate chamber (RUMED type 3501; Rubarth Apparate GmbH, Laatzen, Germany) at 20°C in a 12:12 h light:dark cycle, with light intensity ~ 40 µmol photons m^−2^ s^−1^ without agitation. Absence of contaminating bacteria was routinely checked by plating aliquots on LB and Difco™ marine agar 2216 (MB) plates. The number of cells mL^−1^ after transfer fluctuated between 2.0 × 10^4^ and 3.0 × 10^4^.

### Cell counting

Growth of *P. cordatum* was determined by cell counting using a BD LSR-Fortessa flow cytometer (BD Biosciences, San Jose, CA, USA). *P. cordatum* was identified according to its chlorophyll autofluorescence. Chlorophyll was excited with the 488-nm excitation laser and emission was detected at 695 nm. Samples (1 mL) were taken from three biological replicates during the light period and fixed with 25% glutaraldehyde (80 µL; final concentration 2% v/v) for 15 min at room temperature (RT). Samples were snap frozen in liquid nitrogen and stored at −70°C until they were analyzed. Each sample was analyzed in triplicates for 1 min.

Four independent growth curves per temperature were recorded. For each growth curve, counts were averaged from three biological replicates (each with three technical replicates). Counts were plotted against time and a generalized additive model (GAM) was fitted. The specific growth rate in the exponential growth phase (μ_exp_ per day) and the doubling rate per day (*k*) was calculated [84].

### Extraction of genomic DNA

DNA of *P. cordatum* cells extracted with commercially available plant kits or by applying the common CTAB protocol was either too fragmented or contained too many contaminating compounds to be suitable for PacBio sequencing. Therefore, we performed an initial ultrasound treatment to break the cells and separate the nuclei from the debris, based on work aimed at isolating nuclei for electron microscopy [85]. This treatment was not performed for extracting genomic DNA for Illumina NovaSeq sequencing.

*P. cordatum* culture (100 mL) was transferred to two 50-mL Falcon tubes and centrifuged (685* g*, 5 min, RT) using a Heraeus Multifuge™ X1R (Thermo Scientific, Schwerte, Germany). The supernatant was discarded, the pellets were dissolved in artificial seawater (15 mL) using an inoculation loop, and centrifuged (685* g*, 5 min, RT). The supernatant was discarded, the pellets were dissolved in 30% ethanol (15 mL), and centrifuged (685* g*, 5 min, RT); this step was repeated before the final pellet for each tube was dissolved in 30% ethanol (3 mL). For each tube, ultrasound using a Bandelin Probe Sonicator (Bandelin, Berlin, Germany) was applied (1 min, 5 cycles, amplitude 40%) followed by centrifugation (171* g*, 3 min, RT), and the resulting pellet was suspended in 0.85% NaCl (1.5 mL) and centrifuged (10,000* g*, 1 min, 4°C) using Heraeus Primo R Centrifuge (Thermo Scientific, Schwerte, Germany). Pellets from the two tubes were dissolved in high salt buffer (1 mL) in a 2-mL Eppendorf tube using inoculation loops. Proteinase K (8 µL) was added, and the mixture was incubated at 56°C (1 h), with the tubes inverted gently every 15 min. After cooling on ice (5 min), RNase A (15 µL) was added, and the mixture was incubated at 37°C (30 min, no shaking). The sample was centrifuged (10,000* g*, 5 min, 4°C) and the supernatant transferred to a new tube. NaCl (5 M, 200 µL) were added, followed by thorough mixing by inverting the tube. Solution of CTAB/NaCl (200 µL) was then added, mixed well by inverting the tubes, and the mixture was incubated at 65°C (10 min). Chloroform extraction was performed using chloroform:isoamyl alcohol (24:1 v/v, 1 mL), and repeated three times until no interphase was visible. To the collected aqueous phases, an equal volume of isopropanol (pre-chilled at −20°C) was added, and the mixture was incubated overnight at −20°C. DNA was centrifuged (10,000* g*, 10 min, 4°C) and the pellet was washed three times with 70% ethanol (pre-chilled at 4°C) with centrifugation (10,000* g*, 10 min, 4°C). Following air drying under the clean bench, the DNA pellet was dissolved in the elution buffer (200 µL).

The DNA samples were sent to Helmholtz-Center for Infection Research (Braunschweig, Germany) for Illumina NovaSeq 6000 (pair-end 2 × 150 bp) sequencing, and to the German Collection of Microorganisms and Cell Cultures (DSMZ, Braunschweig, Germany) PacBio Sequel and Sequel II sequencing.

### RNA isolation and sequencing

For RNA extraction, the sample was thawed at RT and transferred to a cryotube filled with 0.3 g acid-washed glass beads (100 µm). The cells were homogenized using the FastPrep-24 instrument (MP Biomedicals, Irvine CA, USA) at 6.0 m/s for 3 min (3 × 1 min, and 1 min on ice). Samples were centrifuged (12,000* g*, 10 min, 4°C; Heraeus Primo R Centrifuge), and the supernatants were transferred to fresh tubes and incubated at RT (5 min). Next, 1-bromo-3-chlorophenol (100 μL; Sigma-Aldrich, Taufkirchen, Germany) was added, and samples were shaken vigorously for 15 s and incubated at RT (10 min). Samples were centrifuged (12,000* g*, 10 min, 4°C), the aqueous phase was transferred to a new tube, to which isopropanol (0.5 mL) was added, mixed, and incubated at RT (10 min). The sample was then centrifuged (12,000* g*, 10 min, 4°C), after which the supernatant was removed. The RNA pellet was washed with 75% ethanol (1 mL) with centrifugation (7500* g*, 5 min, 4°C); this step was repeated. The final pellet was air-dried for 5 min, before being resuspended in RNase-free water (50 μL), and incubated at 55°C (10 min), prior to storage at −80°C.

Removal of genomic DNA was verified via PCR using total RNA as the template. The concentration of the RNA was quantified using a NanoDrop spectrophotometer (Peqlab, Erlangen, Germany) and the RNA integrity was assessed using a Bioanalyzer 2100 (Agilent, Santa Clara, USA). The average RNA concentration in the 18 samples was 297.4 ± 109.5 ng µL^−1^ and the average RIN value was 5.5 ± 0.78.

RNA sequencing was performed at the HZI Braunschweig with 300 cycles on a NovaSeq 6000 using pair-end 150 bp chemistry with the library kit NEBNext Ultra II directional RNA. Ribosomal RNA was depleted prior to sequencing using polyA beads.

### De novo genome assembly

Genome data from *P. cordatum* were generated using Illumina NovaSeq 6000 and PacBio sequencing technologies, with a total data yield of 843.4 Gb (Additional File 3: Table S24). Combining these sequence reads, a hybrid genome assembly was generated using MaSuRCA v4.0.3 [86], independently with CABOG (option *FLYE_ASSEMBLY* = *0*) and FLYE (option *FLYE_ASSEMBLY* = *1*) as the assembly tool in the final step. Both assemblies are near identical, in which 99.94% of the scaffolds in each assembly share 99.04% identity on average. They exhibit the same level of data completeness (56.7%) based on recovery of BUSCO single-copy orthologs in alveolata_odb10 dataset (which is known to be poor in dinoflagellate data). Between these two preliminary assemblies, MaSuRCA-CABOG is more contiguous (N50 length of scaffolds = 194.50 kb) and yields better recovery of the rRNA region (Additional File 3: Table S25); this assembly was used in the subsequent refinement steps.

To refine the assembled genome, we first incorporated RNA-Seq data to further scaffold the MaSuRCA-CABOG assembly using L_RNA_scaffolder [87]. Briefly, we mapped the de novo assembled transcriptome (for transcripts ≥ 500 bp) onto the assembled genome sequences using pblat v2.5 [88]. The mapping results in psl format were used as input for L_RNA_scaffolder. This approach yielded a more contiguous genome assembly (N50 length of scaffolds = 346.97 kb) with a better recovery of BUSCO genes (57.9%; Additional File 3: Table S25).

Next, using BlobTools v1.1.1 [89], we assessed the assembled genome for potential outlier sequences based on sequence coverage, G+C content, and shared sequence similarity to known sequences in NCBI nt database (release 243; 15 April 2021). Genome scaffolds for which the sequencing coverage or G+C content is external to the range of median ± 1.5 × interquartile range are considered as potential outliers. Scaffolds that have bacterial, archaeal, or viral sequences as the top hits plus extreme sequencing coverage or extreme G+C content are considered sequences that are putatively external to the nuclear genome of *Prorocentrum cordatum*. In this analysis, majority (23,366; 98.2%) of the 24,295 genome scaffolds (implicating 3.89 Gb) do not have hits in the BLAST searches (20,914 scaffolds; 2.95Mbp) or have top hits in an undefined eukaryote sequence (2452 scaffolds; 0.95Mbp); this observation is expected given the lack of dinoflagellate data in the existing databases. Using this approach, we identified 1571 outlier sequences and removed them from the genome assembly. Most outlier sequences do not have shared similarity to bacterial sequences in the database. This is expected given the algal cultures from which the genomic DNA was extracted were axenic. The final genome assembly [38] has a total size of 4.15 Gb (N50 length of scaffolds = 349.2 kb; Additional File 3: Table S25).

### Transcriptome assembly

RNA-Seq reads from six conditions (20_ex, 20_st, 26_ex, 26_st, 30_ex and 30_st) were processed using fastp v0.21.0 [90] using parameter *–g* to remove adapter sequences and poly-G artifacts known in NovaSeq 6000 data. Transcriptomes were first assembled in “de novo” mode using Trinity v2.12.0 [91] independently for each condition. All de novo assembled transcripts were combined as a single assembly, from which redundant sequences were identified and removed using CD-HIT v4.8.1 [92] (98% identity; 0.9 length-difference cutoff), yielding the final representative reference transcriptome.

To generate the genome-guided transcriptome, processed RNA-Seq reads from each condition were first mapped onto the final genome assembly (above) using HISAT2 v2.2.1 [93]. The mapping result (i.e., describing the alignments between RNA-Seq reads and genome scaffolds) was used as input for Trinity v2.12.0 in “genome-guided” mode. Using the same strategy above, individual genome-guided assemblies were combined, and redundant sequences removed, yielding the final representative genome-guided transcriptome.

### Ab initio prediction of protein-coding genes

To predict protein-coding genes, we follow the customized gene prediction workflow tailored for dinoflagellate genomes following Chen et al. [94]. The description of this workflow is available at [95]. Briefly, this approach integrates evidence of transcript and protein sequences to guide predictions using multiple gene programs, after which the results were integrated to yield the final gene models [38].

First, we identified novel repetitive elements in the genome assembly using RepeatModeler v2.0.1 [96], combined these elements with existing repeat families in RepeatMasker database release 2018/10/26 as a reference, to predict and mask all repetitive sequence regions from the genome sequences using RepeatMasker v4.1.0 [97]; this yields the repeat-masked genome assembly.

To predict protein-coding genes, we first mapped the representative de novo and genome-guided transcriptome assemblies to the genome assembly using Minimap2 v2.18 [98], for which the code was modified to recognize G-C and G-A donor splice sites. The mapping information was then used to predict transcript-based genes using PASA v2.3.3 [99] for which the code was modified to account for non-canonical splice sites. The proteins coded by the transcript-based genes were searched (BLASTP, *E* ≤ 10^−20^, > 80% query cover) against a customized protein database combining RefSeq (release 98) proteins and predicted proteins from available Symbiodiniaceae genomes (Additional File 3: Table S26). The gene models were checked for transposable elements using HHblits v3.3.0 [100] and TransposonPSI [101], searching against the JAMg transposon database [102]; those genes containing transposable elements were removed from subsequent steps. Redundant sequences were removed based on similarity using CD-HIT v4.8.1 [92] (*-c 0.75 -n 5*). Among the remaining transcript-based gene sequences, we identified high-quality “golden genes” using the script *Prepare_golden_genes_for_predictors.pl* from the JAMg pipeline [102], altered to recognize alternative splice sites. These “golden genes” represent high-quality training set for ab initio gene predictors. We used them as the training set for SNAP [103] and AUGUSTUS v3.3.1 [104] for gene prediction from the repeat-masked genome assembly; the codes for AUGUSTUS was also modified to recognize alternative splice sites [105].

The repeat-masked genome was also used as the input for GeneMark-ES [106]. We also predicted genes using MAKER v2.31.10 [107], in which the code was modified to recognize GA donor splice sites. Protein-coding genes were predicted using MAKER (*protein2genome* mode) based on protein sequences from the Swiss-Prot database (retrieved 02 March 2020) and predicted protein sequences from other Symbiodiniaceae genomes. Finally, gene predictions from the five methods including the ab initio predictors (GeneMark-ES, AUGUSTUS, SNAP), MAKER protein-based predictions, and PASA transcript-based predictions were integrated using EvidenceModeler v1.1.1 [108] to yield the high-confident gene models; the weighting is PASA 10, MAKER protein 8, AUGUSTUS 6, SNAP 2, and GeneMark-ES 2. These gene models were subjected to three rounds of polishing during which the gene models were corrected based on transcriptome re-mapping using the annotation update utility in PASA [99].

Introner elements were identified in the intronic regions using the program Pattern locator [109]. The patterns we searched for were inverted repeats of 8–20 nucleotides and direct repeats of 3–5 nucleotides within 30 bases of each end of the introns as described in Farhat et al. [43].

### Functional annotation of protein-coding genes

Annotation of gene function for *P. cordatum* and other representative dinoflagellate genomes was conducted based on sequence similarity to known proteins in the UniProt database (release 2021_03). Predicted protein sequences from the gene models were first searched against the manually curated protein sequences of Swiss-Prot (UniProt release 2021_03) using BLASTp v2.3.0 + (*E* ≤ 10^−5^; subject-sequence cover ≥ 70%). Sequences that have no Swiss-Prot hits were then searched against TrEMBL (UniProt release 2021_03) using the same parameters. For predicted proteins of *P. cordatum*, we further assessed functions based on sequence-similarity search against known protein sequences in EnzymeDetector, InterProScan, eggNOG, and Kofam.

### Prediction of transit peptides

For each predicted protein, transit peptides were first predicted independently using TargetP v2.0 (*-org pl*), SignalP v6 (*–organism eukarya –mode fast*), WoLF PSORT (*plant* mode), Predotar, and ChloroP v1.1. Subcellular localization is determined based on the consensus from these predictions confirmed in three or more programs.

### Analysis of homologous proteins

To identify homologous proteins of the predicted *P. cordatum* proteins, we compiled a comprehensive protein sequence database (1,554,705 sequences from 31 dinoflagellate taxa; Additional File 3: Table S27) using available genome or transcriptome data. All data from the MMETSP database [110] were downloaded from [111]. For species where there were multiple datasets for the same isolate, the protein sequences were clustered at 90% sequence identity using CD-HIT v4.8.1 [92] to reduce redundancy. Using these 31 sets of protein sequences, homologous sets were then identified based on clustering of protein sequences based on sequence identity using OrthoFinder v2.3.8 [112] at default settings.

### Identification of mRNA editing sites

Putative mRNA editing events were identified from single-nucleotide variations observed in genome sequence reads *versus* RNA-Seq reads. An observed nucleotide variation in the RNA-Seq reads but not in genome sequence reads is considered a potential mRNA edited site. Briefly, 25% of all genome sequence reads (randomly sampled) were mapped to the final genome assembly using bwa-mem v0.7.17-r1188 [113]. Trimmed RNA reads from each sample (6 conditions × 3 replicates) were mapped to the genome assembly separately with HISAT2 v2.2.1 [93] using default parameters (–no-discordant) and a HGFM index that was built using known exons and splice sites from the predicted gene models. PCR duplicates were marked by *MarkDuplicates* implemented in Picard v2.23.8 [114]. For each condition, mapping of RNA-Seq reads was compared with the mapped genome sequence reads using JACUSA v2 (*call-2 -F 1024 -P2 RF-FIRSTSTRAND -s -a D,Y,H:condition* = *1*) [115]. We follow the authors’ recommendation to assess the statistical significance of an mRNA edited site. A site is considered statistically significant if it meets all the requirements: (a) a score > 1.15; (b) coverage of genome reads > 10; (c) coverage of RNA reads from each condition > 5; (d) number of putative editing type is < 2; (e) the editing site is present in all three replicates.

### Analysis of horizontal gene transfer

To identify putative horizontal gene transfer (HGT), *P. cordatum* proteins were searched (BLASTP, *E* ≤ 10^−5^) against a customized protein sequence database that consist of 2,773,521 proteins from 82 other eukaryotes (Additional File 3: Table S28) and 688,212 proteins from 543 single-cell assembled genomes (SAGs) of prokaryotes [49] (Additional File 3: Table S29). Excluding hits to other *Prorocentrum* proteins, *P. cordatum* proteins that have a bacterial top hit are considered results of HGT involving *P. cordatum* and bacteria. To support the inferences of putative HGT, we employed OrthoFinder v2.5.4 [112] to infer homologous protein sets from all the involved proteins (i.e., *P. cordatum* proteins, proteins from other eukaryotes and SAGs). Homologous protein sequence sets that contain *P. cordatum* proteins implicated in HGT were multiply aligned using MAFFT v7.453 [116] at *–maxiterate 1000*. Trimmed with trimAl v1.4.1 [117] using parameter *-automated1*, the alignments were used to infer phylogenetic trees using IQ-TREE2 [118] at *-B 2000 -T AUTO*.

### Integrated mixOmics analysis

We conducted a multi-omics analysis in *P. cordatum* using the proteomic and transcriptomic data to identify a systems level heat stress response across the growth phases using the *mixOmics* package [119] in R. Transcriptome FPKM values were first log_2_ transformed prior to quality filtering with normalized proteomic data, requiring features be present across 75% of samples. Selected features represented the top 25 and 50% most variable transcripts (15,097) and proteins (259), respectively, that were present across 75% of the samples. These features were then input to mixOmics for Data Integration Analysis for Biomarker discovery using a Latent cOmponents (DIABLO) [59].

We conducted performance testing of the initial model to identify the number of latent components that contained a multi-omics signature using Mfold validation with 5 folds and 50 repeats. This suggested two latent components as the best fits for the model. We then performed final model tuning using the max distance to select diagnostic features for both component 1 (RNA-Seq: 170, proteins: 30) and component 2 (RNA-Seq: 190, proteins: 40). Ordination of all features indicate the separation of the three temperature levels for both transcriptome and proteome features. The transcriptome and proteome features selected for component 1 discriminate a common heat stress response at both 26 and 30°C (Additional File 2: Fig. S9, Additional File 3: Table S12) and for component 2, a heat stress response specific to either 26 or 30°C (Additional File 2: Fig. S10, Additional File 3: Table S12). The variates for the pathways from both components were then input to NetworkAnalyst [120] for KEGG pathway over-representation analysis to identify functional categories that were enriched in each network.

A relevance association network was created for each component using the *network* function within *mixOmics*, where values represent a robust approximation of the Pearson correlation. A heatmap displaying the features from each component was created using the *pheatmap* package in R with features clustered according to their Euclidean distances and scaled within rows. This revealed two main clusters within each component that were extracted using the R package *dendextend* according to the corresponding dendrogram. A relevance association network was then created for each subcluster as previously done for the two components.

### Experimental design of multi-omics analysis

Cultures of *P. cordatum* CCMP1329 were maintained as described above. After 15 days of cultivation at 20°C, 10 mL was transferred to 90 mL of fresh L1-Si medium in 300-mL Erlenmeyer flasks and placed in a climate chamber set to the desired temperature (26 or 30°C) and exhibiting the same light intensity and light:dark cycle. These temperatures are already common in the Red Sea [121] or near the equator [122], and are well within the range predicted for the future oceans [37]. Replicate cultures were set up under identical conditions to allow sampling of 3 biological replicates each for transcriptome and proteome and 5 biological replicates for metabolome. For proteome analysis, up to 12 L of culture (120 flasks each with 100 mL culture) was cultivated in the same climate chamber to obtain ~ 2 g biomass (wet weight). For each growth stage (exponential, stationary) and temperature (20°C, 26°C, 30°C), a complete set of cultures were sacrificed. Cell counting and harvesting were started about 5 h after the onset of the light period. For cell counting, random samples were chosen from the climate chamber to account for slight differences in light intensity.

For transcriptome analysis, three 100 mL cultures (biological replicates) were sampled per temperature and growth phase. Each culture was centrifuged in two 50-mL Falcon tubes at (4276 g, 4°C, 5 min) in a Heraeus Multifuge™ X1R. The supernatant was decanted, and the pellet was resuspended in the remaining medium. The two pellets were combined in an Eppendorf tube (2 mL) and centrifuged (17,000* g*, 4°C, 3 min) in a Heraeus Primo R centrifuge. The remaining supernatant was removed by pipetting, and the weight of the wet pellet was determined. The pellet was resuspended in 1 mL TRIzol reagent (Thermo Fisher Scientific, Waltham MA, USA), snap frozen in liquid nitrogen and stored at −70°C until further analysis.

For proteome analysis, tubes and buffers used in the following steps were pre-chilled; all steps were conducted on ice. The culture (400 mL) was filled into pre-chilled 500-mL centrifuge bottles, centrifuged (4248* g*, 4°C, 30 min) using a Sorval Lynx 4000 (Thermo Fischer). The supernatant was decanted, the pellet was resuspended in a buffer (100 mL) containing Tris–HCl (100 mM, pH 7.5) and MgCl_2_∙6H_2_O (5 mM). The resuspended pellets were centrifuged (4248* g*, 4°C, 30 min), and the supernatant was removed. The pellet was resuspended by gently pipetting in the same buffer (800 µL). The suspension was transferred into 2-mL Eppendorf tubes and centrifuged (17,000* g*, 4°C, 5 min) using a Heraeus Primo R centrifuge. The supernatant was removed by pipetting and the weight of the wet pellet was determined. Samples were frozen in liquid nitrogen and stored at −70°C.

For metabolome analysis, 15 mL from five cultures (100 mL each; as biological replicates) from each temperature and growth phase was extracted immediately after cell counting with a filtration unit and 0.22 µm Millipore membrane filter. Samples were filtered at 500 mbar with a vacuum controller. The cells were washed three times with 4°C cold 3.5% NaCl.

The filters were immediately transferred to 5-mL Eppendorf tubes containing 100 mg of glass beads (0.7–100 µm; Kuhmichel, Ratingen, Germany), three stainless steel beads (two 5 mm^3^ and one 10 mm^3^; Kugel Winnie, Bamberg, Germany) for partially destroying the filter and to obtain cell lysis. Cold extraction fluid (2 mL, per-chilled at −20°C) was immediately added; this extraction fluid for metabolite extraction contained methanol, ethanol, and chloroform [123], and the internal standard ^13^C-ribitol. The pre-chilled 5-mL tubes with the filter and extraction fluid on ice were mixed for 20 s and placed back on ice until further treatment.

### Analysis of transcriptome and differentially expressed genes

The transcriptome reads were mapped to the assembled reference genome using HISAT2 [93]. The reads mapped onto the exons were counted for the corresponding genes with featureCounts [50]. For differential expression analysis, only uniquely mapped reads were used to avoid ambiguity. The fragments per kilobase of transcript per million mapped fragments (FPKM) value of each gene was calculated by normalizing the fragments per million with the sum of the exon length of the corresponding gene. The ternary visualization of the gene expression pattern across three temperatures was produced with R package *ggtern* [124].

For analysis of differentially expressed genes (DEGs), genes with low expression were filtered out by the *filterbyexpr* function in edgeR [125] using default parameters. Then, the DEG analysis was performed with edgeR using the recommended *glmQLF* test on the raw read count per gene. Genes with a Benjamini–Hochberg corrected *p*-value ≤ 0.001 and an absolute log_2_(fold-change) ≥ 2 were considered as significantly differentially expressed.

Hierarchical clustering with the complete-linkage algorithm was used to identify gene clusters based on their expression profile across temperature conditions. The input expression values were centered log_2_-transformed FPKM values, i.e., log_2_(FPKM + 1) centered by subtracting the mean. The tree was cut into eight clusters to represent different expression profiles. Superclusters 4 and 7 in the stationary phase (each with small number of genes) showed very similar expression patterns; these were merged for downstream functional enrichment analysis.

### Functional analysis of DEGs

The GO term enrichment analysis was carried out with the R package topGO [126] for all three gene ontologies, i.e., Biological Process (BP), Cellular omponent (CC), and Molecular Function (MF). REVIGO [127] was used to summarize the GO terms according to the semantic similarity for a concise visualization (Additional File 2: Fig. S13). In order to perform a KEGG pathway enrichment analysis, we assigned KEGG ortholog (KO) number for each gene using Kofam [51]. When a gene had multiple KO assignments with an e-value ≤ 10^−10^, we chose the one with the lowest e-value. If a gene had several KO assignments with an e-value of zero, we kept all those KO assignments for this gene. Using this annotation, the genes with KO assigned held about 50% of mapped reads. The expression profile for each KO gene was then generated by summing up the read count for all genes belonging to the same KO gene. Differentially expressed KO genes were then identified using edgeR similar to the above DEG analysis. The KEGG pathway and module enrichment analyses were performed using clusterProfiler [128] on the DEGs. For both GO term enrichment analysis and KEGG enrichment analysis, a false discovery rate (FDR) ≤ 0.05 was considered as significantly enriched.

In the central carbon metabolism pathway analysis, a paired Wilcoxon test was performed to compare the expression change of the gene members belonging to the same EC number. The alteration of a metabolite’s concentration was analyzed using Wilcoxon test on the mean concentration values of biological replicates over the three technical replicates; FDR ≤ 0.05 was regarded as significantly changed.

### Proteome analysis

Cells of *P. cordatum* were resuspended in solubilization buffer and disrupted by bead beating (FastPrep-24 5G, MP Biomedicals) for 10 s at 6 m/s followed by 90 s on ice (in three repetitions) using 0.1 mm silica spheres. Cell debris and insoluble material were removed by ultracentrifugation (104,000* g*, 1 h, 17°C) and the supernatant was again centrifuged (200,000* g*, 1 h, 17). The protein content of the preparation was determined according to Bradford [129]; 50 µg was subjected to reduction, alkylation, and tryptic digestion as reported previously [130]. Generated peptides were analyzed by (a) one-dimensional (1D) reversed phase nanoLC and (b) two dimensional (2D) SCX-/RP nanoLC separation coupled to MS-detection. For 1D separation, tryptic peptides (1 mg) were decomplexed by reversed phase nanoLC separation (Ultimate 3000 nanoRSLC; Thermo Fisher Scientific, Dreieich, Germany) using a trap column setup (2 cm length, 5 µm bead size, 75 µm inner diameter; Thermo Fisher Scientific) with a 25-cm separation column (2 µm bead size, 75 µm inner diameter; Thermo Fisher Scientific), and applying a 360-min linear gradient [130]. In case of 2D separation, 4 µg was subjected to SCX fractionation (eluent A: 5 mM H_3_PO_4_, 5% v/v acetonitrile, pH 3.0; eluent B: 5 mM H_3_PO_4_, 5% v/v acetonitrile, 1 M NaCl, pH 3.0) with a linear 20 min gradient and collection of 19 fractions per sample beginning 5 min after injection. Each fraction was subsequently applied for second dimension reversed phase separation (see above) using a 60 min gradient. For both methods, the eluent was continuously ionized (captive spray ion source; Bruker Daltonik GmbH, Bremen, Germany) and ions analyzed by an ion-trap mass spectrometer (amaZon speed ETD; Bruker Daltonik GmbH) as described in Wöhlbrand et al. [131].

To prepare the membrane protein-enriched fraction, cell pellets were gently thawed, resuspended in 0.5 mL membrane lysis buffer (MLB; 100 mM Tris–HCl, 2 mM MgCl_2_, 10% (w/v) glycerin, 0.5 mM DTT, pH 7.5), and disrupted by bead beating (as described above). DNA was digested using DNase I and the obtained raw fraction applied on top of a continuous sucrose gradient (30–80% w/v) prior to centrifugation (37,800* g*, 12 h, 4°C). Membrane containing fractions were collected and washed twice with MLB (centrifugation at 104,000* g*, 1 h, 4°C). Obtained membrane pellets were resuspended in MLB (500 µL), pooled, and centrifugation repeated. The final pellet was resuspended in sodium dodecyl sulfate (SDS, 300 µL, 1.0% w/v) and incubated at 95°C (5 min) prior to centrifugation (20,000* g*, 20 min, 20°C). The supernatant was snap frozen in liquid nitrogen until further analysis. Protein content was determined using the RC-DC™ Protein Assay (BioRad GmbH, Munich, Germany). A total of 10 µg protein per fraction of each sample was separated by SDS–polyacrylamide gel electrophoresis (SDS-PAGE), and gels were stained with Coomassie brilliant blue [132]. Each sample lane was cut into 8 slices, and each slice into small pieces of ~ 1–2 mm^2^ for subsequent in-gel digest as previously described [131]. The generated peptide solutions were analyzed by reversed phase nanoLC-MS (as described above), via a 120-min gradient. Per sample, protein search results of each slice of fractions I and II were compiled.

Protein identification was performed using Mascot (version 2.3; Matrix Science, London, UK) via the ProteinScape platform (version 4.2; Bruker Daltonik GmbH) against the genomic database of *P. cordatum*. A target-decoy strategy with a false discovery rate < 1.0% was applied together with following settings: enzyme = trypsin, missed cleavage allowed = 1, carbamidomethylation (*C*) = fixed, oxidation (*M*) = variable modification, peptide and MS/MS mass tolerance = 0.3 Da, monoisotopic mode, peptide charge = 2 + and 3 + , instrument type = ESI-TRAP, significance threshold *p* < 0.05, ion score cutoff = 25.0, and minimum peptide length = 5. Search results of individual searches per sample were compiled using the ProteinExtractor function of ProteinScape.

For data analysis, spectral counts for the identified proteins were determined per sample and experimental approach (i.e., membrane protein-enriched fraction, 1D and 2D separated shotgun). Per sample replicate, only the maximum spectral count of one of the three approaches per protein was considered for subsequent analyses, and only proteins detected in at least two replicate samples were included. Custom Matlab (version 2021a; MathWorks, Natick, MA, USA) code was used for data analyses. Following quantile normalization, ANOVA *p*-value and fold-change were calculated for each protein. Proteins that exhibit significant changes (*p* ≤ 0.05) in distinct conditions were considered to be differentially expressed.

### Metabolome analysis

The cell disruption was performed with MM400 oscillating mill (Retsch, Haan, Germany) at 30 Hz for 2 min, and repeated three times. The cooling during the treatment was ensured by using −80°C pre-chilled sample containers. For metabolite extraction, the tubes were centrifuged (14,489* g*, 8 min, 4°C). Two volumes (100 µL and 200 µL) of the supernatant were transferred to gas chromatography (GC) glass vials (Klaus Trott Chromatographiezubehör, Kriftel, Germany) and dried under vacuum (Labconco, Kansas City, Missouri, USA) at 4°C. After drying overnight, the vials were capped with magnetic vial caps (Klaus Ziemer GmbH, Langerwehe, Germany) and stored at −80°C until measurements. All samples belonging to one experiment were analyzed in a batch.

To analyze polar metabolites with gas chromatography and mass spectrometry (GC–MS), a derivatization was performed to reduce polar interactions by introducing non-polar trimethylsilyl groups. This was necessary to evaporate the substances under low temperatures and avoid alteration. After extraction, the samples were analyzed with a gas chromatograph connected to a mass spectrometer (Agilent 7890A and 5975C inert XL Mass Selective Detector). The sample derivatization was automatically done with a multisampler (GERSTEL, Mühlheim an der Ruhr, Germany) and 2% methoxyamine hydrochloride (MeOX; 15 µL) in pyridine (Roth AG, Arlesheim, Switzerland) for 60 min at 40°C followed by the addition of 15 µL *N*-tert-butyldimethylsilyl-*N*-methyl and incubated at 40°C (30 min). For measurement, the sample (1 µL) was injected in splitless-mode into a split/splitless (SSL) injector at 270°C. The GC was equipped with a 30 m DB-35MS + 5 m DuraGuard capillary column (0.25 mm inner diameter, 0.25 µm film thickness; Agilent Technologies). Helium was used as carrier gas at a flow rate of 1.0 mL min^−1^ and the GC-oven was run at following temperatures and times per sample: 6 min at 80°C; 6 min from 80 to 300°C; 10 min at 300°C; 2.5 min from 300 to 325°C; and 4 min at 325°C. Each GC–MS run lasted 60 min. The transmission temperature from GC to MS was 280°C and the temperatures of the MS and the quadrupole were 230 and 150°C, respectively. The ionization in the mass detector was performed at 70 eV. The detector was operated in SCAN-mode for full scan spectra from 70 to 800 m*/z* with 2 scans s^−1^. For calibration of retention indices, the alkane standard mixture (C_10_-C_40_; Sigma-Aldrich) was used.

Data analysis was performed using MetaboliteDetector [133]. After calibration with the alkane mixture, a normalization by ^13^C-ribitol was performed to eliminate variations in sample volumes. Batch quantifications were performed with MetaboliteDetector. Non-targeted analysis was performed with an in-house library and the following settings: peak threshold 5, minimum peak height 5, 10 bins per scan, deconvolution with 5, no baseline adjustment, compound reproducibility 1.00, maximum peak of discrimination index 100.00, and minimum number of ions = 20.

### Supplementary Information

**Additional file 1.** Supplementary Note.

**Additional file 2.** Supplementary Figures S1-S20.

**Additional file 3.** Supplementary Tables S1-S29.

**Additional file 4.** Supplementary Data S1.

**Additional file 5.** Peer review history.

## Data Availability

All genome and transcriptome data generated from this study are available at NCBI via BioProject accession PRJEB54915. The assembled genome and transcriptome sequences, and the predicted gene and protein sequences from *P. cordatum* are available at https://doi.org/10.48610/bc851b7 [38]. The assembled genome is available at NCBI via accession GCA_963575745.1, and the six assembled transcriptomes via accessions HCBB00000000.1, HCBC00000000.1, HCBD00000000.1, HCBE00000000.1, HCBF00000000.1, and HCBG00000000.1. The mass spectrometry proteomics data have been deposited to the ProteomeXchange Consortium via the PRIDE [134] partner repository with the dataset identifier PXD046193. All proteome and metabolome data including detailed methodology are available at FAIRDOMHub [135]: https://doi.org/10.15490/fairdomhub.1.investigation.565.1 [58]. For ease of access, all supplementary files associated with this paper are also available at https://doi.org/10.5281/zenodo.10021821 [136].

## References

[1] Brown AR, Lilley M, Shutler J, Lowe C, Artioli Y, Torres R (2020). Assessing risks and mitigating impacts of harmful algal blooms on mariculture and marine fisheries. Rev Aquac.

[2] Wells ML, Karlson B, Wulff A, Kudela R, Trick C, Asnaghi V (2020). Future HAB science: directions and challenges in a changing climate. Harmful Algae.

[3] Karlson B, Andersen P, Arneborg L, Cembella A, Eikrem W, John U (2021). Harmful algal blooms and their effects in coastal seas of Northern Europe. Harmful Algae.

[4] Murray SA, Kohli GS, Farrell H, Spiers ZB, Place AR, Dorantes-Aranda JJ (2015). A fish kill associated with a bloom of *Amphidinium carterae* in a coastal lagoon in Sydney Australia. Harmful Algae.

[5] Gobler CJ (2020). Climate change and harmful algal blooms: insights and perspective. Harmful Algae.

[6] Taylor FJR, Hoppenrath M, Saldarriaga JF (2008). Dinoflagellate diversity and distribution. Biodivers Conserv.

[7] LaJeunesse TC, Parkinson JE, Gabrielson PW, Jeong HJ, Reimer JD, Voolstra CR (2018). Systematic revision of Symbiodiniaceae highlights the antiquity and diversity of coral endosymbionts. Curr Biol.

[8] Stoecker DK, Hansen PJ, Caron DA, Mitra A (2017). Mixotrophy in the marine plankton. Annu Rev Mar Sci.

[9] Rädecker N, Pogoreutz C, Gegner HM, Cardenas A, Roth F, Bougoure J (2021). Heat stress destabilizes symbiotic nutrient cycling in corals. Proc Natl Acad Sci U S A.

[10] Johnson JG, Morey JS, Neely MG, Ryan JC, Van Dolah FM (2012). Transcriptome remodeling associated with chronological aging in the dinoflagellate *Karenia brevis*. Marine Genomics.

[11] Shi X, Lin X, Li L, Li M, Palenik B, Lin S (2017). Transcriptomic and microRNAomic profiling reveals multi-faceted mechanisms to cope with phosphate stress in a dinoflagellate. ISME J.

[12] Wang X, Niu X, Chen Y, Sun Z, Han A, Lou X (2019). Transcriptome sequencing of a toxic dinoflagellate, *Karenia mikimotoi* subjected to stress from solar ultraviolet radiation. Harmful Algae.

[13] LaJeunesse TC, Lambert G, Andersen RA, Coffroth MA, Galbraith DW (2005). *Symbiodinium* (Pyrrhophyta) genome sizes (DNA content) are smallest among dinoflagellates. J Phycol.

[14] Saad OS, Lin X, Ng TY, Li L, Ang P, Lin S (2020). Genome size, rDNA copy, and qPCR assays for Symbiodiniaceae. Front Microbiol.

[15] Lin S (2011). Genomic understanding of dinoflagellates. Res Microbiol.

[16] Wisecaver JH, Hackett JD (2011). Dinoflagellate genome evolution. Annu Rev Microbiol.

[17] Shoguchi E, Shinzato C, Kawashima T, Gyoja F, Mungpakdee S, Koyanagi R (2013). Draft assembly of the *Symbiodinium minutum* nuclear genome reveals dinoflagellate gene structure. Curr Biol.

[18] Lin S, Cheng S, Song B, Zhong X, Lin X, Li W (2015). The *Symbiodinium kawagutii* genome illuminates dinoflagellate gene expression and coral symbiosis. Science.

[19] Aranda M, Li Y, Liew YJ, Baumgarten S, Simakov O, Wilson MC (2016). Genomes of coral dinoflagellate symbionts highlight evolutionary adaptations conducive to a symbiotic lifestyle. Sci Rep.

[20] Liu H, Stephens TG, González-Pech RA, Beltran VH, Lapeyre B, Bongaerts P (2018). *Symbiodinium* genomes reveal adaptive evolution of functions related to coral-dinoflagellate symbiosis. Commun Biol.

[21] González-Pech RA, Stephens TG, Chen Y, Mohamed AR, Cheng Y, Shah S (2021). Comparison of 15 dinoflagellate genomes reveals extensive sequence and structural divergence in family Symbiodiniaceae and genus *Symbiodinium*. BMC Biol.

[22] Shah S, Dougan KE, Chen Y, Bhattacharya D, Chan CX (2023). Gene duplication is the primary driver of intraspecific genomic divergence in coral algal symbionts. Open Biol.

[23] Beedessee G, Kubota T, Arimoto A, Nishitsuji K, Waller RF, Hisata K (2020). Integrated omics unveil the secondary metabolic landscape of a basal dinoflagellate. BMC Biol.

[24] Camp EF, Kahlke T, Signal B, Oakley CA, Lutz A, Davy SK (2022). Proteome metabolome and transcriptome data for three Symbiodiniaceae under ambient and heat stress conditions. Sci Data.

[25] Dougan KE, González-Pech RA, Stephens TG, Shah S, Chen Y, Ragan MA (2022). Genome-powered classification of microbial eukaryotes: focus on coral algal symbionts. Trends Microbiol.

[26] Zaheri B, Morse D (2021). Assessing nucleic acid binding activity of four dinoflagellate cold shock domain proteins from *Symbiodinium kawagutii* and *Lingulodinium polyedra*. BMC Mol Cell Biol.

[27] Wong JTY (2019). Architectural organization of dinoflagellate liquid crystalline chromosomes. Microorganisms.

[28] Levin RA, Beltran VH, Hill R, Kjelleberg S, McDougald D, Steinberg PD (2016). Sex, scavengers, and chaperones: transcriptome secrets of divergent *Symbiodinium* thermal tolerances. Mol Biol Evol.

[29] Liew YJ, Li Y, Baumgarten S, Voolstra CR, Aranda M (2017). Condition-specific RNA editing in the coral symbiont *Symbiodinium microadriaticum*. PLoS Genet.

[30] Mohamed AR, Andrade N, Moya A, Chan CX, Negri AP, Bourne DG (2020). Dual RNA-sequencing analyses of a coral and its native symbiont during the establishment of symbiosis. Mol Ecol.

[31] Zhang H, Hou Y, Miranda L, Campbell DA, Sturm NR, Gaasterland T (2007). Spliced leader RNA trans-splicing in dinoflagellates. Proc Natl Acad Sci U S A.

[32] Mungpakdee S, Shinzato C, Takeuchi T, Kawashima T, Koyanagi R, Hisata K (2014). Massive gene transfer and extensive RNA editing of a symbiotic dinoflagellate plastid genome. Genome Biol Evol.

[33] Velikova V, Larsen J (1999). The *Prorocentrum cordatum*/*Prorocentrum minimum* taxonomic problem. Grana.

[34] Zhang F, Li M, Glibert PM, Ahn SH (2021). A three-dimensional mechanistic model of *Prorocentrum minimum* blooms in eutrophic Chesapeake Bay. Sci Total Environ.

[35] Khanaychenko AN, Telesh IV, Skarlato SO (2019). Bloom-forming potentially toxic dinoflagellates *Prorocentrum cordatum* in marine plankton food webs. Protistology.

[36] Seebens H, Schwartz N, Schupp PJ, Blasius B (2016). Predicting the spread of marine species introduced by global shipping. Proc Natl Acad Sci U S A.

[37] Alexander MA, Scott JD, Friedland KD, Mills KE, Nye JA, Pershing AJ (2018). Projected sea surface temperatures over the 21st century: changes in the mean, variability and extremes for large marine ecosystem regions of Northern Oceans. Elementa.

[38] Dougan KE, Deng ZL, Wöhlbrand L, Reuse C, Bunk B, Chen Y, et al. Genome and transcriptome data for bloom-forming dinoflagellate *Prorocentrum cordatum* CCMP1329. Datasets. The University of Queensland Data Collection. 2023. 10.48610/bc851b7.

[39] Stephens TG, González-Pech RA, Cheng Y, Mohamed AR, Burt DW, Bhattacharya D (2020). Genomes of the dinoflagellate *Polarella glacialis* encode tandemly repeated single-exon genes with adaptive functions. BMC Biol.

[40] Dougan KE, Bellantuono AJ, Kahlke T, Abbriano RM, Chen Y, Shah S, et al. Whole-genome duplication in an algal symbiont serendipitously confers thermal tolerance to corals. bioRxiv*.* 2022:2022.04.10.487810.

[41] Chen Y, Shah S, Dougan KE, van Oppen MJH, Bhattacharya D, Chan CX (2022). Improved *Cladocopium goreaui* genome assembly reveals features of a facultative coral symbiont and the complex evolutionary history of dinoflagellate genes. Microorganisms.

[42] John U, Lu Y, Wohlrab S, Groth M, Janouškovec J, Kohli GS (2019). An aerobic eukaryotic parasite with functional mitochondria that likely lacks a mitochondrial genome. Sci Adv.

[43] Farhat S, Le P, Kayal E, Noel B, Bigeard E, Corre E (2021). Rapid protein evolution, organellar reductions, and invasive intronic elements in the marine aerobic parasite dinoflagellate *Amoebophrya* spp. BMC Biol.

[44] Worden AZ, Lee JH, Mock T, Rouzé P, Simmons MP, Aerts AL (2009). Green evolution and dynamic adaptations revealed by genomes of the marine picoeukaryotes *Micromonas*. Science.

[45] Huff JT, Zilberman D, Roy SW (2016). Mechanism for DNA transposons to generate introns on genomic scales. Nature.

[46] van der Burgt A, Severing E, De Wit PJGM, Collemare J (2012). Birth of new spliceosomal introns in fungi by multiplication of introner-like elements. Curr Biol.

[47] Stephens TG, Ragan MA, Bhattacharya D, Chan CX (2018). Core genes in diverse dinoflagellate lineages include a wealth of conserved dark genes with unknown functions. Sci Rep.

[48] Bachvaroff TR, Place AR (2008). From stop to start: tandem gene arrangement, copy number and trans-splicing sites in the dinoflagellate *Amphidinium carterae*. PLoS One.

[49] Pachiadaki MG, Brown JM, Brown J, Clair JJL, Chisholm SW, Bezuidt O (2019). Charting the complexity of the marine microbiome through single-cell genomics. Cell.

[50] Liao Y, Smyth GK, Shi W (2014). FeatureCounts: an efficient general purpose program for assigning sequence reads to genomic features. Bioinformatics.

[51] Aramaki T, Blanc-Mathieu R, Endo H, Ohkubo K, Kanehisa M, Goto S (2020). KofamKOALA: KEGG Ortholog assignment based on profile HMM and adaptive score threshold. Bioinformatics.

[52] Slamovits CH, Keeling PJ (2008). Widespread recycling of processed cDNAs in dinoflagellates. Curr Biol.

[53] Butler CC, Turnham KE, Lewis AM, Nitschke MR, Warner ME, Kemp DW (2023). Formal recognition of host-generalist species of dinoflagellate (*Cladocopium*, Symbiodiniaceae) mutualistic with Indo-Pacific reef corals. J Phycol.

[54] Amphidinium ver. 1.0. https://marinegenomics.oist.jp/amphidinium/viewer/download?project_id=83. Accessed 14 Dec 2022.

[55] Roy SW, Gozashti L, Bowser BA, Weinstein BN, Larue GE, Corbett-Detig R (2023). Intron-rich dinoflagellate genomes driven by Introner transposable elements of unprecedented diversity. Curr Biol.

[56] Song B, Morse D, Song Y, Fu Y, Lin X, Wang W (2017). Comparative genomics reveals two major bouts of gene Retroposition coinciding with crucial periods of Symbiodinium evolution. Genome Biol Evol.

[57] Tester PA, Litaker RW, Berdalet E (2020). Climate change and harmful benthic microalgae. Harmful Algae.

[58] Dougan KE, Deng ZL, Wöhlbrand L, Reuse C, Bunk B, Chen Y (2023). Heat stress response of *Prorocentrum cordatum* - proteome and metabolome. Datasets FAIRDOMHub.

[59] Singh A, Shannon CP, Gautier B, Rohart F, Vacher M, Tebbutt SJ (2019). DIABLO: an integrative approach for identifying key molecular drivers from multi-omics assays. Bioinformatics.

[60] Salvucci ME, Crafts-Brandner SJ (2004). Inhibition of photosynthesis by heat stress: the activation state of Rubisco as a limiting factor in photosynthesis. Physiol Plant.

[61] Schroda M, Hemme D, Mühlhaus T (2015). The *Chlamydomonas* heat stress response. Plant J.

[62] Verma A, Barua A, Ruvindy R, Savela H, Ajani PA, Murray SA (2019). The genetic basis of toxin biosynthesis in dinoflagellates. Microorganisms.

[63] Shi X, Zhang H, Lin S (2013). Tandem repeats, high copy number and remarkable diel expression rhythm of form II RuBisCO in *Prorocentrum donghaiense* (Dinophyceae). PLoS One.

[64] Lee MG (1998). The 3' untranslated region of the hsp 70 genes maintains the level of steady state mRNA in *Trypanosoma brucei* upon heat shock. Nucleic Acids Res.

[65] Quijada L, Soto M, Alonso C, Requena JM (2000). Identification of a putative regulatory element in the 3'-untranslated region that controls expression of HSP70 in *Leishmania infantum*. Mol Biochem Parasitol.

[66] Zilka A, Garlapati S, Dahan E, Yaolsky V, Shapira M (2001). Developmental regulation of heat shock protein 83 in Leishmania. 3' processing and mRNA stability control transcript abundance, and translation id directed by a determinant in the 3'-untranslated region. J Biol Chem..

[67] Zhang H, Lin S (2003). Complex gene structure of the form II RuBisCo in the dinoflagellate *Prorocentrum minimum* (Dinophyceae). J Phycol.

[68] Bruce BD (2000). Chloroplast transit peptides: structure, function and evolution. Trends Cell Biol.

[69] Lee DW, Hwang I (2018). Evolution and design principles of the diverse chloroplast transit peptides. Mol Cell.

[70] Nassoury N, Cappadocia M, Morse D (2003). Plastid ultrastructure defines the protein import pathway in dinoflagellates. J Cell Sci.

[71] Patron NJ, Waller RF, Archibald JM, Keeling PJ (2005). Complex protein targeting to dinoflagellate plastids. J Mol Biol.

[72] Csurös M, Rogozin IB, Koonin EV (2008). Extremely intron-rich genes in the alveolate ancestors inferred with a flexible maximum-likelihood approach. Mol Biol Evol.

[73] Rogozin IB, Carmel L, Csuros M, Koonin EV (2012). Origin and evolution of spliceosomal introns. Biol Direct.

[74] Aumont O, Maury O, Lefort S, Bopp L (2018). Evaluating the potential impacts of the diurnal vertical migration by marine organisms on marine biogeochemistry. Global Biogeochem Cycles.

[75] Olsson P, Granéli E (1991). Observations on diurnal vertical migration and phased cell division for three coexisting marine dinoflagellates. J Plankton Res.

[76] Kamykowski D (1981). Laboratory experiments on the diurnal vertical migration of marine dinoflagellates through temperature gradients. Mar Biol.

[77] Sanchez-Garcia S, Wang H, Wagner-Döbler I (2022). The microbiome of the dinoflagellate *Prorocentrum cordatum* in laboratory culture and its changes at higher temperatures. Front Microbiol.

[78] Wohlrab S, Iversen MH, John U (2010). A molecular and co-evolutionary context for grazer induced toxin production in *Alexandrium tamarense*. PLoS One.

[79] Kang HC, Jeong HJ, Park SA, Ok JH, You JH, Eom SH (2021). Comparative transcriptome analysis of the phototrophic dinoflagellate *Biecheleriopsis adriatica* grown under optimal temperature and cold and heat stress. Front Mar Sci.

[80] Gallaher SD, Craig RJ, Ganesan I, Purvine SO, McCorkle SR, Grimwood J (2021). Widespread polycistronic gene expression in green algae. Proc Natl Acad Sci U S A.

[81] Ishida H, John U, Murray SA, Bhattacharya D, Chan CX (2023). Developing model systems for dinoflagellates in the post-genomic era. J Phycol.

[82] Strassert JFH, Irisarri I, Williams TA, Burki F (2021). A molecular timescale for eukaryote evolution with implications for the origin of red algal-derived plastids. Nat Commun.

[83] Guillard RRL, Hargraves PE (1993). *Stichochrysis immobilis* is a diatom, not a chrysophyte. Phycologia.

[84] Wood AM, Everroad RC, Wingard LM, Andersen RA (2005). Measuring growth rates in microalgal cultures. Algal Culturing Techniques.

[85] Levi-Setti R, Gavrilov KL, Rizzo PJ (2008). Divalent cation distribution in dinoflagellate chromosomes imaged by high-resolution ion probe mass spectrometry. Eur J Cell Biol.

[86] Zimin AV, Puiu D, Luo MC, Zhu T, Koren S, Marcais G (2017). Hybrid assembly of the large and highly repetitive genome of *Aegilops tauschii*, a progenitor of bread wheat, with the MaSuRCA mega-reads algorithm. Genome Res.

[87] Xue W, Li JT, Zhu YP, Hou GY, Kong XF, Kuang YY (2013). L_RNA_scaffolder: scaffolding genomes with transcripts. BMC Genomics.

[88] Wang M, Kong L (2019). pblat: a multithread blat algorithm speeding up aligning sequences to genomes. BMC Bioinformatics.

[89] Laetsch DR, Blaxter ML (2017). BlobTools: interrogation of genome assemblies. F1000Res..

[90] Chen S, Zhou Y, Chen Y, Gu J (2018). fastp: an ultra-fast all-in-one FASTQ preprocessor. Bioinformatics.

[91] Grabherr MG, Haas BJ, Yassour M, Levin JZ, Thompson DA, Amit I (2011). Full-length transcriptome assembly from RNA-Seq data without a reference genome. Nat Biotechnol.

[92] Li W, Godzik A (2006). Cd-hit: a fast program for clustering and comparing large sets of protein or nucleotide sequences. Bioinformatics.

[93] Kim D, Paggi JM, Park C, Bennett C, Salzberg SL (2019). Graph-based genome alignment and genotyping with HISAT2 and HISAT-genotype. Nat Biotechnol.

[94] Chen Y, González-Pech RA, Stephens TG, Bhattacharya D, Chan CX (2020). Evidence that inconsistent gene prediction can mislead analysis of dinoflagellate genomes. J Phycol.

[95] Dinoflagellate annotation workflow. https://github.com/TimothyStephens/Dinoflagellate_Annotation_Workflow. Accessed 14 Dec 2022.

[96] RepeatModeler. http://www.repeatmasker.org/RepeatModeler/. Accessed 14 Dec 2022.

[97] RepeatMasker. https://www.repeatmasker.org/. Accessed 14 Dec2022.

[98] Li H (2018). Minimap2: pairwise alignment for nucleotide sequences. Bioinformatics.

[99] Haas BJ, Delcher AL, Mount SM, Wortman JR, Smith RK, Hannick LI (2003). Improving the *Arabidopsis* genome annotation using maximal transcript alignment assemblies. Nucleic Acids Res.

[100] Remmert M, Biegert A, Hauser A, Söding J (2012). HHblits: lightning-fast iterative protein sequence searching by HMM-HMM alignment. Nat Methods.

[101] TransposonPSI. https://github.com/NBISweden/TransposonPSI. Accessed 14 Dec 2022.

[102] Just Annotate My Genome (JAMg). https://github.com/genomecuration/JAMg. Accessed 14 Dec 2022.

[103] Korf I (2004). Gene finding in novel genomes. BMC Bioinformatics.

[104] Hoff KJ, Stanke M (2019). Predicting genes in single genomes with AUGUSTUS. Curr Protoc Bioinform.

[105] dinoflag-alt-splice. https://github.com/chancx/dinoflag-alt-splice. Accessed 14 Dec 2022.

[106] Borodovsky M, Lomsadze A (2011). Eukaryotic gene prediction using GeneMark.hmm-E and GeneMark-ES. Curr Protoc Bioinform..

[107] Campbell MS, Holt C, Moore B, Yandell M (2014). Genome annotation and curation using MAKER and MAKER-P. Curr Protoc Bioinform..

[108] Haas BJ, Salzberg SL, Zhu W, Pertea M, Allen JE, Orvis J (2008). Automated eukaryotic gene structure annotation using EVidenceModeler and the p rogram to assemble spliced alignments. Genome Biol.

[109] Mŕazek J, Xie S (2006). Pattern locator: a new tool for finding local sequence patterns in genomic DNA sequences. Bioinformatics.

[110] Johnson LK, Alexander H, Brown CT (2019). Re-assembly, quality evaluation, and annotation of 678 microbial eukaryotic reference transcriptomes. GigaScience..

[111] MMETSP re-assemblies. 10.5281/zenodo.1212585. Accessed 14 Dec 2022.

[112] Emms DM, Kelly S (2019). OrthoFinder: phylogenetic orthology inference for comparative genomics. Genome Biol.

[113] bwa. https://github.com/lh3/bwa. Accessed 14 Dec 2022.

[114] Picard. https://broadinstitute.github.io/picard/. Accessed 14 Dec 2022.

[115] Piechotta M, Naarmann-de Vries IS, Wang Q, Altmuller J, Dieterich C (2022). RNA modification mapping with JACUSA2. Genome Biol.

[116] Katoh K, Standley DM (2013). MAFFT multiple sequence alignment software version 7: improvements in performance and usability. Mol Biol Evol.

[117] Capella-Gutiérrez S, Silla-Martínez JM, Gabaldón T (2009). trimAl: a tool for automated alignment trimming in large-scale phylogenetic analyses. Bioinformatics.

[118] Minh BQ, Schmidt HA, Chernomor O, Schrempf D, Woodhams MD, Von Haeseler A (2020). IQ-TREE 2: new nodels and efficient methods for phylogenetic inference in the genomic era. Mol Biol Evol.

[119] Rohart F, Gautier B, Singh A, Lê Cao KA (2017). mixOmics: an R package for ’omics feature selection and multiple data integration. PLoS Comput Biol.

[120] Zhou G, Soufan O, Ewald J, Hancock REW, Basu N, Xia J (2019). NetworkAnalyst 3.0: a visual analytics platform for comprehensive gene expression profiling and meta-analysis. Nucleic Acids Res..

[121] Voolstra CR, Valenzuela JJ, Turkarslan S, Cárdenas A, Hume BCC, Perna G (2021). Contrasting heat stress response patterns of coral holobionts across the Red Sea suggest distinct mechanisms of thermal tolerance. Mol Ecol.

[122] Ibarbalz FM, Henry N, Brandão MC, Martini S, Busseni G, Byrne H (2019). Global trends in marine plankton diversity across Kingdoms of life. Cell.

[123] Paul C, Mausz MA, Pohnert G (2013). A co-culturing/metabolomics approach to investigate chemically mediated interactions of planktonic organisms reveals influence of bacteria on diatom metabolism. Metabolomics.

[124] Hamilton NE, Ferry M (2018). ggtern: Ternary diagrams using ggplot2. J Stat Softw.

[125] Robinson Mark D, McCarthy Davis J, Smyth GK (2010). edgeR: a Bioconductor package for differential expression analysis of digital gene expression data. Bioinformatics.

[126] topGO. 10.18129/B9.bioc.topGO. Accessed 14 Dec 2022.

[127] Supek F, Bošnjak M, Škunca N, Šmuc T (2011). REVIGO summarizes and visualizes long lists of Gene Ontology terms. PLoS One.

[128] Yu G, Wang LG, Han Y, He QY (2012). ClusterProfiler: an R package for comparing biological themes among gene clusters. OMICS.

[129] Bradford MM (1976). A rapid and sensitive method for the quantitation of microgram quantities of protein utilizing the principle of protein-dye binding. Anal Biochem.

[130] Wöhlbrand L, Rabus R, Blasius B, Feenders C (2017). Influence of NanoLC column and gradient length as well as MS/MS frequency and sample complexity on shotgun protein identification of marine bacteria. J Mol Microbiol Biotechnol.

[131] Wöhlbrand L, Ruppersberg HS, Feenders C, Blasius B, Braun HP, Rabus R (2016). Analysis of membrane-protein complexes of the marine sulfate reducer *Desulfobacula toluolica* Tol2 by 1D blue native-PAGE complexome profiling and 2D blue native-/SDS-PAGE. Proteomics.

[132] Neuhoff V, Arold N, Taube D, Ehrhardt W (1988). Improved staining of proteins in polyacrylamide gels including isoelectric focusing gels with clear background at nanogram sensitivity using Coomassie Brilliant Blue G-250 and R-250. Electrophoresis.

[133] Hiller K, Hangebrauk J, Jäger C, Spura J, Schreiber K, Schomburg D (2009). Metabolite detector: comprehensive analysis tool for targeted and nontargeted GC/MS based metabolome analysis. Anal Chem.

[134] Perez-Riverol Y, Bai J, Bandla C, Garcia-Seisdedos D, Hewapathirana S, Kamatchinathan S (2022). The PRIDE database resources in 2022: a hub for mass spectrometry-based proteomics evidences. Nucleic Acids Res.

[135] Wolstencroft K, Krebs O, Snoep JL, Stanford NJ, Bacall F, Golebiewski M (2017). FAIRDOMHub: a repository and collaboration environment for sharing systems biology research. Nucleic Acids Res.

[136] Dougan KE, Deng ZL, Wöhlbrand L, Reuse C, et al. Multi-omics analysis reveals the molecular response to heat stress in a “red tide” dinoflagellate. 2023. Zenodo. 10.5281/zenodo.10021821. 10.1186/s13059-023-03107-4 PMC10666404 37996937

