# Supplementary material for: Multi-omics analysis reveals the molecular response to heat stress in a “red tide” dinoflagellate
Source: Genome Biol. 2023 Nov 23;24:265. doi: 10.1186/s13059-023-03107-4 (PMC10666404; doi:10.1186/s13059-023-03107-4)
Supplement: Supplementary file 2 — Additional file 2. Supplementary Figures S1-S20. [file 13059_2023_3107_MOESM2_ESM.pdf]

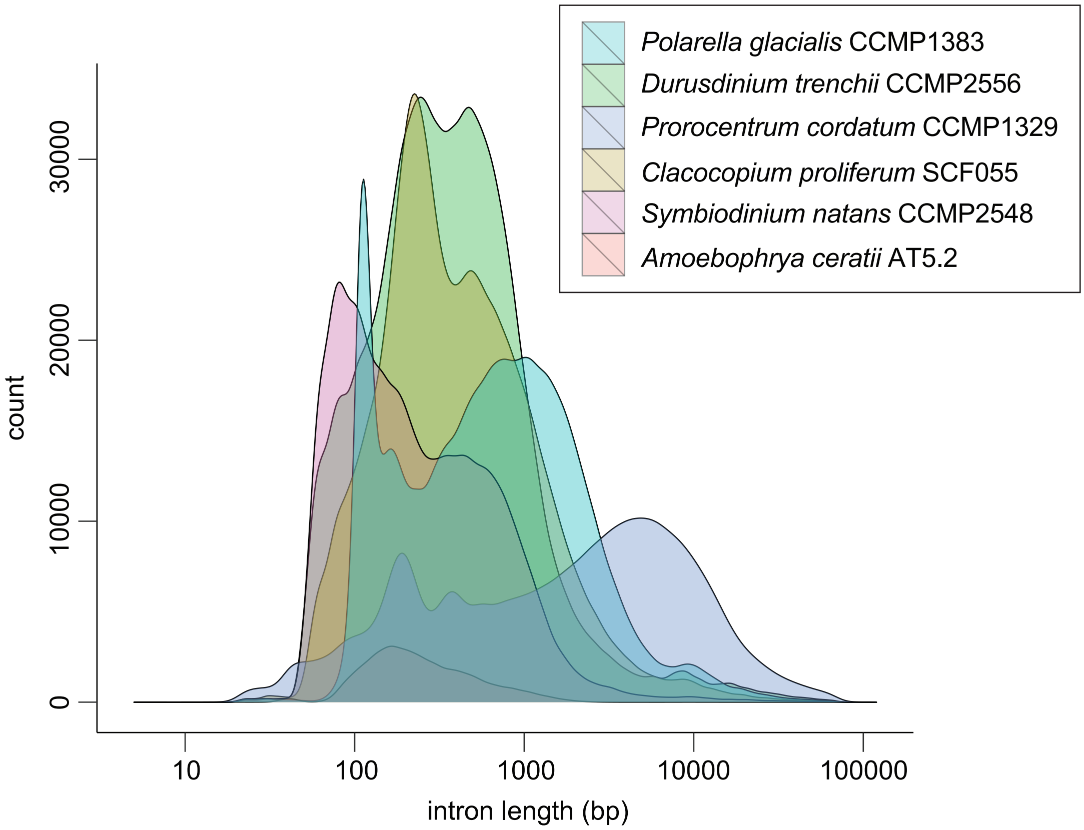


**Fig. S1.** Distribution of intron lengths in genomes of *P. cordatum* and other dinoflagellates.

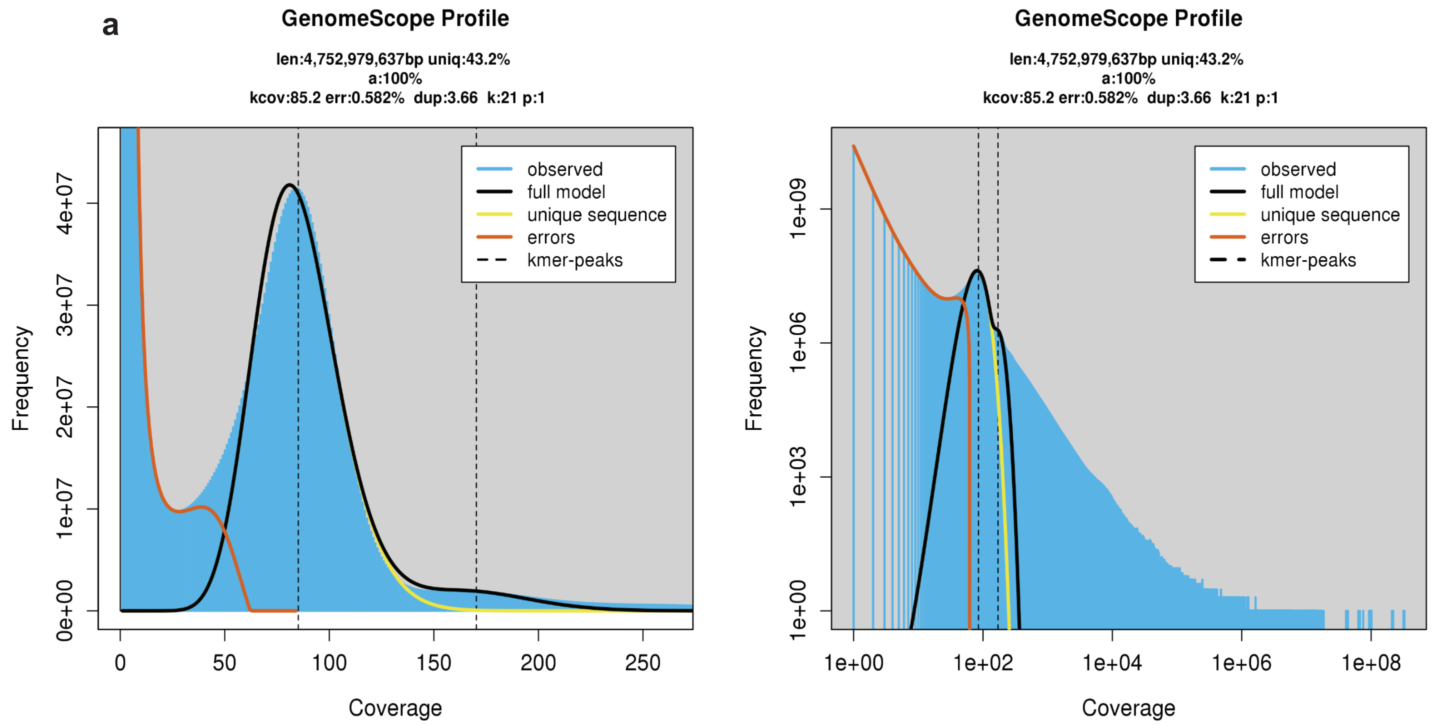


**b**

```
GenomeScope version 2.0
p = 1
k = 21

property                      min                 max
Homozygous (a)                100%                100%
Genome Haploid Length         4,730,452,501 bp    4,752,979,637 bp
Genome Repeat Length          2,685,886,986 bp    2,698,677,590 bp
Genome Unique Length          2,044,565,514 bp    2,054,302,047 bp
Model Fit                     42.9662%            90.8621%
Read Error Rate               0.582075%           0.582075%
```

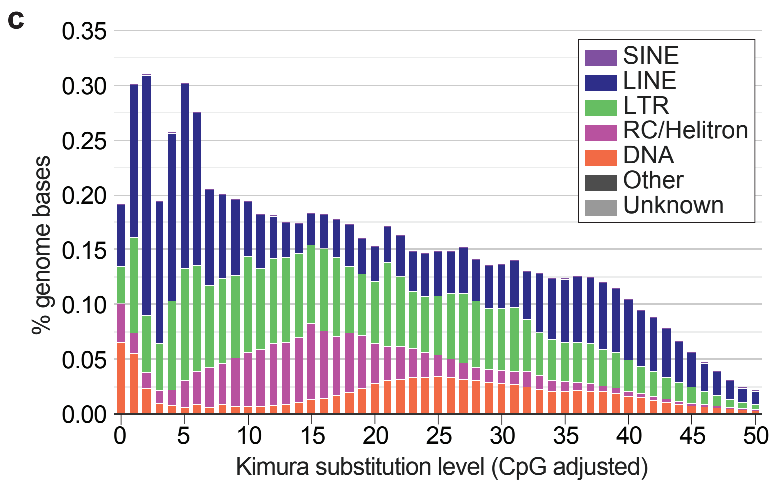


**Fig. S2.** Analysis of *P. cordatum* genome based on (a) distribution of 21-mers indicating a haploid genome, (b) genome-size estimation based on 21-mers using GenomeScope2, and (c) repeat landscape excluding unknown repeats.

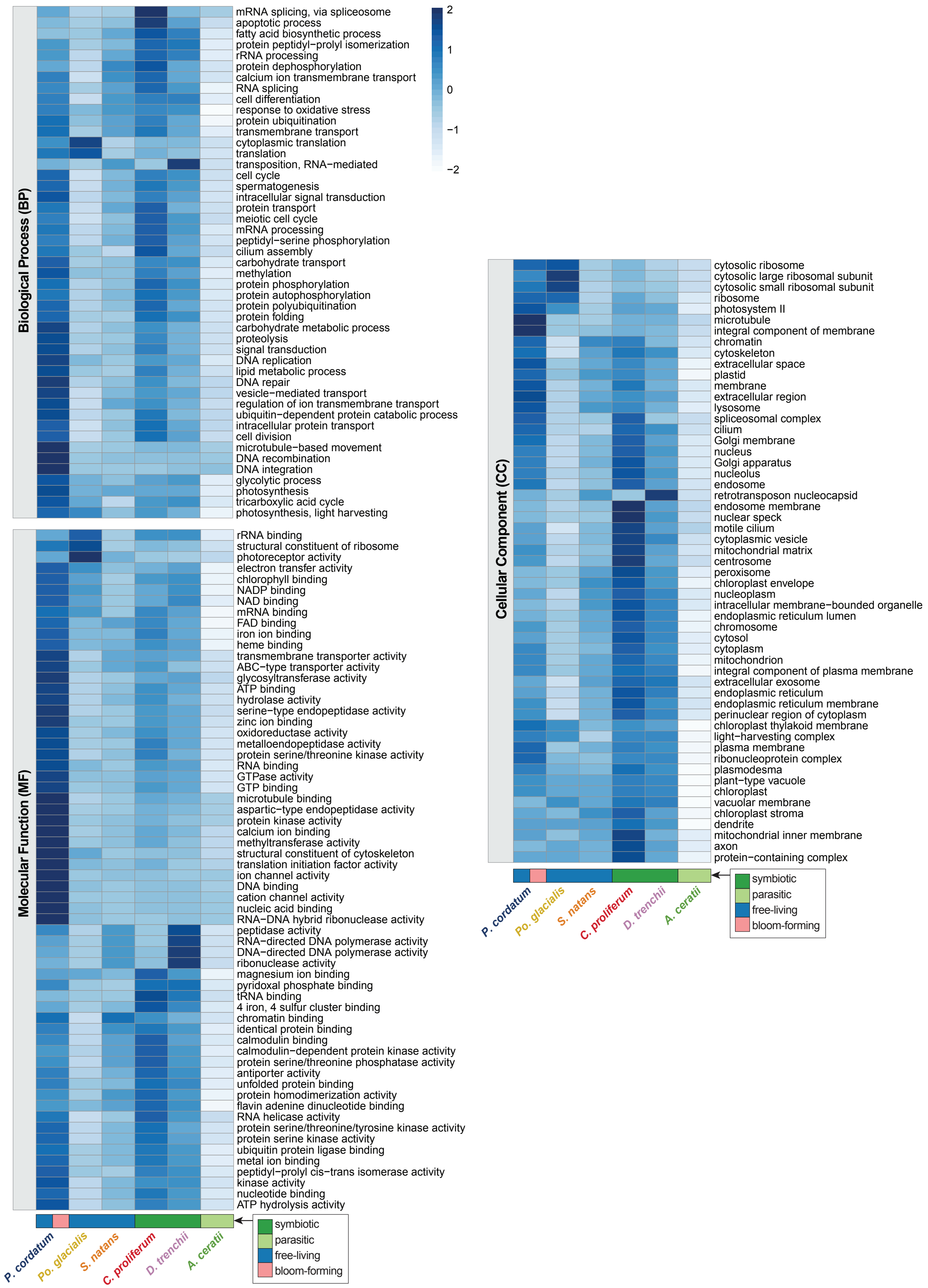


**Fig. S3.** Gene functions encoded in the genome of *P. cordatum* and the other five representative taxa based on count of Gene Ontology (GO) terms.

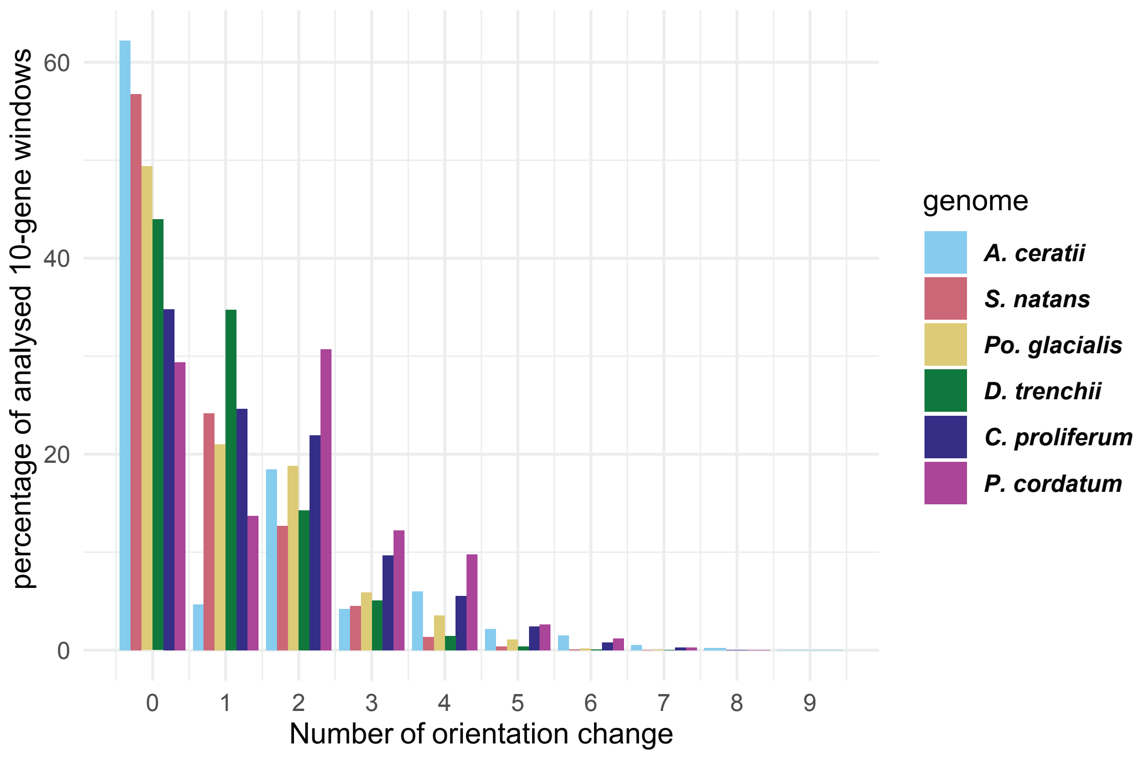


**Fig. S4.** Unidirectional coding of genes in dinoflagellate genomes based on gene-orientation changes in ten-gene windows.

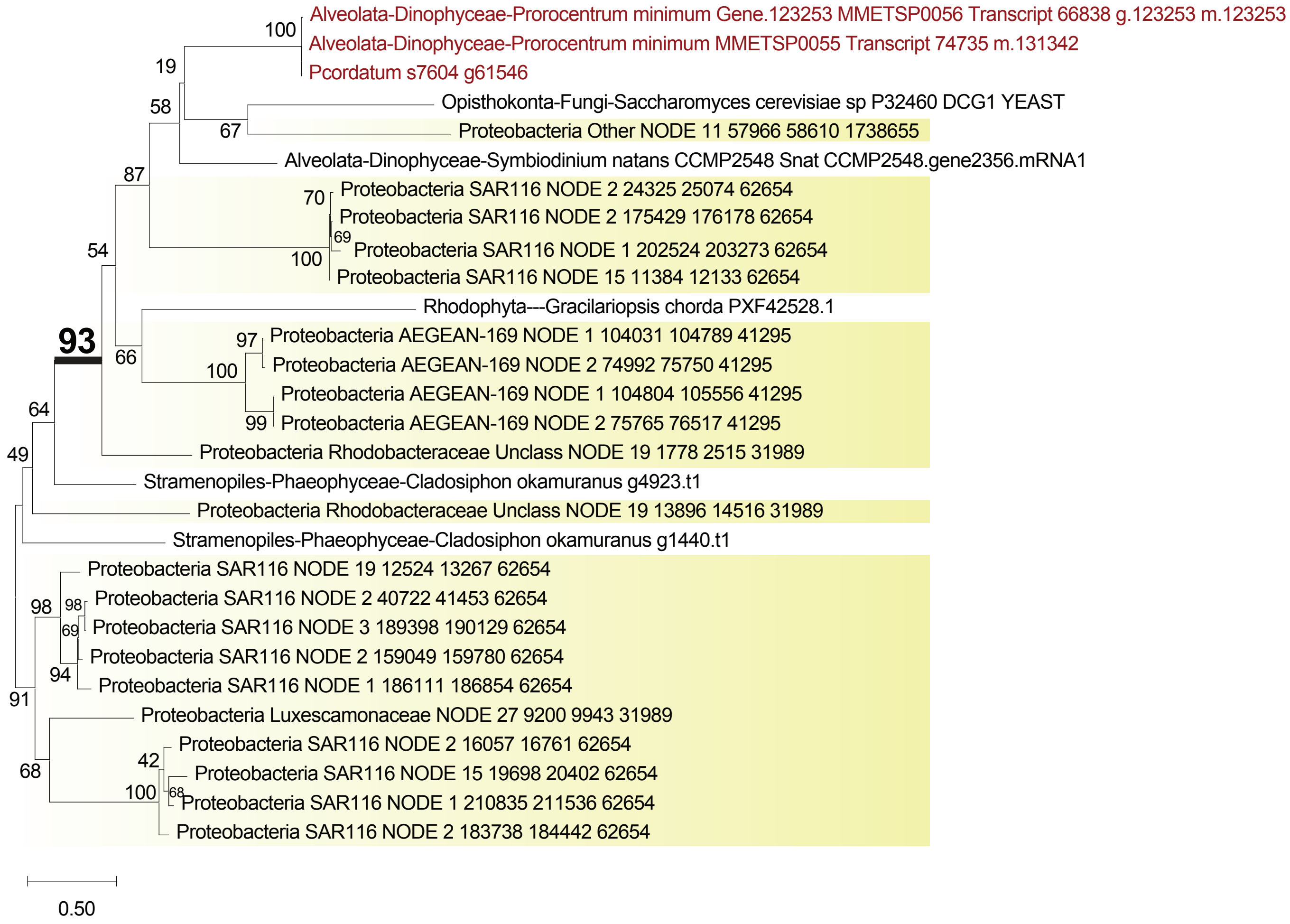


**Fig. S5.** Maximum likelihood protein tree of a putative hydantoin racemase indicating bacterial origin of the encoding gene in *P. cordatum*.

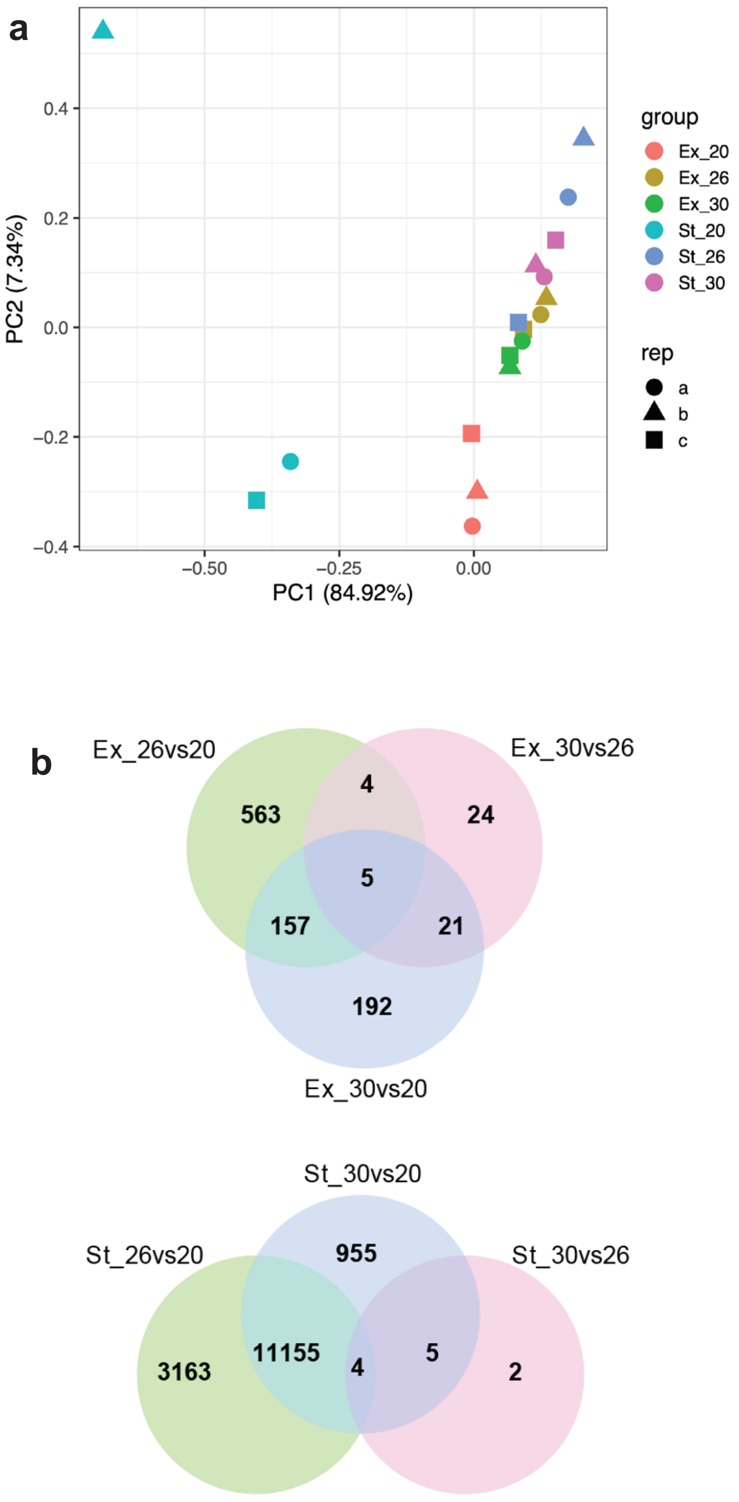


**Fig. S6.** Transcriptome analysis of *P. cordatum* showing (a) principal component analysis of samples based on FPKM values, and (b) Venn diagram detailing number of DEGs between any two temperature conditions, independently for Ex and St phases.

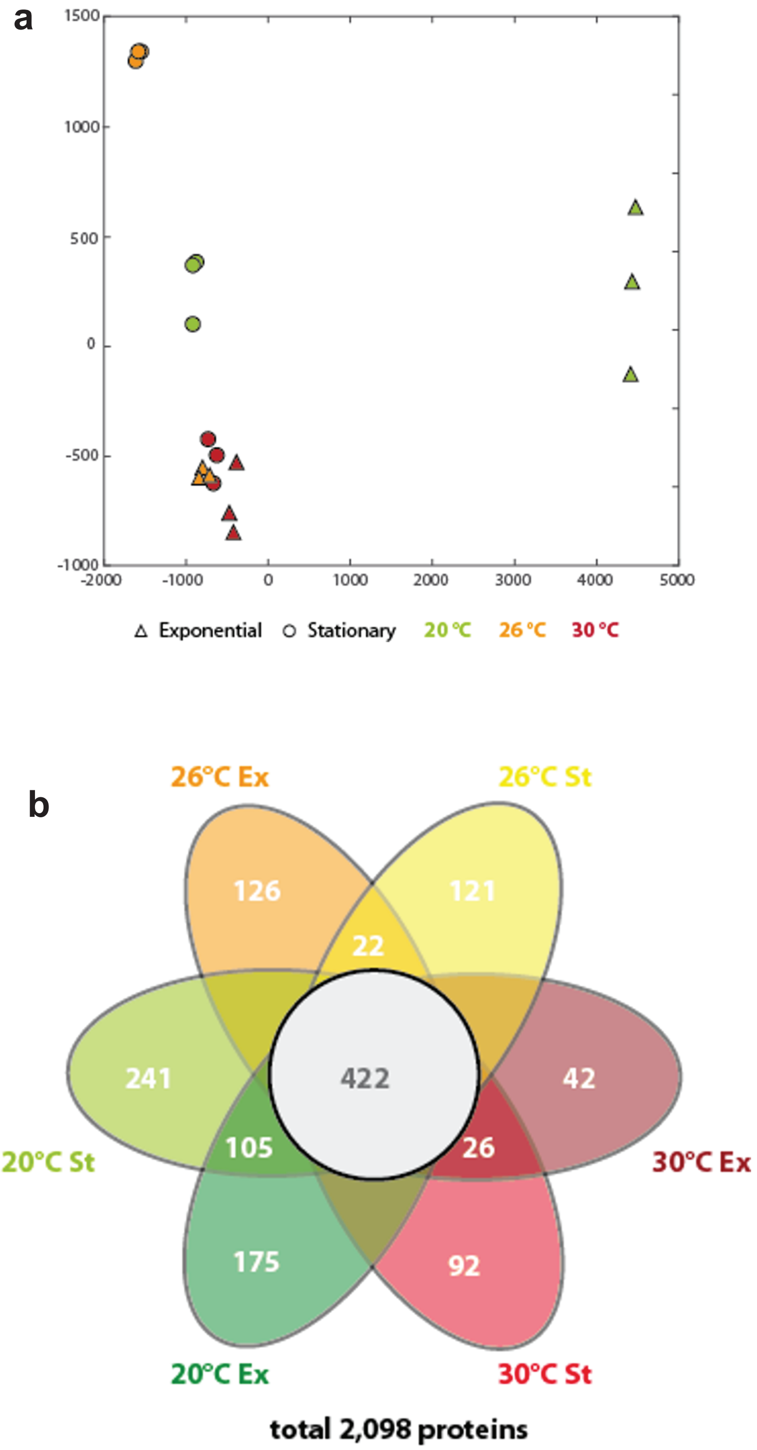


**Fig. S7.** The proteome landscape of *P. cordatum* heat-stress response, showing (a) NMDS visualization of individual samples based on peptide count data (max error = $1.24 \times 10^{-11}$), and (b) Venn diagram of number of proteins in distinct conditions.

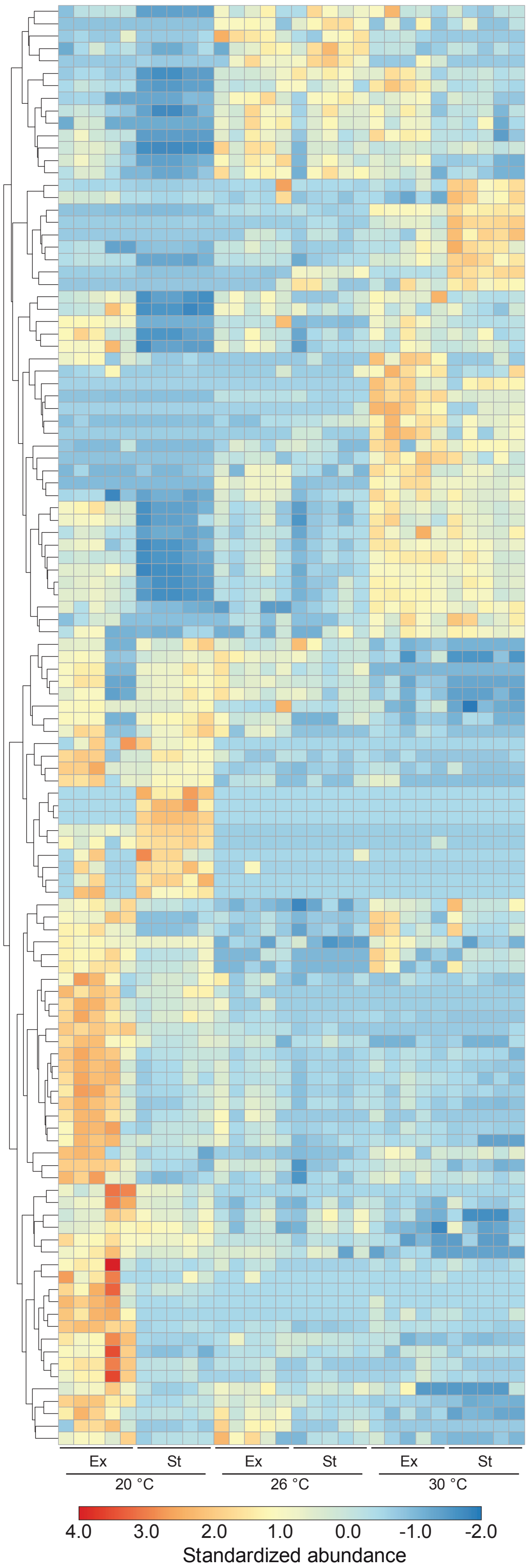


**Fig. S8.** Heatmap of all identified metabolites ($p < 0.001$) at 20°C, 26°C and 30°C in *P. cordatum*, for Ex and St phase. Five biological replicates and three technical replicates were run per experiment. Relative intensity values are shown, and data were normalized to internal standard and cell count.

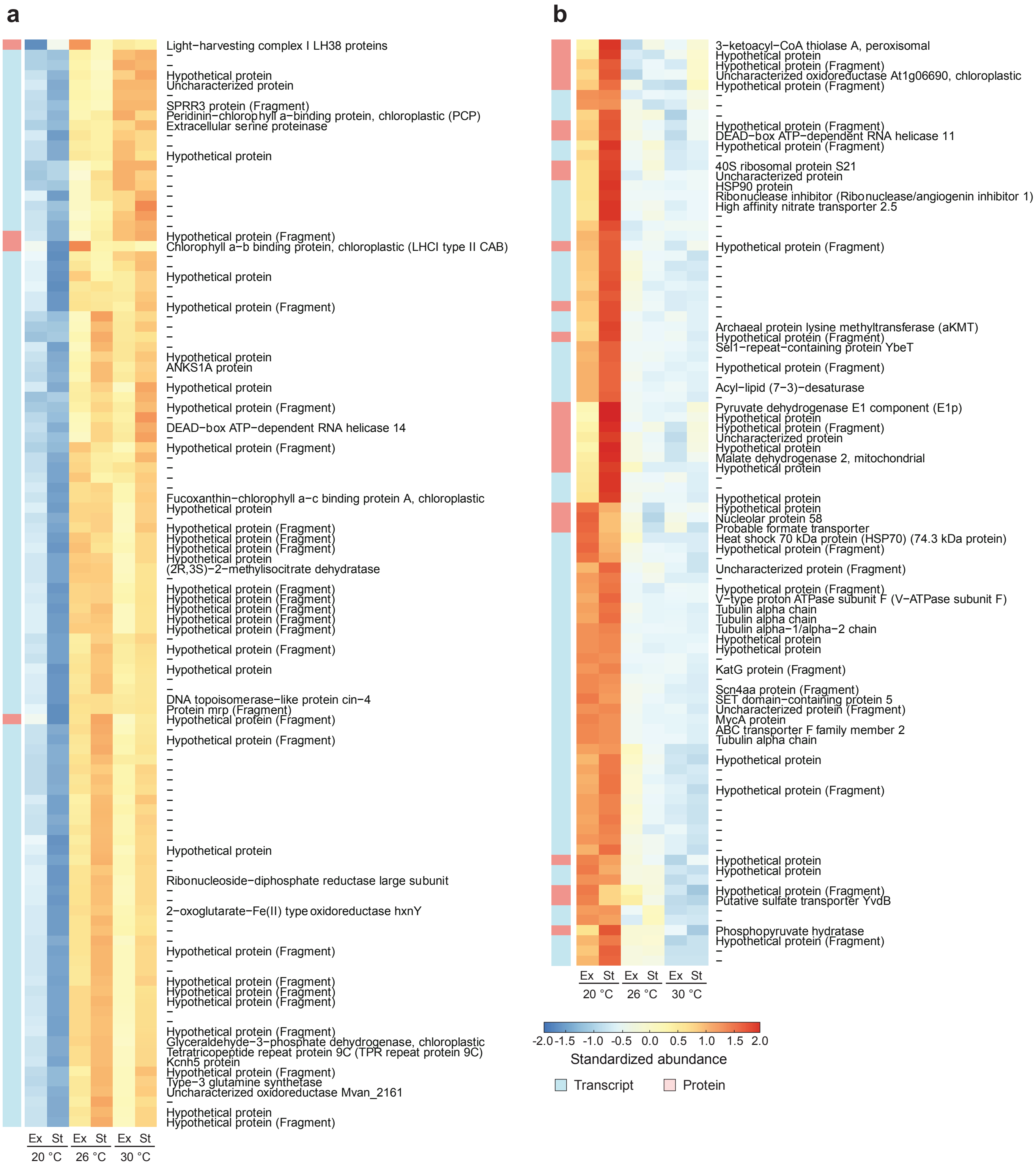


**Fig. S9.** Heatmap of expression levels for the inferred multi-omics signature that is indicative of a general heat stress response (component 1), shown for values scaled across rows with proteins and transcripts that are (a) up-regulated and (b) down-regulated during heat stress.

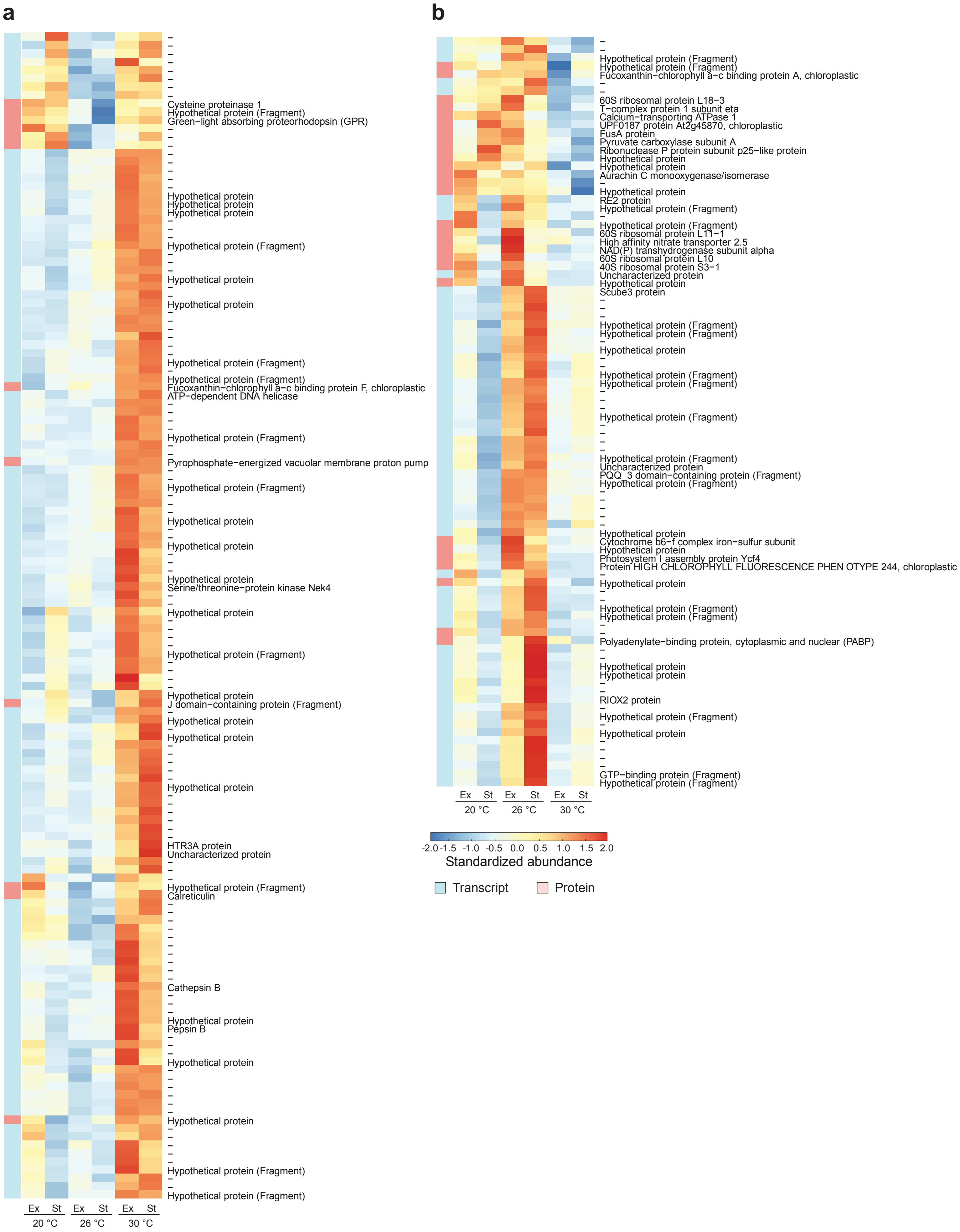


**Fig. S10.** Heatmap of expression levels for the inferred multi-omics signature that is indicative of a heat-stress response specific to 26°C and to 30°C (component 2), shown for values scaled across rows with proteins and transcripts that are (a) up-regulated and (b) down-regulated during heat stress.

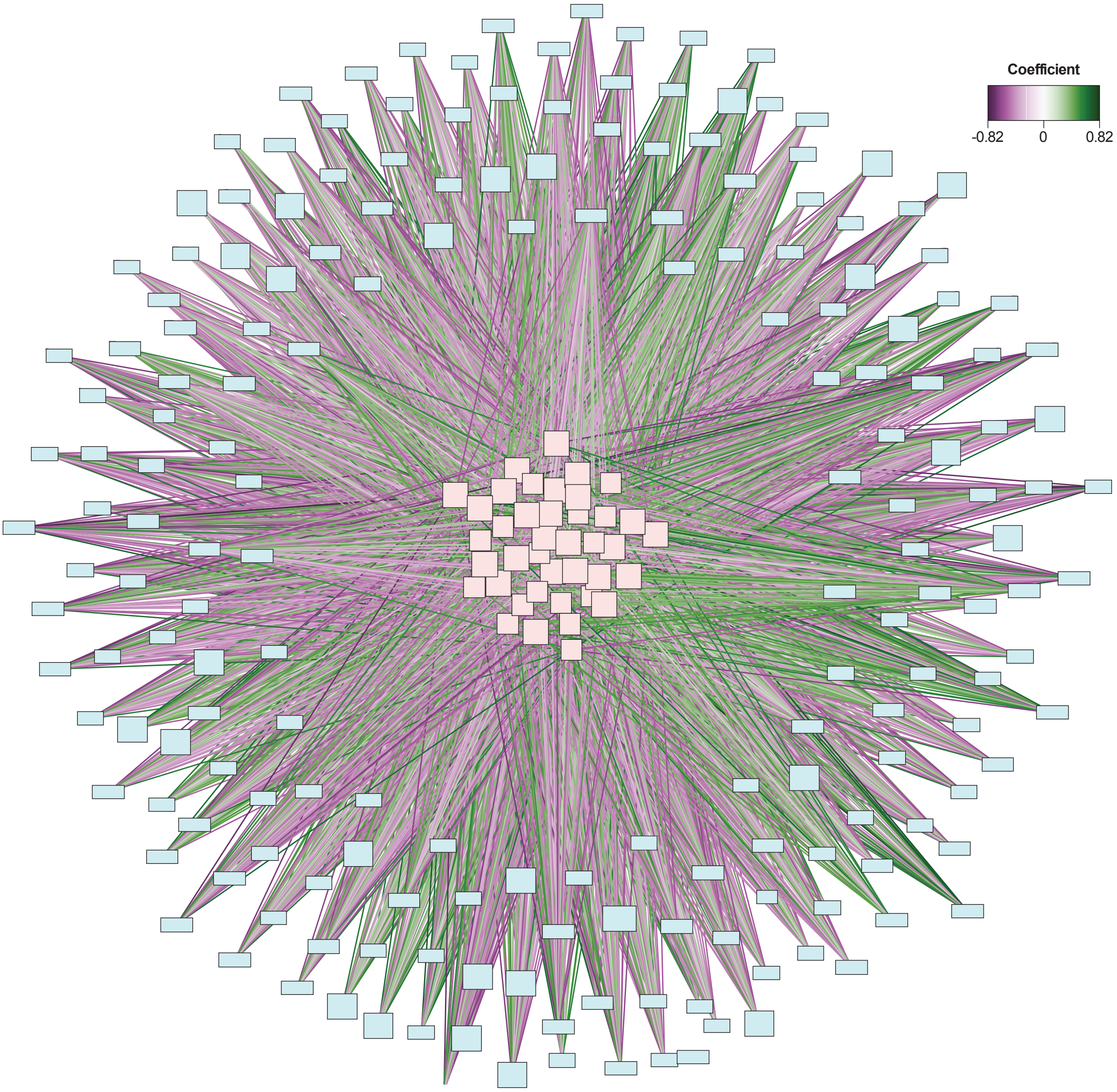


**Fig. S11.** Network visualization of multi-omics signature inferred from both proteins and transcripts that represent a heat-stress response specific to 26°C and to 30°C (component 2). Colors of the lines indicate the nature of the relationships between proteins and transcripts, with positive relationships in green and negative relationships in purple.

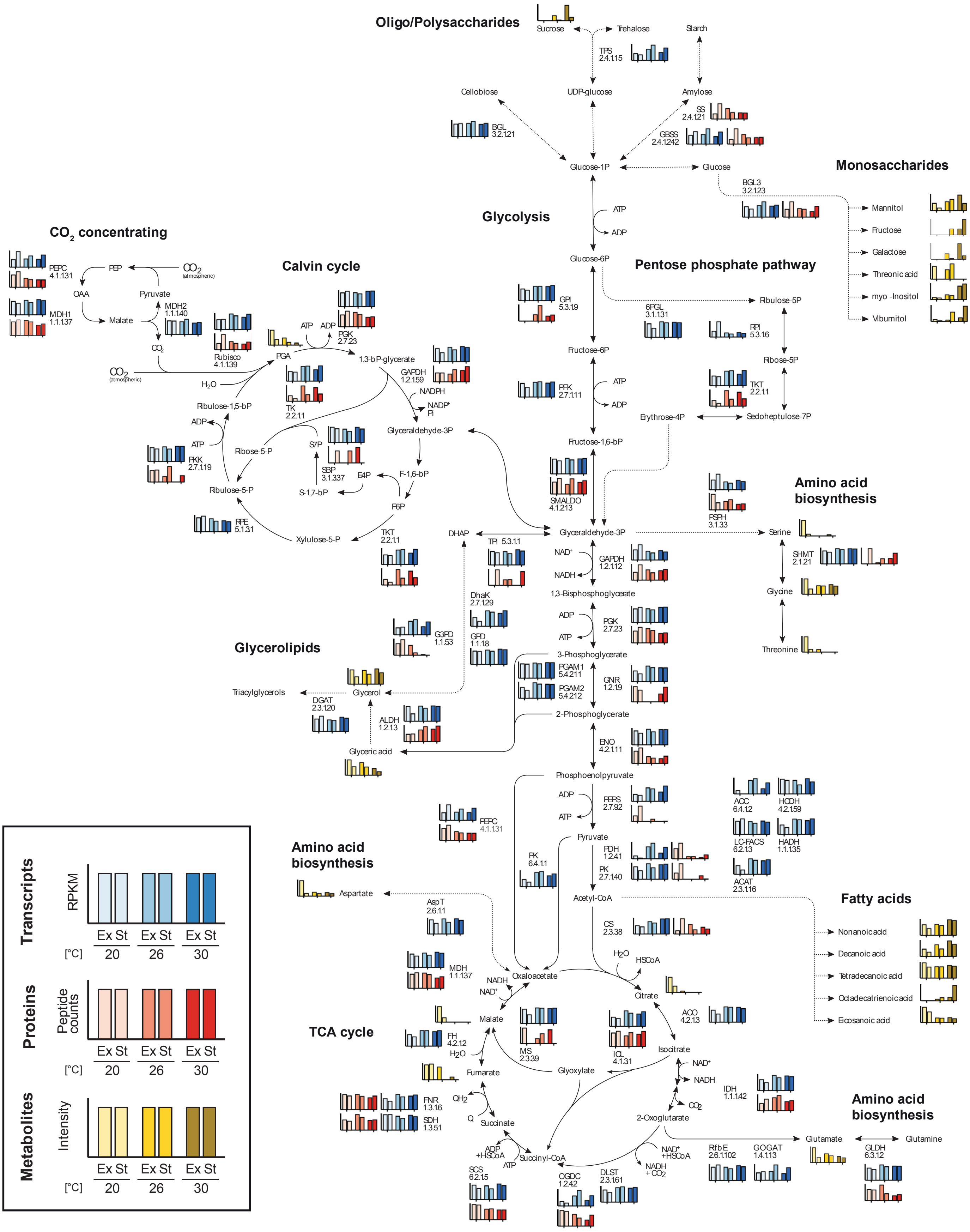


**Fig. S12.** Detailed reconstruction of the central carbon metabolism of *P. cordatum* and the concerted responses to heat stress response at transcript (blue), protein (red) and metabolite (yellow) levels.

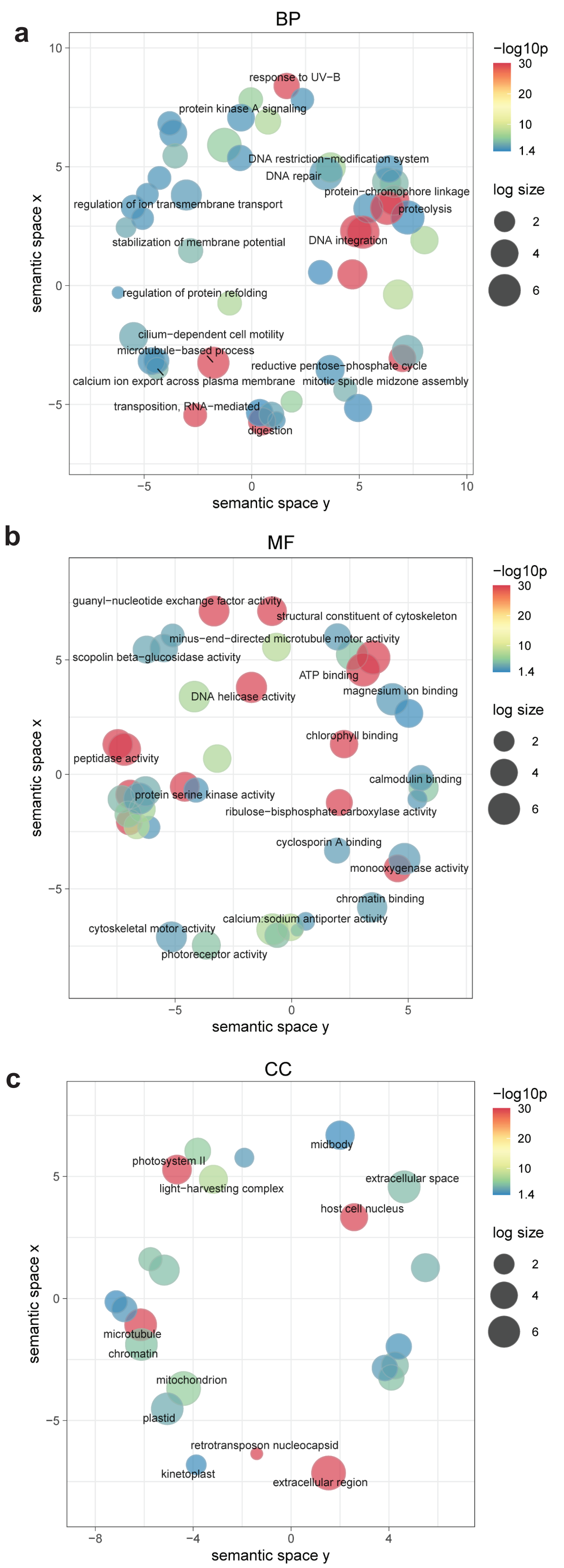


**Fig. S13.** Functional enrichment of dispersed gene copies based on GO terms in homologous sets of size 20 or greater (total of 13,529 genes), shown for (a) Biological Process, (b) Molecular Function, and (c) Cellular Component.

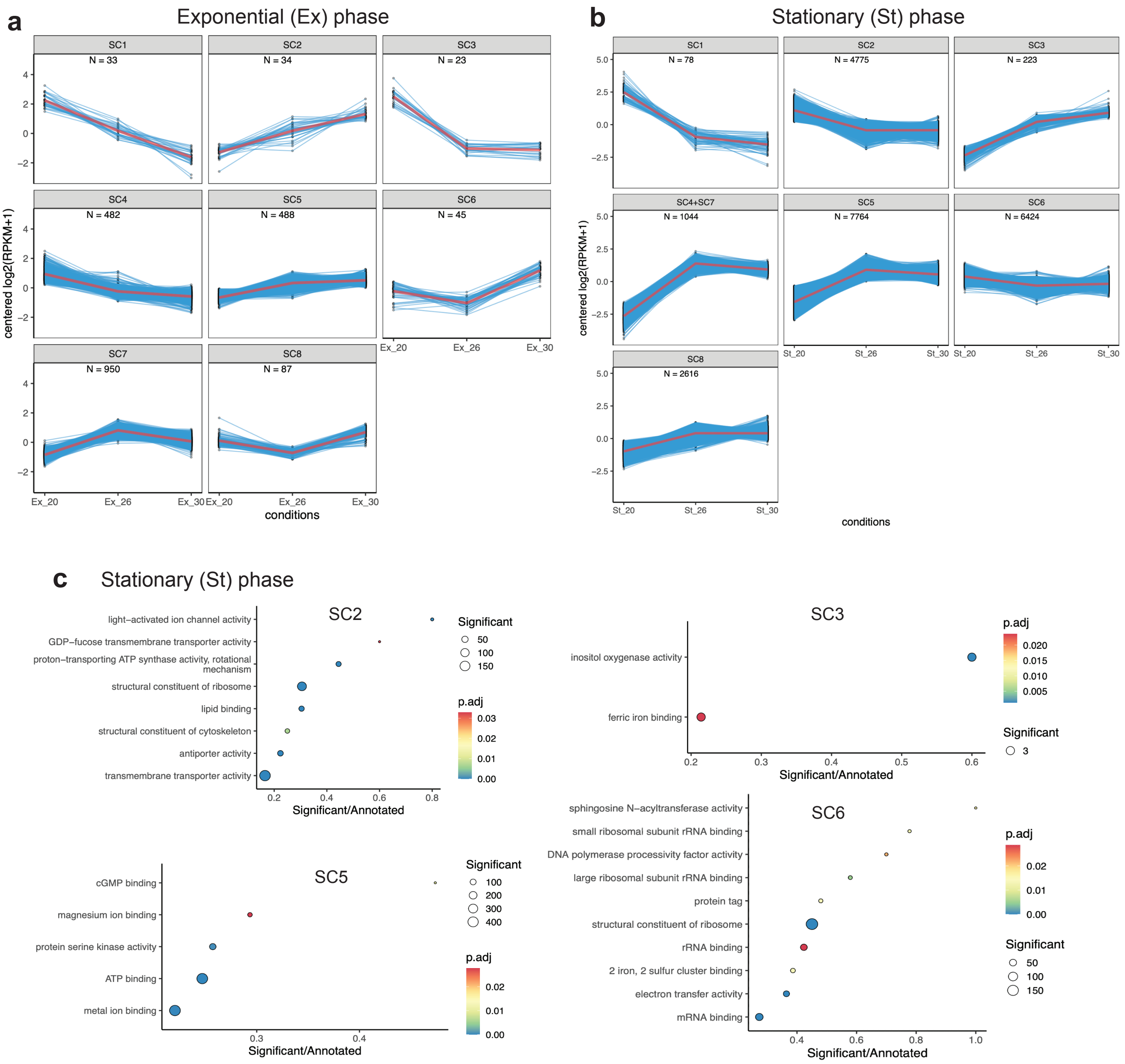


**Fig. S14.** Clusters of genes with similar expression patterns across different conditions in (a) exponential (Ex) and (b) stationary (St) phase, and (c) enriched gene functions in the Molecular Function GO terms in SC2, SC3, SC5, and SC6 in the St phase.

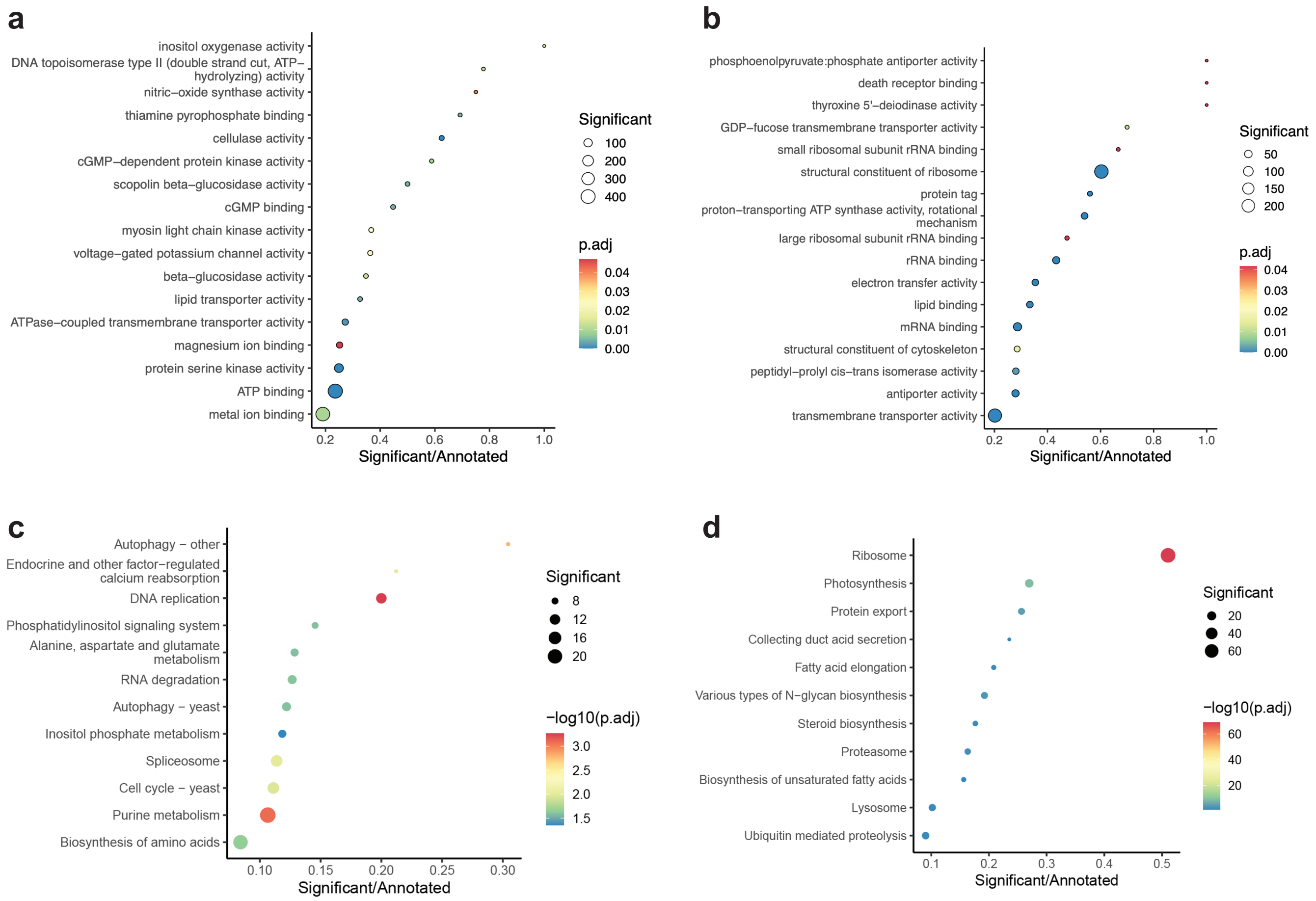


**Fig. S15.** Enriched functions in differentially regulated genes in 26°C versus 20°C in stationary phase, showing enriched Molecular Function GO terms in (a) up-regulated and (b) down-regulated genes, and the enriched KEGG pathways in (c) up-regulated and (d) down-regulated genes.

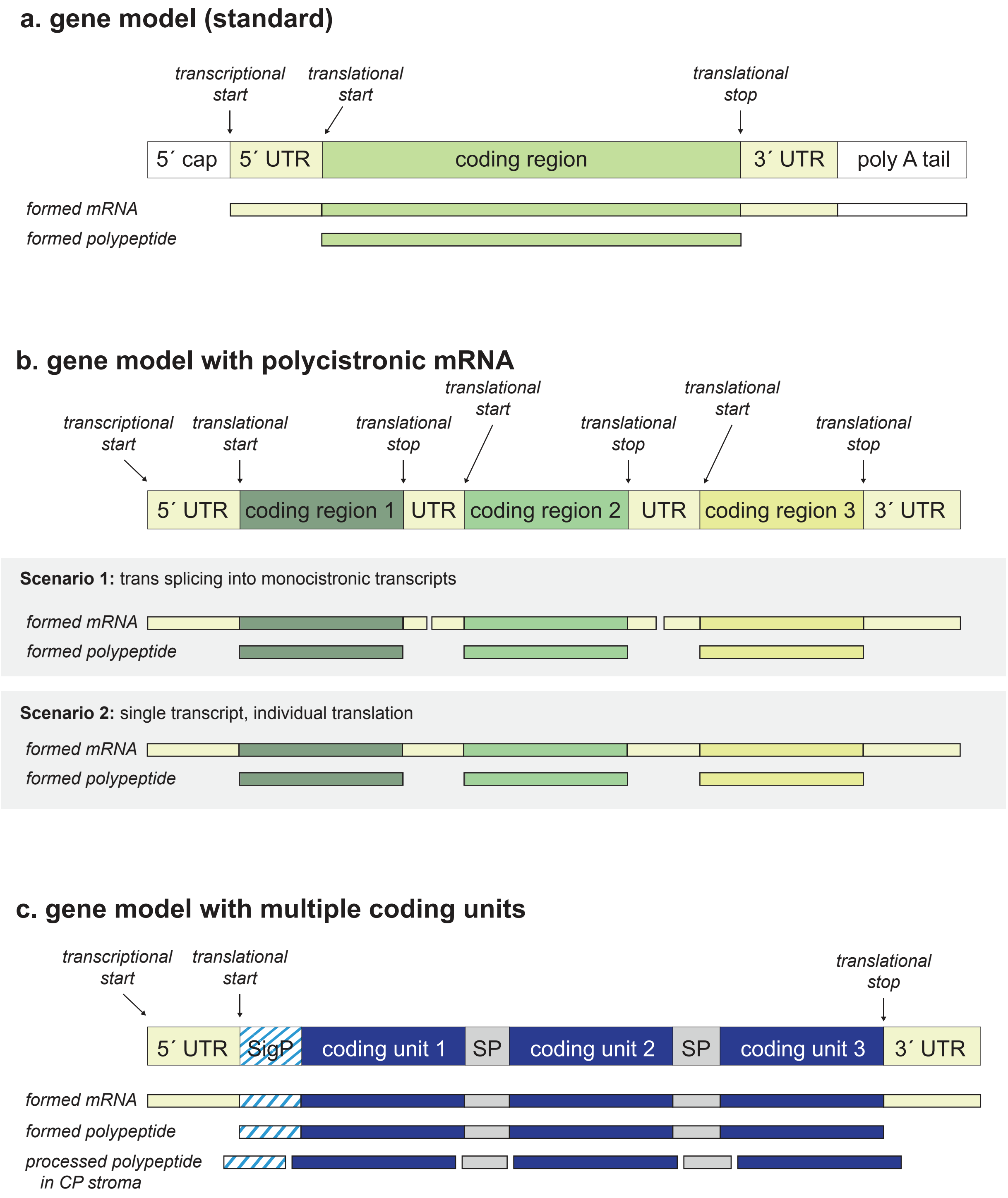


**Fig. S16.** Schematic diagram three distinct gene models identified in *P. cordatum*: (a) standard gene model, (b) gene model with polycistronic mRNA, and (c) gene model with multiple coding units.

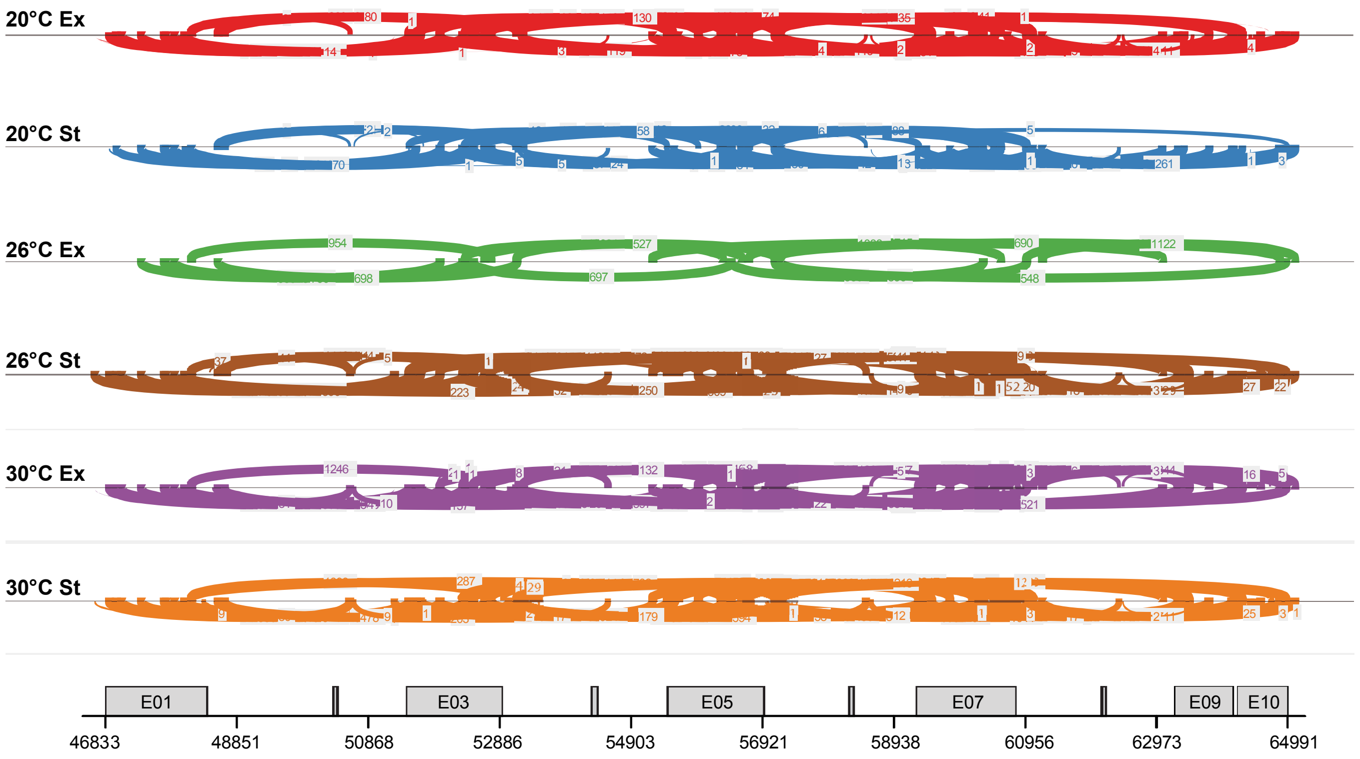


**Fig. S17.** Sashimi plot across the HSP70 locus displaying read coverage with lines between exons indicating connections supported by >500 alignments.

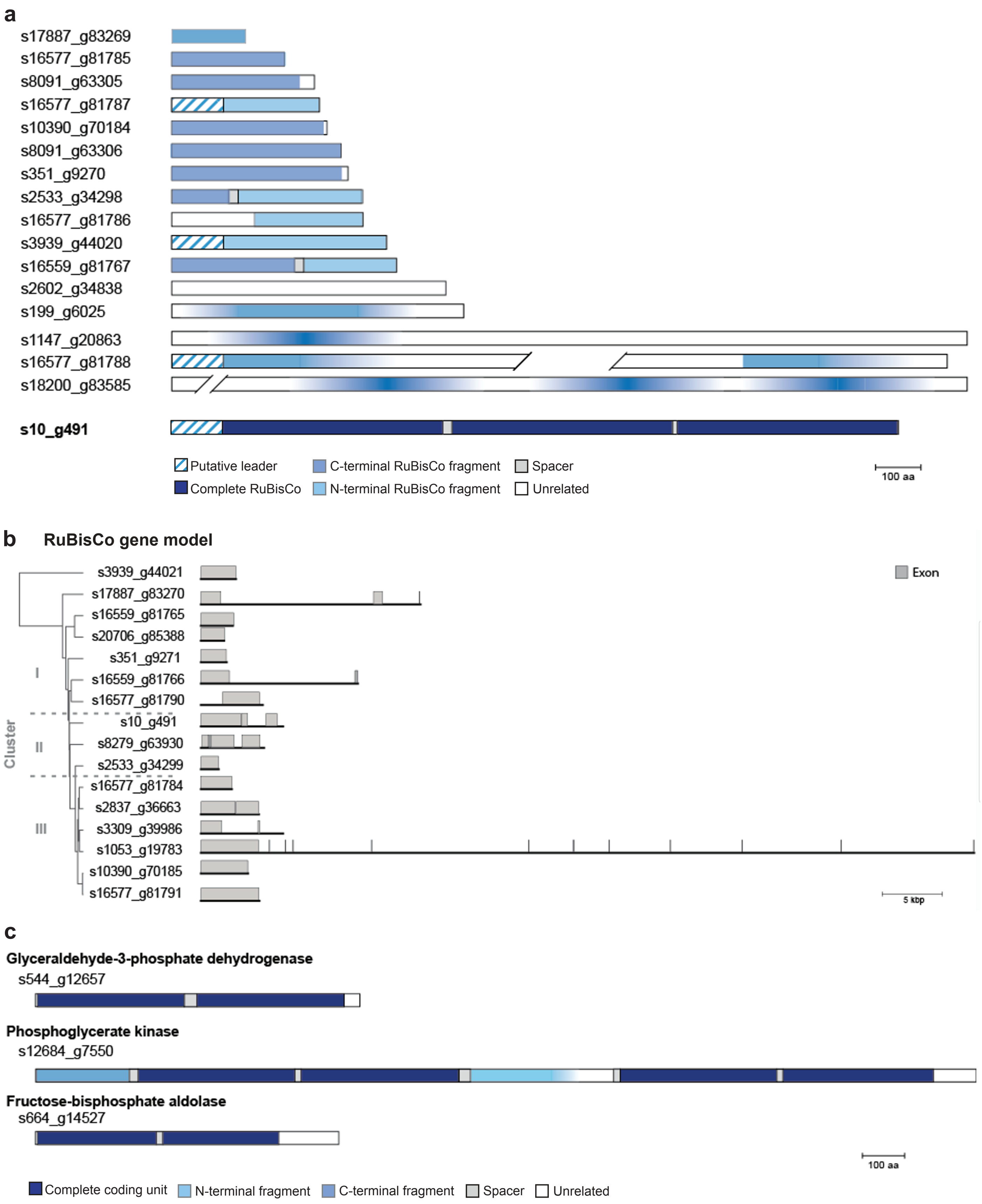


**Fig. S18.** Detailed information on gene models encoding multiple coding units (CUs), showing the scale model for (a) those containing only fragments of RuBisCo CU with s10_g491 as a reference that encodes 3 CUs; (b) RuBisCo gene models and the corresponding exons; and (c) those containing multiple CUs that encode protein functions related to central metabolism.

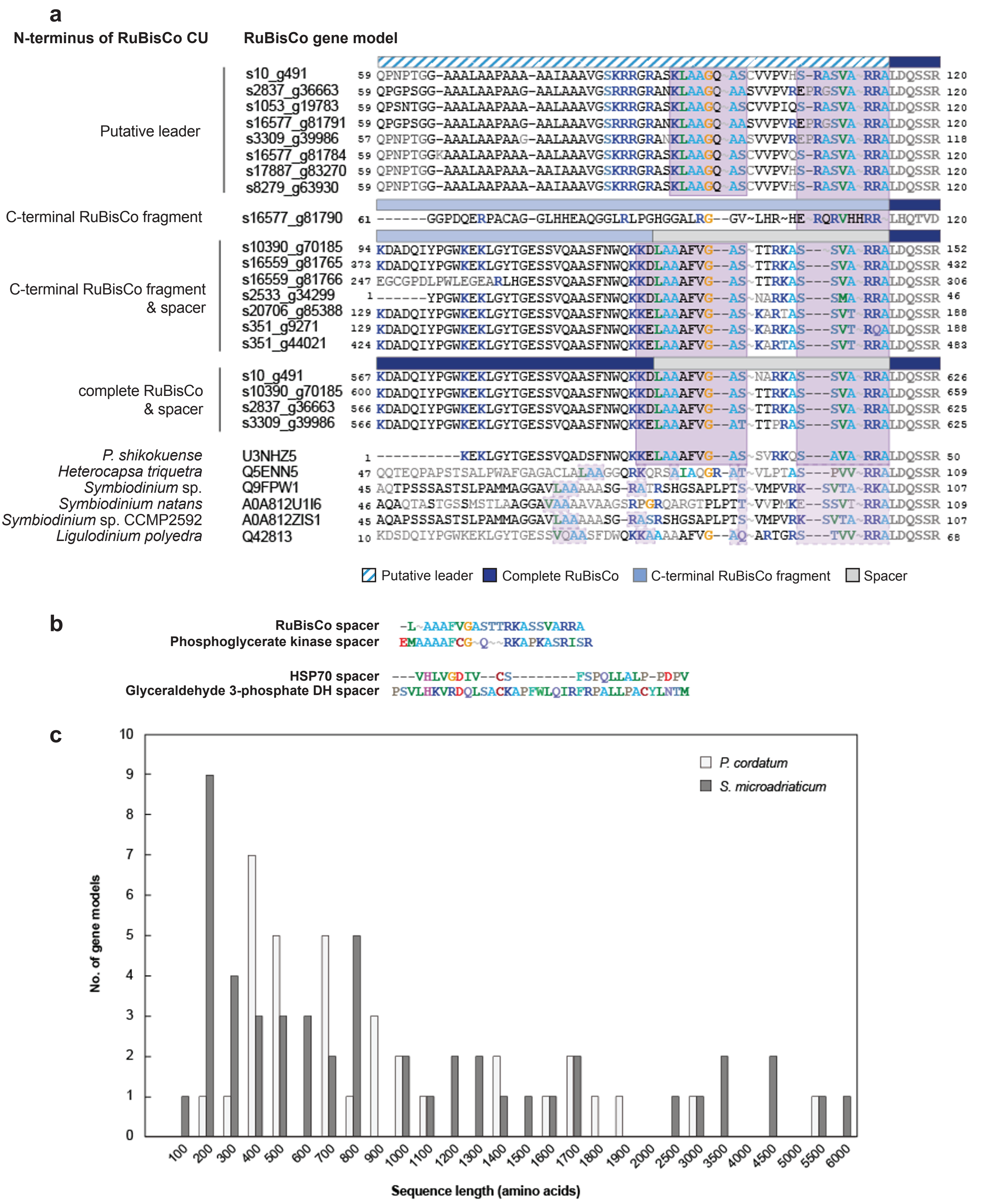


**Fig. S19.** Comparison of RuBisCo gene models of *P. cordatum* and other dinoflagellate species, showing the (a) alignment of the predicted leader sequences, N-terminal sequence of the first CU, and the spacer region between two complete CUs; (b) alignment of spacer regions of chloroplast-localized proteins (RuBisCo and phosphoglycerate kinase) and non-chloroplastic proteins (HSP70 and glyceraldehyde 3-phosphate dehydrogenase); and (c) histogram of Rubisco genes encoded in the genomes of *P. cordatum* and *S. microadriaticum* relative to protein-sequence length.

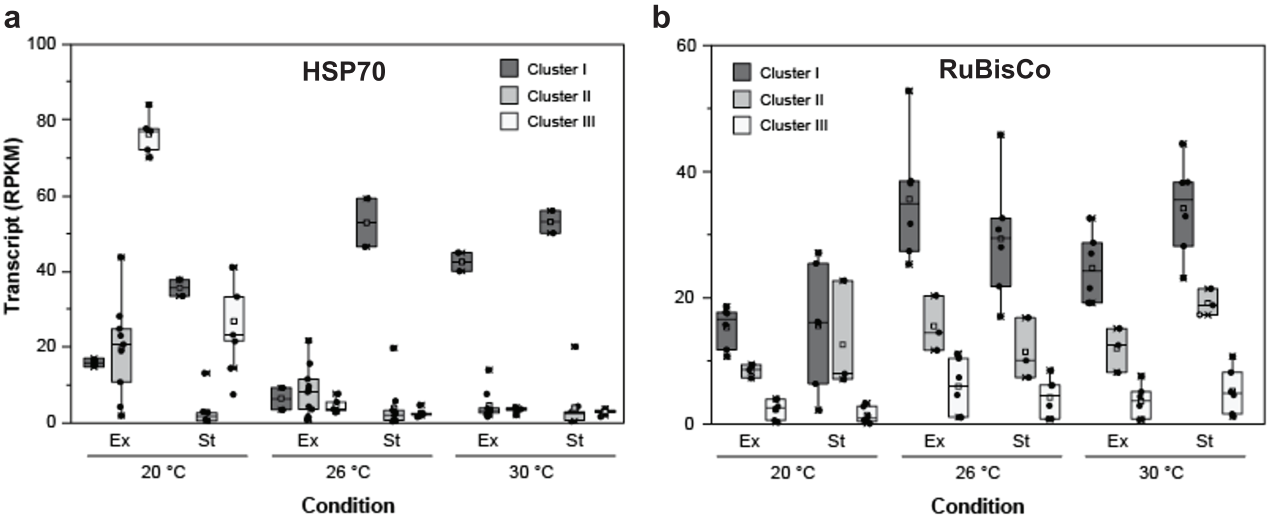


**Fig. S20.** Transcript profiles of (a) HSP70 and (b) RuBisCo gene models based on clustering in Figures 6a and 6c.
