# Supplementary material for: Multi-omics analysis reveals the molecular response to heat stress in a “red tide” dinoflagellate
Source: Genome Biol. 2023 Nov 23;24:265. doi: 10.1186/s13059-023-03107-4 (PMC10666404; doi:10.1186/s13059-023-03107-4)
Supplement: Supplementary file 5 — Additional file 5. Peer review history. [file 13059_2023_3107_MOESM5_ESM.docx]

**Review history**

**First round of review**

**Reviewer 1**

Why is the temperature stress used in the experiment related to global warming? Five or 10 degrees is larger than the 1.5 degrees rise expected by 2050. Is it perhaps more relevant to HAB formation? In any event, ten degrees seems a lot, many species do not survive such temperatures.

Why might genes change direction more frequently (peak of 30% at two orientation changes in 10 gene window)? Would this imply a different mechanism for gene expression or at least an increased number of transcriptional start sites compared to species where gene orientation changes less often?

Splice acceptor motif percentage seems a bit lower than other non-parasitic species. Is there a problem recognising the sites?

Fig S6. Are the numbers of proteins shown in the Venn diagram really only present in one condition? The data in Table S10 shows proteins that were detected only in one of 18 samples, are these among the 779 differentially expressed proteins? How many peptides were detected? Are all proteins all defined by 2 or more peptides each?

Table S7 The metabolome data shows products that would seem unlikely to be useful to living cells, such as phenol or benzene. Are they there only because they were identified?

Any correspondence between changed levels of transcripts and proteins? Figure 4b seems to suggest no, but it is unclear if it is the same sequences that are being analysed for RNA and protein.

The number of transcripts without an SL seems high. I wonder if it might be related to the larger number of gene orientation changes, as this might result in fewer transcripts where SL addition is no longer needed to carve out the 5' end of messages from a polycistronic primary transcript. The reference cited (#57) also noted few sequences without an SL (SL only called if there are less than 6 bases missing), is this the same criteria?

Fig 4a indicates increase in photosynthetic proteins at high temperature, but also shows a similar increase going to stationary phase at 20 degrees.

Line 213 makes a reference (#51) to Rubisco activity at higher temperature, but the protein in dinoflagellates is different (form II not form I).

The switch from RNA editing to alternative splicing is striking (Fig 5e). While the latter is very likely to produce a different protein sequence, is it possible to determine to what extent RNA editing will affect protein sequence? And is there any clue as to what might distinguish the two types of transcript?

Discussion of "Regulation of multi-protein-coding gene variants" is difficult to follow. Perhaps change line 291 to read "CUs are separated by spacer sequence that are collectively translated as a single protein with the spacer peptide subsequently removed". And on line 292 change sentence to read "This is different from polycistronic transcription in dinoflagellates, where multiple (different) genes are co-transcribed as a primary transcript which is subsequently processed into mature single coding unit transcripts by trans-splicing at the 5' end and polyadenylation at the 3' end of each coding unit".

Line 299 states 4.95% of transcripts contain dino SL, which implies only this percentage of transcripts will require trans splicing to produce the mature transcript. How can this be reconciled with the finding that most genes are unidirectionally encoded and presumably transcribed as polycistronic primary transcripts?

Figure 6a seems to show that hsp70 transcripts do not generally increase with temperature stress. Furthermore, only 26 degrees stationary phase appear to have increased protein abundance. Any idea why more hsp are not induced by heat? Figure 6b seems to have editing sites in introns. How is this detected? Figure 6c seems to show a disconnect between transcript abundance and protein abundance.

Line 313 indicates a comparison between multi-CU and single-CU genes. Where was the single CU gene?

Line 328 states there is no consensus sequence for the leader in plastid directed proteins. This was described by Nassoury et al 2003 J Cell Sci 116 2867-74 and Patron et al 2005 JMB 348 1015-24.

Line 346 reports finding promoter motifs. This seems very difficult to interpret in the context of polycistronic genes where the transcription start site may not be precisely known. Do the promoter motifs all include a TTTT box?

Line 354 Temperatures are unlikely to vary over the distances typically involved in diurnal vertical migration.

Line 581 indicates targeting peptides were identified using a variety of programs such as TargetP, signal and PSORT. How well do these predict a plastid location from the unusual tripartite leader sequences?

**Reviewer 2**

Multi-omics analysis reveals the molecular response to heat stress in a "red tide" dinoflagellate

It is a delight to see a globally distributed HAB-causing dinoflagellate sequenced. The manuscript delves into temperature stress response. Besides the genome, transcriptomes and limited scale of proteomes as well as metabolomes were created to enable an integrative interrogation of the interesting question—heat stress response.

I am highly interested in the finding that P. cordatum has high GC content. Because it is even higher than that in the coral symbionts Symbiodiniaceae, it is clearly not a signature of heat resistance signature. I think this perspective deserves a mention and some discussion.

Another interesting finding is the greater number of genes being transcriptionally dynamic than previously reported/believed. This is consistent with recent studies on transcriptional regulation of nutrient related genes in various species of dinoflagellates.

The complementary trend of mRNA editing and alternative splicing as mechanisms of post-transcriptional regulation is also interesting.

The manuscript is reasonably well organized, despite some roughness or disconnect of flow as shown in specific comments shown below. The results are clearly described and the major conclusions are well supported. The methods are generally sound. I recommend acceptance after some minor/moderate revision.

Specific comments are provided in the following.

1. Line 95: reference 31 hypothesizes the recycling of cDNA into the genome, not on the occurrence of trans-splicing.

2. Line 99: As far as I understand, P. cordatum is a globally distributed species, not an invasive species.

3. Lines 115-118: did you find the occurrence of introner elements in Polarella glacialis and A. gibbosum? If so, make a statement here; if not, this sentence is problematic.

4. Lines 126-130: comparing "unidirectionally encoded" with "dispersed duplicates", and inferring "duplications events occurred independently" is confusing.

5. Lines 134-139: The role of transposition in dinoflagellate genome evolution has been reported multiple times and relevant literature should be referred to here.

6. Lines 180-184 does not flow from the preceding sentence; it feels like jumping from one thing to the other, and cannot be connected by "For instance".

7. Line 330: Prorocentrum donghaiense has been renamed as Prorocentrum shikokuense.

8. Lines 351-352: compared with the symbiotic and parasitic dinoflagellates, does P. cordatum have fewer introns along with longer introns that you describe? The trend of number of introns would be interesting to show in the paper.

**Authors’ response to reviewers**

**Reviewer #1**

Why is the temperature stress used in the experiment related to global warming? Five or 10 degrees is larger than the 1.5 degrees rise expected by 2050. Is it perhaps more relevant to HAB formation? In any event, ten degrees seems a lot, many species do not survive such temperatures.

The 1.5°C-rise is a mean estimated value for the global oceans. However, the period when temperatures of 26-30°C would be encountered in certain depths and areas of the oceans is expected to be prolonged in future (Alexander et al. 2018, Elementa 6: 9). In this context, 30°C is ecologically relevant as it is already observed in summers during which dinoflagellates commonly bloom (Tester et al. 2020, 91: 101655). We have now clarified this in the Introduction and provided the background context for the experimental design in the Results. See also our response to Reviewer 2’s comment below.

# *L98–105:* “Among bloom-forming dinoflagellates, *Prorocentrum cordatum* (formerly *Prorocentrum minimum*) [32, 33] is an invasive, potentially toxic species that has expanded its habitat from the Caspian Sea where it was originally found to coastal oceans worldwide [34], including the temperate oceans in the North Atlantic where it is now regularly detected [35]. The tolerance of *P. cordatum* to a wide range of salinities and temperatures facilitates its increased bloom frequency [32, 33]. Along with the estimated 1.5°C rise in average sea temperatures, the period when temperatures range between 26°C and 30°C at certain depths and areas of the oceans is expected to be prolonged in future [36].”

*L147–150:* “To investigate the heat-stress response in *P. cordatum*, axenic cultures were grown in defined media at the optimal temperature (20°C) before they were exposed to either 26°C or 30°C (Fig. 3a); 30°C is already observed in summers during which dinoflagellates commonly form blooms [54], thus is ecologically relevant in this context.”

Why might genes change direction more frequently (peak of 30% at two orientation changes in 10 gene window)? Would this imply a different mechanism for gene expression or at least an increased number of transcriptional start sites compared to species where gene orientation changes less often?

The analysis of unidirectional coding of genes based on the arbitrarily defined ten-gene windows is a purely bioinformatics exercise following the approach used in earlier studies of dinoflagellate genomes (e.g., Lin et al. 2015 Science 350:691-4; Stephens et al. 2020 BMC Biology 18: 56). This approach is sensitive to contiguity of assembled genome sequences. Given the much larger genome size (4.75 Gbp) of P. cordatum compared to the other genomes, even with comparable sequence contiguity, we cannot dismiss the technical bias of the fragmented genome assembly in our observations.

Similarly, on the other extreme of the small genome of parasitic Amoebophrya ceratii (0.12 Gbp), we observed a spike at two orientation changes in Fig. S4. To systematically assess the correlation between gene-coding directionality and the regulatory mechanism of gene expression requires a tailored experimental design incorporating genome and transcriptome data from multiple species, which is beyond the scope of this study. In this instance, we take caution not to over-interpret the results.

Splice acceptor motif percentage seems a bit lower than other non-parasitic species. Is there a problem recognising the sites?

The approach we adopted to predict protein-coding genes in P. cordatum is similar to those used in the other studies of non-parasitic species detailed in Table 2, in which multiple lines of evidence from transcriptome and protein sequences were used to guide identification of intron/exon splice junctions, using tools that were customised to recognise non-canonical splice sites of dinoflagellates. As such, the lower percentage (79.7% in Table 2) of splice acceptor sites with AGG motif relative to other non- parasitic species (≥ 93%) is unlikely due to a technical issue. Moreover, the low percentage (56.5%) observed for the parasitic A. ceratii may be explained by the approach used for predicting protein- coding genes in the genome (John et al. 2019, Science Advances 5: eaav1110), for which the gene- prediction tools were not customised to explicitly recognise alternative splice sites of dinoflagellates.

As is standard practice with comparative genomic analysis based on de novo assemblies of variable sequence contiguity and quality, we take caution not to over-interpret metrics of genome assemblies and predicted genes.

Fig S6. Are the numbers of proteins shown in the Venn diagram really only present in one condition? The data in Table S10 shows proteins that were detected only in one of 18 samples, are these among the 779 differentially expressed proteins? How many peptides were detected? Are all proteins all defined by 2 or more peptides each?

Figure S6 (now Figure S7) shows the number of differentially abundant proteins in the distinct conditions. Table S10 shows the normalized spectral (peptide) counts for all detected proteins (of the corresponding gene models). Single peptide detections were considered if the same protein could be represented (i.e. identified) by two or more peptides in at least one replicate sample. This constraint is specifically designed to avoid artificial exclusion of small, hydrophobic proteins that give rise to very few detectable peptides. In total, ~19,000 different peptides were detected from ~880,000 peptide spectra. We have now clarified this in the text.

*L165–168:* “We found 779 proteins with significantly changed abundance at 26°C or 30°C compared to 20°C, with functions related to photosynthesis, energy generation, and central metabolism (Fig. 3d, Additional File 2: Fig. S7; all detected proteins shown in Additional File 3: Table S10).”

*L813–821:* “For data analysis, spectral counts for the identified proteins were determined per sample and experimental approach (i.e. membrane protein-enriched fraction, 1D and 2D separated shotgun). Per sample replicate, only the maximum spectral count of one of the three approaches per protein was considered for subsequent analyses, and only proteins detected in at least two replicate samples were included. Custom Matlab (version 2021a; MathWorks, Natick, MA, USA) code was used for data analyses. Following quantile normalization, ANOVA *p*-value and fold-change were calculated for each protein. Proteins that exhibit significant changes (*p* ≤ 0.05) in distinct conditions were considered to be differentially expressed.”

Table S7 The metabolome data shows products that would seem unlikely to be useful to living cells, such as phenol or benzene. Are they there only because they were identified?

The metabolome data were presented in Table S11. These data represent all detected metabolites as measured by GC-MS. Some of the detected compounds (such as phenol or benzene highlighted by the Reviewer) have no clear physiological role in P. cordatum.

Any correspondence between changed levels of transcripts and proteins? Figure 4b seems to suggest no, but it is unclear if it is the same sequences that are being analysed for RNA and protein.

Compared to transcripts, proteins (and metabolites) exhibit differential rates of synthesis and turnover that reflect regulatory constraints. As such, the expression levels of transcripts and proteins are not coherently linked in a one-to-one relationship, e.g. a protein may still be present in the sample at the time of extraction, long after the transcript is expressed and translated. The individual cause for incoherence in these transcript-protein profiles cannot be discerned simply using transcriptome and proteome data generated from the same sample. However, proteome and metabolome data are powerful for validating phenotypic outcomes, and they provide evidence central to our interpretation of molecular response to heat stress.

Our analysis focused on both detected proteins and their corresponding gene models (Table S13). As expected in such an analysis, a greater diversity of transcript isoforms was identified for the gene models compared to protein isoforms. Some detected protein sequences are highly similar (with some sharing 100% identity), therefore their assignment to the specific gene duplicates (i.e., functionally very similar gene models) is not clearly distinguishable. We clarified this in the text when presenting the results of RuBisCo analysis.

# *L356–357:* “Associating expressed peptides to distinct CUs is not straightforward due to the high pairwise protein sequence identity (>95%).”

The number of transcripts without an SL seems high. I wonder if it might be related to the larger number of gene orientation changes, as this might result in fewer transcripts where SL addition is no longer needed to carve out the 5' end of messages from a polycistronic primary transcript. The reference cited (#57) also noted few sequences without an SL (SL only called if there are less than 6 bases missing), is this the same criteria?

We adopted the same approach as described in Stephens et al. (2020, BMC Biology 18: 56) to identify dinoSL sequences in the P. cordatum transcripts. The fewer dinoSL sequences we identified in P. cordatum are likely explained by the RNA-Seq transcriptome data generated for this study, which is known to yield fragmented sequences that do not represent full-length or near-full-length transcripts. In Stephens et al. (2020) and others (e.g., Gonzalez-Pech et al. 2021, BMC Biology 19: 73) in which transcriptome data were generated using PacBio Iso-Seq technology (designed to sequence eukaryotic transcripts to full-length), the recovery of dinoSL in those sequences is expected to be greater. For this reason, ours results here are inappropriate for investigating the correlation between the presence of dinoSL sequences and gene-orientation changes.

Fig 4a indicates increase in photosynthetic proteins at high temperature, but also shows a similar increase going to stationary phase at 20 degrees.

Yes, Fig 4a shows that heat stress and stationary phase response both have global effects on gene expression/protein formation. We have clarified this in the text.

# *L197–203:* “A global visualization of the relevant expressed transcripts (753) and proteins

(278) revealed differential expression at elevated temperatures, with a marked increase in photosynthesis proteins but a decreased abundance of proteins related to central metabolism and oxidative phosphorylation (Fig. 4a). This result is supported by reduction in the accumulation of 38 metabolites associated with central metabolism at elevated temperatures. We also observed a similar pattern of elevated expression in transcripts and proteins coding for photosynthetic functions in the St phase relative to Ex phase at 20°C.”

Line 213 makes a reference (#51) to Rubisco activity at higher temperature, but the protein in dinoflagellates is different (form II not form I).

We expect the observation for form I RuBisCo also applies to form II RuBisCo in the dinoflagellates. We have clarified this in the text.

# *L220-223:* “The generally unchanged profile of CO2-fixing ribulose 1,5-bisphosphate carboxylase (RuBisCo; Calvin cycle) may be misleading in this context; because activity of form I RuBisCo is known to decrease at higher temperatures [56], we expect similar trend for form II RuBisCo in dinoflagellates as well.”

The switch from RNA editing to alternative splicing is striking (Fig 5e). While the latter is very likely to produce a different protein sequence, is it possible to determine to what extent RNA editing will affect protein sequence? And is there any clue as to what might distinguish the two types of transcript?

Depending on the locations of RNA editing (e.g. in promoter, exon, or intron regions), the nature of the editing (i.e. synonymous versus non-synonymous substitutions), and effect on the protein sequence (e.g. including silent, missense, and nonsense mutations), the consequences of RNA editing are highly variable. Although linking RNA edits within protein-coding regions to their effects on proteins may be more straightforward, synonymous RNA edits could also affect integrity and stability of the RNA secondary structure, which are known to have serious biological ramifications, e.g., synonymous variants linked to disease in humans.

Our results provide a comprehensive overview of the interplay between RNA editing and alternative splicing in regulating gene expression, for the first time in a bloom-forming dinoflagellate species in response to heat stress and in distinct growth conditions. A systematic investigation of the impact of different RNA edit types on protein sequences requires a tailored experimental design including functional validation, which is beyond the scope of this study. We cautiously refrain from over- interpreting our results in this regard.

Since our original submission in December 2022, we noticed some inconsistencies in the results presented in Figure 5e. We have now **revised Figure 5e**, and the associated results in the new **Additional File 4: Data S1** (due to the large file size). These results do not affect our earlier interpretations and the conclusions drawn, and we have updated the text accordingly.

*L273-279:* “We found evidence of post-transcriptional regulation in 11,350 genes (Fig. 5e): 9,098 genes (involving 45,180 sites) with differential RNA editing, 1,820 genes (involving 6,243 exons) with DEU (Additional File 3: Table S22), and 432 genes (involving 2,367 sites and 1,177 exons) with both modes of regulation. The number of mRNA edits were similar under all conditions, but the type of edit and overlap between edited sites was condition- specific (Fig. 5e, Additional File 4: Data S1).

*L280–287:* “Interestingly, the relatively few (432) genes with both RNA editing and DEU in

*P. cordatum* suggests that post-transcriptional regulation of a gene tends to involve only one of these two modes: the editing of RNA was identified predominantly in genes for which no DEU was identified, and *vice versa* (Fig. 5e); singleton genes showed a greater extent of DEU in contrast to the dispersed duplicates that exhibited a greater extent of RNA editing. The 432 genes displaying both modes of regulation encode key photosynthetic and stress-related functions, such as chloroplastic ATP synthase subunits (β and ε subunits), ferredoxin, photosystem I reaction center subunit XI, and heat shock 70 kDa protein.”

Discussion of "Regulation of multi-protein-coding gene variants" is difficult to follow. Perhaps change line 291 to read "CUs are separated by spacer sequence that are collectively translated as a single protein with the spacer peptide subsequently removed". And on line 292 change sentence to read "This is different from polycistronic transcription in dinoflagellates, where multiple (different) genes are co- transcribed as a primary transcript which is subsequently processed into mature single coding unit transcripts by trans-splicing at the 5' end and polyadenylation at the 3' end of each coding unit".

We have reworded these sentences and cited the specific panels of Figure S16 for clarity.

# *L301–307:* “These CUs are separated by spacer sequences that are collectively transcribed as a single transcript that was then translated as a single protein sequence, after which the spacer peptides were removed (Additional File 2: Fig. S16c). This is slightly different from the usual polycistronic transcription in dinoflagellates (Additional File 2: Fig. S16b), whereby multiple (different) genes are co-transcribed as a primary transcript that is processed subsequently, each gene via *trans*-splicing at the 5′-end and polyadenylation at the 3′-end [30], into individual mature transcripts.”

Line 299 states 4.95% of transcripts contain dino SL, which implies only this percentage of transcripts will require trans splicing to produce the mature transcript. How can this be reconciled with the finding that most genes are unidirectionally encoded and presumably transcribed as polycistronic primary transcripts?

Per our response above related to the analysis of dinoSL sequences (in page 3), we refrain from over- interpret our results in this regard. We have made this point explicit in the text.

# *L312–317:* “We found 4.95% of *P. cordatum* transcripts to contain dinoSL (Additional File 3: Table S23) involving 17,214 (20.1% of 85,849) gene models, including those encoding HSP70, lending further support to this idea (Additional File 1: Supplementary Note). However, due to the technical limitations of identifying dinoSL in transcriptome data generated from RNA-Seq in this study (for which the assembled transcripts are likely fragmented), we took caution not to over-interpret these results.”

Figure 6a seems to show that hsp70 transcripts do not generally increase with temperature stress. Furthermore, only 26 degrees stationary phase appear to have increased protein abundance. Any idea why more hsp are not induced by heat? Figure 6b seems to have editing sites in introns. How is this detected? Figure 6c seems to show a disconnect between transcript abundance and protein abundance.

Per our response above related to the incoherence of transcript-protein profiles (in page 3), proteins and metabolites exhibit differential rates of synthesis and turnover that reflect regulatory constraints. Recovery of these proteins is not coherently linked in a one-to-one relationship with expression of the corresponding transcript, as we observed in Figures 6a and 6c.

In the case of HSP70, the decrease in transcript abundance during the stationary phase may reflect their active translation into proteins in response to heat stress, against the backdrop of HSP70 protein amplified by expression of the multi-CU transcripts, and proteins have markedly longer half-life than the transcripts.

Our approach for identifying RNA editing, using the established tool of JACUSA (Piechotta et al. 2022, Genome Biology 23: 115), incorporates both genome and transcriptome (RNA-Seq) data to identify significant RNA edits in the transcriptome against the background of single-nucleotide variants in the genome sequences; the latter may present false positives. The identified RNA edits were mapped onto the annotated genome features, including intronic regions. Using this approach, RNA edits can be recovered in intronic regions because RNA-Seq data are expected to include non-coding RNAs and introns of pre-mRNAs that are known to affect mRNA stability and subsequent patterns of alternative splicing. We have cited the paper of JACUSA in Methods, and we refer the readers to the paper for more detail.

*L628–630:* “For each condition, mapping of RNA-Seq reads was compared with the mapped genome-sequence reads using JACUSA v2 (*call-2 -F 1024 -P2 RF-FIRSTSTRAND -s -a D,Y,H:condition=1*) [111].”

Line 313 indicates a comparison between multi-CU and single-CU genes. Where was the single CU gene?

The presence of single- and multi-CU gene models encoding functionally similar proteins (i.e., paralogs) is a trait of dinoflagellates. Here, we focused on the expression and structural pattern of the multi-CU gene models relative to the single-CU counterpart(s), and selected RuBisCo as a

physiologically important example. We did not exclude single-CU gene models from this analysis. We have now clarified this in the text.

# *L328–330:* “To assess expression heterogeneity in response to heat stress among paralogs occurring as single-CU and multi-CU gene models, we selected RuBisCo for its central physiological function.”

Line 328 states there is no consensus sequence for the leader in plastid directed proteins. This was described by Nassoury et al 2003 J Cell Sci 116 2867-74 and Patron et al 2005 JMB 348 1015-24.

We are grateful for this comment. We have revised the text and cited the relevant references accordingly.

# *L340–355:* “Transport of RuBisCo to the plastid is facilitated by transit peptides including a leader sequence encoded upstream of the gene; these sequences are highly diverged among photoautotrophs [64, 65]. In dinoflagellates, an elevated serine/threonine and alanine content, and a potential motif of FVAP close to the N-terminus was predicted [66, 67]. We found, in the upstream region of eight gene models (Fig. 6d), the predicted leader sequence from the closely related *Prorocentrum shikokuense* [59]. We identified two conserved sequence motifs in the leader sequence, the N-terminal region of proteins that lacked a leader sequence, and the spacer region between two CUs (Fig. 6e). The two sequence motifs are moderately hydrophobic and rich in basic amino acid residues (*i.e.* arginine or lysine) as expected for plastid transit peptides [65]. We also observed a high content of serine and alanine, and an FVGA motif in the spacer regions (upstream of the N-terminus; Fig. 6d). The conserved sequence regions indicate that proteins coded by these single- and multi-CU genes in *P. cordatum* can be transported into the plastid. Highly similar sequence motifs present in *P. shikokuense* and other dinoflagellates support this hypothesis, and these motifs may be taxon- specific, e.g. FVGA is conserved in *Prorocentrum*, but not in the analyzed RuBisCo proteins of *Heterocapsa*, *Symbiodinium*, and *Ligulodinium* taxa (Additional File 2: Fig. S19).”

Line 346 reports finding promoter motifs. This seems very difficult to interpret in the context of polycistronic genes where the transcription start site may not be precisely known. Do the promoter motifs all include a TTTT box?

For each of the 32 P. cordatum gene models coding for RuBisCo, we extracted its upstream sequence region (up to 500 bp or up to the exon boundary of another gene upstream, whichever is shorter). Using this approach, 28 upstream sequence regions (>8 bp) were used for identification of promoter sequences based on conserved motifs among them using a machine-learning approach, MEME. This approach yielded three distinct motifs, which we used as a query to search against the curated motif repositories of transcription factor-binding sites (available from https://tfbsdb.systemsbiology.net/): JASPAR, SELEX, and UniPROBE. Using this approach, we found one motif that is potentially associated with a zinc finger-binding motif. However, given the very limited database resources that were derived from vertebrate and plant model systems (evolutionarily very distantly related to dinoflagellates), functional implications of these identified motifs remain unclear. We did not recover clear TTTT motifs in these sequences.

The paucity of transcription factors in dinoflagellates is well established (e.g. Beauchemin et al., 2012, Bayer et al., 2012, Roy and Morse et al. 2013). More recently, some putative transcription factors in dinoflagellates were shown to preferentially bind to RNA molecules in contrast to the canonical DNA- binding mechanism expected in transcription factors (Zaheri and Morse, 2021, BMC Molecular and Cell Biology, 22:27). Under these circumstances, we agree with the Reviewer that the interpretation of putative promoter (or transcription factor-binding motifs) here, based on very limited supporting evidence, is difficult to interpret, particularly in the context of polycistronic transcription. The result presented in this sentence, focusing on RuBisCo genes, does not add significant value to our results focusing on alternative exon usage and RNA editing from the overall P. cordatum gene models.

A comprehensive analysis of non-coding RNAs and their putative gene targets from broadly sampled taxa would provide biological insights into the molecular regulatory mechanism that underpin gene expression in dinoflagellates; this is beyond the scope of this study. For these reasons, we decided to delete this sentence from the main text.

Line 354 Temperatures are unlikely to vary over the distances typically involved in diurnal vertical migration.

Diurnal vertical migration of plankton in the ocean can cover many hundreds of metres, thus encountering large gradients of temperature, oxygen, and nitrate (Aumont et al. 2018, Global Biogeochemical Cycles, 32: 1622-43). Observations on the distances that are migrated vertically by dinoflagellates are rare. Earlier studies have demonstrated migration of P. minimum (=P. cordatum) across an artificially established halocline (Olsson and Granéli 1991, Journal of Planktonic Research 13: 1313-24), and Prorocentrum micans across temperature gradients of 7°C (Kamykowski et al. 1981, Marine Biology 62: 57-64). We have now clarified this point in the main text.

# *L370–376:* “Habitats of *P. cordatum* are known to have rapidly fluctuating temperatures, such as diurnal vertical migration or in spring blooms and red tides. Diurnal vertical migration of plankton in the ocean is known to cover many hundreds of meters, encompassing large gradients of temperature, oxygen, and nitrate [70]. Although distances of vertical migration of dinoflagellates are little known, earlier studies have demonstrated migration of *P. minimum* (=*P. cordatum*) across an artificially established halocline [71] and *Prorocentrum micans* across temperature gradients of 7°C [72].”

Line 581 indicates targeting peptides were identified using a variety of programs such as TargetP, signal and PSORT. How well do these predict a plastid location from the unusual tripartite leader sequences?

Leader sequences are not explicitly incorporated in the algorithmic design of the tools designed to identify signal peptides for plastid-targeting proteins. However, these tools are commonly based on profiles of sequence alignments in a hidden Markov model approach to elucidate conserved motifs. If the leader sequence is not globally conserved (or only conserved restrictively to a small subset of taxa, if included), the signal of such motifs would not have strong predictive power. We also recognise the distinct signal peptides (test sets) used in the different tools for predicting signal peptides, which would affect the prediction outcomes. For this reason, we employed multiple methods in predicting transit peptides, in which we inferred these peptides based on the consensus of results from the five methods (see Methods, L606–609).

**Reviewer #2**

It is a delight to see a globally distributed HAB-causing dinoflagellate sequenced. The manuscript delves into temperature stress response. Besides the genome, transcriptomes and limited scale of proteomes as well as metabolomes were created to enable an integrative interrogation of the interesting question—heat stress response.

We thank the reviewer for the positive feedback.

I am highly interested in the finding that P. cordatum has high GC content. Because it is even higher than that in the coral symbionts Symbiodiniaceae, it is clearly not a signature of heat resistance signature. I think this perspective deserves a mention and some discussion.

We have included this point in the main text but refrained from expanding the section too much given the already lengthy main text.

# *L376–381:* “Compared to dinoflagellates of Family Symbiodiniaceae well-known as the coral symbionts found in warm, sub-tropical coral reef ecosystems, *P. cordatum* is found in cooler, temperate waters. Therefore, the elevated G+C content of the *P. cordatum* genome does not appear to be a signature of heat resistance, and instead may be favored by selection to ensure high fidelity of transcription (*i.e.* G-C base pairs are more thermally stable) in open oceans.”

Another interesting finding is the greater number of genes being transcriptionally dynamic than previously reported/believed. This is consistent with recent studies on transcriptional regulation of nutrient related genes in various species of dinoflagellates.

The complementary trend of mRNA editing and alternative splicing as mechanisms of post- transcriptional regulation is also interesting.

The manuscript is reasonably well organized, despite some roughness or disconnect of flow as shown in specific comments shown below. The results are clearly described and the major conclusions are well supported. The methods are generally sound. I recommend acceptance after some minor/moderate revision.

Specific comments are provided in the following.

1. Line 95: reference 31 hypothesizes the recycling of cDNA into the genome, not on the occurrence of trans-splicing.

We have removed this citation (Slamovitz et al, 2008, Current Biology, 18: R550-R552) in this sentence.

2. Line 99: As far as I understand, P. cordatum is a globally distributed species, not an invasive species.

P. cordatum is invasive in the sense that it has expanded its habitat from the Caspian Sea where it was originally found (Khanaychenko et al. 2019) to coastal oceans worldwide, including temperate oceans e.g., the North Sea at Helgoland, where it is now regularly detected (Seebens et al., 2016). So it is globally distributed, but also invasive. We have clarified this in the text. See also our response to Reviewer 1’s comment above.

*L98–101:* “Among bloom-forming dinoflagellates, *Prorocentrum cordatum* (formerly

*Prorocentrum minimum*) [32, 33] is an invasive, potentially toxic species that has expanded its habitat from the Caspian Sea where it was originally found to coastal oceans worldwide [34], including the temperate oceans in the North Atlantic where it is now regularly detected [35].”

3. Lines 115-118: did you find the occurrence of introner elements in Polarella glacialis and A. gibbosum? If so, make a statement here; if not, this sentence is problematic.

Yes, this statement about the prevalence of introner elements (IEs) in free-living dinoflagellates is based on our analysis of all available genomes of free-living taxa, including Polarella glacialis and Amphidinium gibbosum. We identified IE-containing genes in ~11% of all genes in P. glacialis, and

~4% of all genes in A. gibbosum. These results are now presented in a revised Table S2. We made no changes to the text in this regard.

4. Lines 126-130: comparing "unidirectionally encoded" with "dispersed duplicates", and inferring "duplications events occurred independently" is confusing.

We have now clarified this in the text.

# *L130-133:* “Most genes are unidirectionally encoded in *P. cordatum* genome, as similarly observed in genomes of other dinoflagellates (Additional File 2: Fig. S4), e.g. [37]. Interestingly, a substantial proportion (64.8%) of the 85,849 genes in *P. cordatum* are dispersed duplicates (Fig. 1e and Additional File 3: Table S5), suggesting that ...”

5. Lines 134-139: The role of transposition in dinoflagellate genome evolution has been reported multiple times and relevant literature should be referred to here.

We have now cited three key references in this context and clarified this in the text. The cited references are Song et al. 2017 Genome Biology and Evolution 9: 2037-47; Gonzalez-Pech et al. 2021 BMC Biology 19:73; and Roy et al. 2023 Current Biology 33: 189-196.

# *L134-136:* “… alternatively, collinearity of duplicated blocks was disrupted by extensive rearrangements, due in part to the abundant transposable elements, as expected in dinoflagellate genomes [21, 52, 53].”

6. Lines 180-184 does not flow from the preceding sentence; it feels like jumping from one thing to the other, and cannot be connected by "For instance".

Here we refer to the specific example within component 1. We have revised the text to make this link more explicit for clarity.

# *L186–192:* “Our results also reveal both positive and negative correlations of expression between transcripts and proteins in component 1 (Fig. 3g) and component 2 (Additional File 2: Fig. S11). In component 1 for instance, gene expression of glutamine synthetase was positively correlated to the expression of chlorophyll *a*-*b* binding protein and the light- harvesting complex I LH38 proteins, whereas it was negatively correlated to protein expression of pyruvate dehydrogenase, and the sulfate and formate transporters.”

7. Line 330: Prorocentrum donghaiense has been renamed as Prorocentrum shikokuense.

We have updated this species name in both instances in the text, and the instance in Fig. S19.

*L343–345:* “We found, in the upstream region of eight gene models (Fig. 6d), the predicted leader sequence from the closely related *Prorocentrum shikokuense* [59].”

*L352–353:* “Highly similar sequence motifs present in *P. shikokuense* and other dinoflagellates support this hypothesis, …”

8. Lines 351-352: compared with the symbiotic and parasitic dinoflagellates, does P. cordatum have fewer introns along with longer introns that you describe? The trend of number of introns would be interesting to show in the paper.

Comparison of intron numbers between genomes is not straightforward, because these numbers are affected by the number of protein-coding genes and genome size. To compare introns of P. cordatum with other symbiotic and parasitic dinoflagellates, we opted to assess the distribution of intron lengths per genome instead. The distribution clearly shows that introns of P. cordatum are longer than those of other symbiotic or parasitic dinoflagellate species. These new results are now presented in the new Supplementary Figure S1. We cited this figure at the beginning of the Results section where the analysis of intron lengths is presented.

# *L117–119:* “The larger genome of *P. cordatum* encodes more protein-coding genes with longer introns than do the other species (Fig. 1e, Table 2, and Additional File 2: Fig. S1).”

**Fig. S1.** Distribution of intron lengths in genomes of *P. cordatum* and other dinoflagellates.

**Second round of review**

**Reviewer 1**

The authors have addressed all my previous comments. In my view the MS is now acceptable for publication. Please check the figure legends for a Euro symbol that has replaced panel (e).

**Reviewer 2**

I am satisfied by the authors' responses to my previous comments. I have no further comments. I think the manuscript is acceptable as is.
